# *Taraxacum kok-saghyz* (rubber dandelion) genomic microsatellite loci reveal modest genetic diversity and cross-amplify broadly to related species

**DOI:** 10.1038/s41598-019-38532-8

**Published:** 2019-02-13

**Authors:** Marcin Nowicki, Yichen Zhao, Sarah L. Boggess, Helge Fluess, Miriam Payá-Milans, Margaret E. Staton, Logan C. Houston, Denita Hadziabdic, Robert N. Trigiano

**Affiliations:** 1 Department of Entomology and Plant Pathology, The University of Tennessee, Knoxville, TN USA; 2 Guizhou Key Laboratory of Agro-Bioengineering, Guizhou University, Huaxi, Guiyang P. R. China; 3 Julius Kühn Institute for Breeding Research on Agricultural Crops, Sanitz OT Groß Lüsewitz, Germany; 4 Present Address: Centro de Biotecnología y Genómica de Plantas, UPM-INIA, 28223 Madrid, Spain

## Abstract

*Taraxacum kok-saghyz* (TKS) carries great potential as alternative natural rubber source. To better inform future breeding efforts with TKS and gain a deeper understanding of its genetic diversity, we utilized *de novo* sequencing to generate novel genomic simple sequence repeats markers (gSSRs). We utilized 25 gSSRs on a collection of genomic DNA (gDNA) samples from germplasm bank, and two gDNA samples from historical herbarium specimens. PCR coupled with capillary electrophoresis and an array of population genetics tools were employed to analyze the dataset of our study as well as a dataset of the recently published genic SSRs (eSSRs) generated on the same germplasm. Our results using both gSSRs and eSSRs revealed that TKS has low- to- moderate genetic diversity with most of it partitioned to the individuals and individuals within populations, whereas the species lacked population structure. Nineteen of the 25 gSSR markers cross-amplified to other *Taraxacum* spp. collected from Southeastern United States and identified as *T*. *officinale* by ITS sequencing. We used a subset of 14 gSSRs to estimate the genetic diversity of the *T*. *officinale* gDNA collection. In contrast to the obligatory outcrossing TKS, *T*. *officinale* presented evidence for population structure and clonal reproduction, which agreed with the species biology. We mapped the molecular markers sequences from this study and several others to the well-annotated sunflower genome. Our gSSRs present a functional tool for the biodiversity analyses in *Taraxacum*, but also in the related genera, as well as in the closely related tribes of the Asteraceae.

## Introduction

The growing human population has generated an increased demand for resources, including rubber, a substrate used for over 40,000 commercial products^1^ (Supplementary Fig. S1). Significant progress has been made in the production of synthetic rubber from non-renewable petroleum, and this increased its percentage in the total amount of the rubber supplied^1^. Yet, the vast majority of rubber production is still reliant on the same natural source from which it was initially discovered, the rubber tree *Hevea brasiliensis* Müll. Arg. (rubber palm^2,3^). This important crop is threatened by the South American leaf blight pathogen *Microcyclus ulei* (Henn.) Arx (syn. *Pseudocercospora ulei* [(Henn.) Hora Junior & Mizubuti, comb. nov. 2014]) and is losing competition for the land and the manpower against the economically favored African oil palm *Elaeis guineensis* Jacq^2,4^.

Natural rubber from plants outperforms that from petroleum in several aspects: the polymer of the natural rubber has much higher molecular weight compared with the synthetic rubber and the sustainable and renewable production of the plant (natural) rubber is considered superior to processing the non-renewable petroleum^1^. Several thousand plant species from across the world were screened for laticiferous properties, especially at times of increased rubber demand, e.g., WWI or WWII^5^ (Supplementary Fig. S1). The current body of scientific evidence points towards only a few species bearing the potential as an alternative to *H*. *brasiliensis* as a source of usable latex^1–3,5,6^. These species include guayule (*Parthenium argentatum* Gray), rubber ficus (*Ficus elastica* Roxb. ex Hornem.), and Russian dandelion (*Taraxacum kok-saghyz* Rodin; TKS). The molecular properties of rubber from each of these plants differ from those of the *Hevea* product^1–3,6^ and point toward specialty uses on species basis. For instance, the guayule rubber could be used for medical products because of the lower content of allergenic proteins^2^. The TKS rubber is of particular interest to the tire industry due to its high molecular weight (polymer index) and fast generation time (six months in TKS *vs*. seven years in *Hevea*), albeit with a comparatively higher content of allergenic proteins^1–3^. Moreover, each of these species could be grown in areas complementary to the *Hevea* palm (24°S through 23°N^7^) with latitudes reaching as high as temperate zones (*P*. *argentatum*: 21°N through 37°N; *F*. *elastica*: 10°S through 35°N; TKS: 35°N through at least 45°N).

TKS is of particular interest for the industry due to the proven success in production of tires^8^. The tire industry reported in their very first uses of TKS rubber that the tires “differed but little, according to their mechanical characteristics, from those made from imported natural rubber^9^” (citing^10^). In addition, it offers an accessory gain of inulin used in the manufacturing of numerous commercial products^11–14^. Both biosynthetic pathways are linked interchangeably within the TKS metabolism^13–15^. Several establishments devoted to TKS rubber production were founded in the United States (US) and Europe (Kultevat Inc., KeyGene Inc., ESKUSA GmbH^8^) emanating from the major research projects (project acronyms: EU-PEARLS; DRIVE4U^16^).

TKS is native to Kazakhstan^17^ and Western Xinjiang, China^18^, and is currently grown in Western Europe and North America alike (Kultevat Inc., KeyGene Inc., ESKUSA GmbH^8,16,19^). The plant was a major crop and model plant for rubber studies during the times of the Soviet Union of Socialist Republics (USSR). As hypothesized in other studies, likely due to the governmental pressure on performance, the TKS germplasm was profusely confused with the common dandelion species (*T*. *officinale* or *T*. *brevicorniculatum*). As a result, the world’s germplasm and botanical gardens collections were annotated as TKS for over 50 years despite being the common dandelions^20–23^. Recent United States Department of Agriculture - Agricultural Research Service (USDA-ARS) and European expeditions helped remedy this issue and provided new properly identified germplasm^22,24^. TKS is obligatory out-crossing, self-incompatible, diploid herbaceous plant (2n = 16), and morphologically resembles common dandelions, which exhibit mostly clonal reproduction due to polyploid genomic architecture^21,25^.

A recent spike in the TKS research confirmed the USDA-ARS collected species identity using the morphological^12,13,15,19,21^, molecular^14,21,25,26^, biochemical^11,12,14^, physiological^11,13,15^, and breeding^14,19,26^ approaches. The outcrossing nature of TKS was regarded when devising the plant genome linkage map^27^, followed by its genome sequence assembly to contigs^4^ and transcriptome sequencing^28^. All of this helped elucidate the TKS latex biosynthesis pathways^14,28,29^. In addition, a number of physiological and developmental studies on currently available germplasm provided data that was helpful in maximizing the rubber/inulin yield in both years the plants were grown^11,13,15,19^. TKS also proved amenable to genetic transformation and tissue culture^14,26^, indicating the potential for its breeding engineering and increases in yield of rubber^30,31^ or inulin^13–15^.

Although some progress has been made regarding TKS biology, physiology, and genetics, until recently only limited information was available regarding the species diversity and inheritance/interplay of traits of interest. McAssey *et al*.^32^ utilized the USDA-ARS TKS germplasm^24^ to estimate the genetic diversity of the species using 17 expressed-sequence tags/simple sequence repeats markers (genic SSR; EST-SSR; here dubbed as “eSSR”) mined from the available GenBank EST libraries, across 17 TKS populations from the species native area^24^. They concluded that the majority of the species diversity is captured within each population^32^. Similar conclusions were drawn from a study of Russian, American (USDA-ARS), and wild Chinese accessions of TKS using 23 eSSRs^33^. None of these studies utilized nuclear genomic short-sequence repeats markers (gSSRs) to infer the population structure and genetic diversity of this economically important plant species. Moreover, the available TKS genome assembled to the contigs level only^4^ is lacking an extensive annotation or higher-level organization, despite providing insights into the TKS rubber/inulin biosynthetic pathways.

The goal of our study was to infer the TKS population structure, information of high value for breeding of this potential industrial crop. We utilized *de novo* sequencing to generate novel TKS gSSRs and to estimate the genetic diversity and spatial structure of the USDA-ARS TKS germplasm. We hypothesized that the majority of the species diversity would be captured in each examined population, as found in prior studies that utilized eSSRs^32,33^. The specific research objectives included the following: (1) identifying and characterizing polymorphic gSSR loci using *de novo* sequence of the TKS genome and mapping of the useful polymorphic gSSRs and other marker sequences onto the well-annotated genome of the related species *Helianthus annuus*^4,27,32^; (2) estimating the genetic diversity and inferring the population structure of the USDA-ARS TKS germplasm^24^ and two available historical herbarium TKS samples using gSSRs; and (3) comparing the gSSR data with the published eSSR data of McAssey *et al*.^32^ and Yushuang *et al*.^33^ to reach better-informed conclusions on TKS genetic diversity. We then deployed those gSSRs in a cross-amplification study with the local US dandelion samples (*T*. *officinale*), including their molecular identification. Information provided here will be useful in advancing future TKS studies, in the current and future breeding efforts of this potential crop for renewable rubber, and in augmenting the currently available resources for analyses of *Taraxacum* spp. and related plants.

## Results

### Designing and validating gSSR-markers

#### TKS SSRs discovery and the marker map

The TKS *de novo* genome sequencing yielded 45,804,966 paired-end reads of 275 bp. After trimming, 42,367,598 reads with a mean length of 265 bp were masked and used for *de novo* assembly on ABySS. The resulting *de novo* assembly contained 8,077,494 unitigs, from which 99,429 SSRs were identified on 95,692 sequences. From these, 11,259 were compound SSRs, meaning that two SSRs were separated by at most 15 bp. Primers were computed for 22,764 perfect SSRs. The number of SSRs with primers were 15,760 for the di-, 4,893 for the tri-, and 2,111 for the tetranucleotides, respectively.

**Figure 1:**
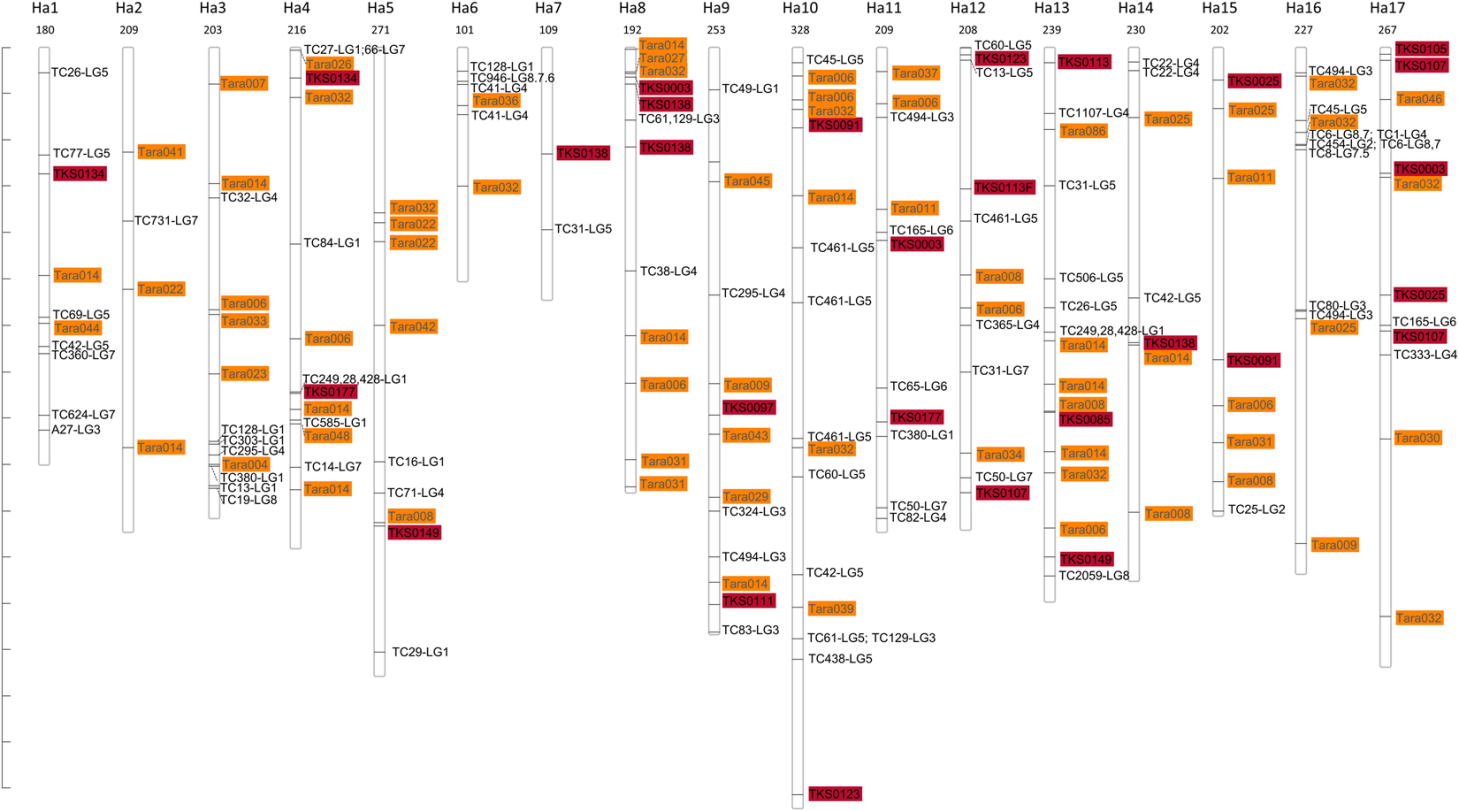
Schematic map of *Taraxacum kok-saghyz* (TKS) markers on the *Helianthus annuus* genome. Sequences of the markers were retrieved by searching the TKS genome contigs^4^, and ~1 kb sequences containing the markers of Arias *et al*.^27^, McAssey *et al*.^32^ and our *de novo* gSSRs were used to search the sunflower genome using their BLAST algorithm^73^. Physical positions of the best hits, occasionally located to several positions of the sunflower genome with comparable reliability, are shown. Markers used to generate the TKS linkage map^27^ are black on white plates with their original linkage groups (LG) markings; eSSRs of McAssey *et al*.^32^ are black on red plates; our *de novo* gSSRs are gray on orange plates. The *H*. *annuus* chromosomes are numbered on top with their respective sizes [Mbp] indicated. Scale on the left ticks every 20 Mbp.

Because of the lack of a well-annotated TKS genome, the ~1 kb sequences pulled from the TKS contigs of Lin *et al*.^4^ containing the markers used for the construction of TKS linkage map^27^ and those of the gSSR and eSSR^32^ population genetics studies were mapped to the sunflower genome. The markers analyzed were located across all eight TKS linkage groups based on the mapping back to the sunflower genome (Fig. 1). In several instances, the marker sequences localized to separate TKS contigs, but the sequences mapped to the same sunflower genome regions (Fig. 1). Only one of our gSSRs (Tara026) co-localized with two other map markers (TC27; TC66) within a single TKS genome scaffold, and also mapped back close to each another on the sunflower genome (Fig. 1).

#### SSR genotyping and analyses

We chose a pool of 25 di- and 25 tri-nucleotide repeat gSSR markers for the study of TKS germplasm (Table 1 and Supplementary Table S1). After their initial screening on the TKS gDNA, this gSSR pool was reduced as several did not amplify a significant number of gDNA samples of the collection, lacked polymorphic alleles, or amplified a more complex banding pattern. We thus chose the 25 best-performing gSSRs for their specificity (single or double PCR products only) and reproducibility, and used them for TKS population genetics studies (Table 2 and Supplementary Table S1).

**Table 1:** Population genetics indices of *Taraxacum kok-saghyz* (TKS) populations.

TKS population^a^	N^b^	N_A_/population^b^	MLG^b^	eMLG^b^	H_O_^b^	H_E_^b^	F^b^
35156	3	50	3	3	0.33	0.39	0.22 ^ns^
35159	3	60	3	3	0.45	0.54	0.49***
35160	3	66	3	3	0.50	0.61	0.26**
35162	4	63	4	4	0.49	0.57	0.44***
35164	3	61	3	3	0.44	0.53	0.34***
35165	3	55	3	3	0.37	0.45	0.23*
35166	3	57	3	3	0.40	0.48	0.22*
35168	3	53	3	3	0.35	0.43	0.15 ^ns^
35169	3	69	3	3	0.53	0.64	0.31***
35170	3	59	3	3	0.41	0.49	0.23*
35172	3	55	3	3	0.36	0.44	0.10 ^ns^
35173	3	56	3	3	0.37	0.45	0.06 ^ns^
35176	3	57	3	3	0.42	0.50	0.33**
35177	3	51	3	3	0.33	0.40	0.28*
35178	5	49	5	5	0.55	0.47	−0.01 ^ns^
35180	3	67	3	3	0.45	0.54	0.39***
35181	3	71	3	3	0.52	0.63	0.33***
35182	3	61	3	3	0.48	0.54	0.31**
35183	3	63	3	3	0.46	0.56	0.32**
Herbarium	2	34	2	2	0.38	0.42	0.44 ^ns^
**Overall**	**62**	**57.85**	**62**	**10**	**0.38**	**0.54**	**0.29**

^a^Population description, as per USDA-ARS^24^. Herbarium: two TKS herbarium specimens, MONT 51683 (H.E. Morris, September 11, 1942) and KE 650 (C. Hobbs, July 02, 1949) submitted for destructive sampling.

^b^Diversity indices calculated: N: Number of individuals tested per population; N_A_/population: Total number of alleles detected per population (after binning); MLG: Number of Multi-Locus Genotypes (MLGs) detected; eMLG: Number of MLGs expected; H_O_: Observed heterozygosity; H_E_: Expected heterozygosity (Nei’s unbiased gene diversity^91^); F: Fixation inbreeding coefficient ([1− (H_O_/H_E_)], with significance tested by 1,000 permutations of the dataset: ^ns^not significant; ^*^P < 0.05; ^**^P < 0.01;^***^P < 0.001).

#### TKS: Population genetics analyses

gSSRs: Analysis of TKS spatial fixation genetics indices, Multi-locus genotype (MLG) networks, and population structure: Our results suggest no significant deviations from the Hardy-Weinberg equilibrium (HWE) across the 25 gSSR markers used to analyze the TKS populations (Supplementary Fig. S2) despite low sampling. Only the TKS population 35162, and to a lesser extent 35178, show deviations from HWE at six and two loci, respectively. All loci were polymorphic in each population tested, and no clonal MLGs were detected. The Genotype Accumulation Curve (GAC) analyses indicated an MLG saturation (Supplementary Fig. S3) with eight gSSRs in the analyzed TKS germplasm. Analyses of the Index of Association (Ia) confirmed the outcrossing character of the TKS germplasm studied (Supplementary Fig. S4). Only modest linkage disequilibrium was found in the gSSR TKS dataset (Supplementary Fig. S5) and suggested well-dispersed genomic locations of the gSSRs used.

The amplified gSSR markers yielded from 3 to 13 alleles per locus, averaging about 6 across the TKS germplasm pool (Table 2). The 25 gSSRs used indicated a moderate degree of inbreeding within the populations and overall (F_IS_ = 0.287; Tables 1 and 2). Our results further indicated a moderate TKS population fixation and genetic differentiation across the 25 loci tested (F_ST_ = 0.094; F’_ST_ = 0.098; Table 1). This implied a moderate level of gene flow among the TKS populations (inferred N_m_ = 2.41). Collectively, the data indicated rather low genetic differentiation among the TKS datasets analyzed, despite high allelic diversity of the obligatory outcrossing TKS. In agreement with the spatial fixation indices accrued, AMOVA for the gSSR dataset indicated the majority of the molecular diversity partitioned among the individuals and not among populations (Φ_IT_ = 66.52%; Φ_IS_ = 23.63%; Φ_ST_ = 9.86%).

**Table 2:** List of the *Taraxacum kok-saghyz* (TKS) genomic short sequence repeat (gSSR) markers developed in the study and summary statistics across 20 TKS populations.

gSSR locus	GenBank accession #	Contig as per^4^	Motif	Allele size range [bp]	% PCR successful^a^	N_A_^b^	H_O_^b^	H_E_^b^	F_IS_^b^	F_ST_^b^	F’_ST_^b^	Cross-amplification^c^
Tara003	MH397372	(not detected)	(AT)_7_	256–269	97	6	0.10	0.45	0.78	0.00	0.00	yes*
Tara004	MF033818	utg5180; utg10022	(TG)_6_	215–230	97	5	0.03	0.30	0.89	0.00	−0.02	yes*
Tara006	MH397373	utg1153; utg145; utg19593	(AT)_6_	379–386	74	4	0.00	0.61	1.00	0.01	0.01	no
Tara007	MH397374	utg3858; utg2509	(GA)_6_	355–360	92	3	0.00	0.29	1.00	0.00	−0.25	yes*
Tara008	MH397375	utg2139; utg1303; utg17621; utg3133	(CT)_6_	105–119	98	7	0.67	0.57	−0.18	0.09	0.10	no
Tara011	MH397376	utg8509	(CT)_6_	286–301	84	5	0.07	0.47	0.84	0.16	0.17	yes*
Tara014	MH397377	utg30583	(TA)_7_	176–194	95	9	0.31	0.44	0.29	0.20	0.21	yes*
Tara022	MH397378	utg34485	(AAG)_8_	188–207	97	8	0.40	0.65	0.39	0.09	0.10	yes
Tara023	MH397379	utg3340	(CAT)_6_	236–252	97	5	0.62	0.63	0.02	0.10	0.11	no
Tara026	MF033819	utg12026	(CTT)_10_	340–380	90	13	0.70	0.86	0.19	0.04	0.04	yes*
Tara027	MF033820	utg1422	(ATA)_6_	375–391	87	5	0.07	0.35	0.80	0.10	0.11	yes*
Tara029	MH397380	utg12556	(TGG)_6_	329–364	94	5	0.88	0.62	−0.42	0.03	0.04	yes*
Tara030	MF033821	utg9893	(TAA)_7_	360–369	87	4	0.00	0.23	1.00	0.10	0.11	yes*
Tara031	MH397381	utg2986	(ATC)_7_	387–392	90	3	0.10	0.13	0.20	0.14	0.14	yes*
Tara032	MF033822	utg15094; utg27973	(AGA)_6_	191–263	100	4	0.99	0.64	−0.55	0.06	0.06	yes*
Tara033	MH397382	utg7101	(AGA)_6_	157–346	95	5	1.00	0.70	−0.43	0.05	0.06	yes
Tara036	MH397383	utg21567	(CAT)_7_	295–308	98	6	0.18	0.72	0.76	0.10	0.10	yes
Tara037	MH397384	utg3890	(AGA)_9_	242–286	92	9	0.58	0.61	0.05	0.20	0.21	yes
Tara039	MH397385	utg14580	(ACA)_7_	113–129	98	7	0.21	0.74	0.72	0.01	0.01	no
Tara041	MH397386	utg19083	(GTT)_6_	109–130	97	7	0.81	0.64	−0.27	0.00	−0.03	yes
Tara042	MF033823	utg10767	(AAC)_6_	217–225	95	4	0.28	0.55	0.48	0.20	0.21	yes
Tara043	MF033824	utg4473	(CTC)_7_	225–341	97	6	0.30	0.56	0.46	0.09	0.09	yes*
Tara045	MF033825	utg17775; utg13920; utg13797	(TAA)_12_	128–150	95	6	0.33	0.45	0.28	0.35	0.36	yes*
Tara046	MH397387	utg9231	(CAA)_7_	220–236	90	6	0.37	0.67	0.45	0.07	0.08	yes*
Tara048	MH397388	utg12243	(TTC)_7_	224–239	95	6	0.62	0.67	0.09	0.14	0.15	yes
**Mean**					**93**	**5.92**	**0.38**	**0.54**	**0.29**	**0.09**	**0.10**	

^a^Percentage of successfully amplified TKS gDNA samples, from a total of *n* = 62 individuals.

^b^Diversity indices calculated: N_A_: Number of alleles per locus (after binning); H_O_: Observed heterozygosity; H_E_: Expected heterozygosity (Nei’s unbiased gene diversity^91^); F_IS_: Deviation from panmictic HWE breeding model (Inbreeding coefficient); F_ST_: Co-ancestry coefficient (effect of population sub-division); F’_ST_: Standardized F_ST_ (the proportion of total variance explained by genetic differentiation among populations relative to the maximum proportion attainable, given the observed variation within populations).

^c^Results of cross-amplification study to US dandelions gDNA collection: “no”: The locus did not cross-amplify; “yes”: The locus cross-amplified, but marker was not used in the US dandelion population study (erratic amplification, complex banding pattern); “yes*”: The locus cross-amplified and the marker was used in the US dandelion population study.

**Figure 2:**
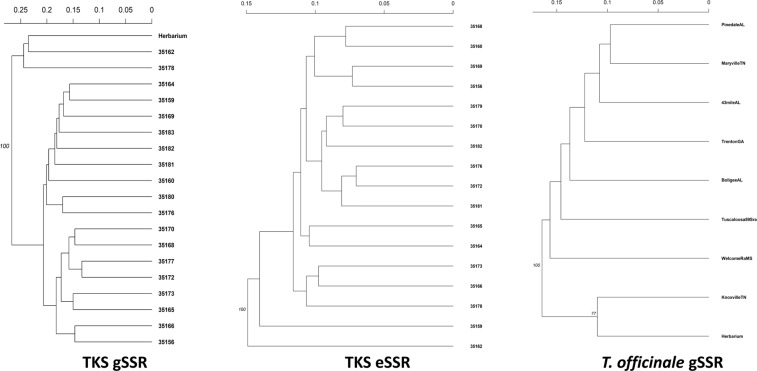
*Taraxacum* spp. genetic diversity. Neighbor-joining tree of genetic distances^88,92^ between the *T*. *kok-saghyz* (TKS) populations used in this study captured with gSSRs (left panel), TKS eSSRs dataset of McAssey *et al*.^32^ (middle panel), and US *T*. *officinale* using chosen gSSRs of this study (right panel). Neighbor-joining trees were generated using the Prevosti algorithm^93^ with 1,000 permuted randomizations. Bootstrap support exceeding 70% is indicated. The computed genetic distance scales are placed on top of each respective tree.

**Figure 3:**
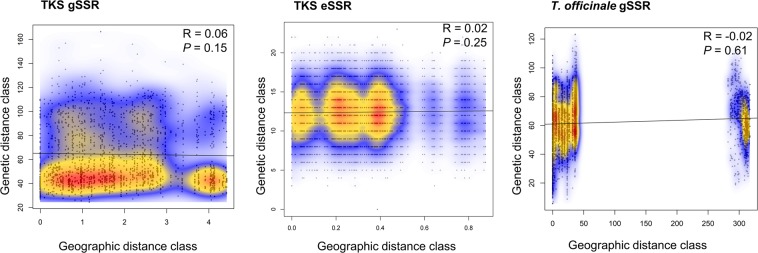
Mantel test of the correlation of geographic distance and genetic distance matrices for the *Taraxacum kok-saghyz* (TKS) gSSR dataset (left panel; two Herbarium samples removed), TKS eSSR dataset of McAssey *et al*.^32^ (2016; middle panel), and US *T*. *officinale* using 14 gSSRs (right panel) were analyzed using 1,000 permutations. Mantel’s R indices and their corresponding statistical support are indicated, respectively.

To compare the relatedness of both TKS datasets (gSSRs and eSSRs), we generated pairwise matrices of population genetic distances for both. Values of the population pairwise distance matrix of F_ST_ ranged from 0.018 to 0.355 for the gSSR, and from −0.024 to 0.261 for the eSSR datasets, respectively (data not shown). The pairwise population F_ST_ distance matrices (and D_ST_ matrices; both unstandardized and standardized; data not shown) for the gSSR and eSSR datasets provided similar results (Supplementary Fig. S6), thus indicating that the TKS diversity information was comparable between them. Sub-population-wise, the distance matrix for the gSSR dataset showed low resolution in the analyzed TKS collection (Fig. 2; Prevosti genetic distance range: 0.004 to 0.244, averaging 0.076 ± 0.056). The Neighbor-Joining dendrogram built on this basis indicated three TKS sub-populations as outliers (Herbarium, 35162, and 35178), and the remaining ones possibly divided into two separate clades. Testing of the geographic distance among TKS populations driving the genetic diversity of the species proved inconclusive (Fig. 3).

**Figure 4:**
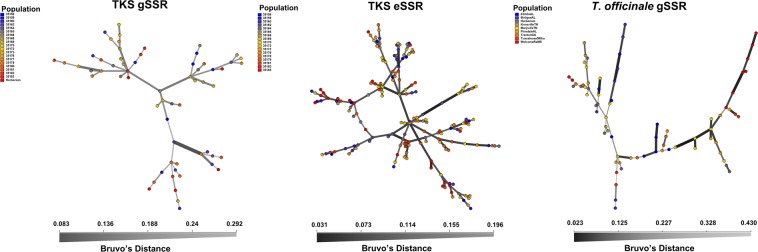
Minimum spanning networks for the *Taraxacum kok-saghyz* (TKS) gSSR dataset (left panel), TKS eSSR dataset of McAssey *et al*.^32^ (middle panel), and US *T*. *officinale* dataset using 14 gSSRs selected for this study (right panel). Bruvo’s distance (considering the motifs lengths) was used to reticulate the datasets. Color legends for the populations and Bruvo distance scales/shading are indicated, respectively, on each graph.

**Figure 5:**
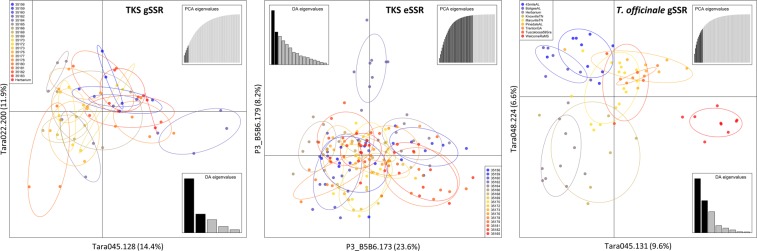
Discriminant Analysis of Principal Components (DAPC) for the *Taraxacum kok-saghyz* (TKS) gSSR dataset (left panel), TKS eSSR dataset of McAssey *et al*.^32^ (middle panel), and US *T*. *officinale* using 14 gSSRs selected for this study (right panel). Optimized and cross-checked PCA eigenvalues were used to generate each graph, respectively (gSSR: 5 PCAs retained; eSSR: 39; *T*. *officinale* gSSR: 11). Color legends for the populations and DA/PCA eigenvalues used are shown, respectively, on each graph. Alleles contributing the most to explaining the variance for each dataset are indicated on either axis, respectively (with percentages of the variance explained in the parentheses, respectively).

Reticulation analyses of the gSSR MLGs using the Minimum Spanning Networks (MSN) supported the F-statistics conclusions with no evidence of population structure in the TKS germplasm analyzed with gSSRs (Fig. 4). The lack of clustering or population structure visualized this way, suggests species-wide gene flow, implying that TKS diversity is well retained at the sub-population level. The results of the Discriminant Analysis of the Principal Components (DAPC; Fig. 5) were in agreement with the F_ST_ population-wise trees (Fig. 2), with the gSSR populations 35162, 35178, and the Herbarium samples placed with some distance to the majority of the remaining samples.

eSSRs: Comparative analysis of TKS spatial fixation genetics indices, Multi-locus genotype (MLG) networks, and population structure: In the re-analyzed eSSR TKS dataset^32^, the significant deviations from HWE presented a locus-wise pattern and were much more common in occurrence than in the gSSR dataset (Supplementary Fig. S2). The eSSRs saturated the MLGs detected in the TKS germplasm significantly slower than gSSRs (10 vs. 8 markers, respectively; Supplementary Fig. S3). The eSSR dataset provided congruent results with the gSSR dataset on Ia and pairwise linkage disequilibrium (Supplementary Figs S4 and S5). Regarding the fixation indices, the eSSR dataset harbored an overall F_ST_ = 0.11^32^ and F’_ST_ = 0.068 (data not shown), and an inferred N_m_ = 2.02. Partitioning of the molecular variance with AMOVA for the eSSR dataset yielded results similar to the gSSR dataset (Φ_IT_ = 84.61%; Φ_IS_ = 8.34%; Φ_ST_ = 7.04%). Differences occurred in the variance partitioned among individuals within populations, and the gSSR dataset showed higher value of this parameter than the eSSR dataset. The F_ST_ distance matrix for the eSSR dataset of TKS showed different population-wise clustering from the gSSR dataset (Fig. 2). The eSSR study showed marginally higher resolution in the pairwise genetic distances of TKS populations, likely due to a much higher number of samples per population analyzed (Prevosti distance range: 0.003 to 0.149, averaging 0.099 ± 0.082). Similar to the gSSR dataset, the sub-population 35162 was separated with high confidence from the bulk of other sub-populations, as was 35159. The absolute placement of the eSSR sub-populations differed from the gSSR dataset and indicated generally better resolution than the gSSR dataset, but no major clustering. Testing of the geographic distance among TKS populations driving the genetic diversity of the species proved inconclusive, similar to the gSSR dataset (Fig. 3).

MSN reticulation of the eSSR dataset (Fig. 4) provided results similar to the gSSR dataset, confirming that study’s conclusions^32^ of TKS lacking well-defined population structure. Analysis of networks from both gSSR and eSSR datasets resulted in similar Bruvo’s genetic distance ranges, and congruently implied lack of TKS population structure. Similar to the gSSR dataset, the DAPC analysis of the eSSR dataset (Fig. 5) also confirmed the sub-population-wise tree of genetic distances (Fig. 2). The eSSR population 35162 presented a similar (diverged) pattern to this observed in the gSSR dataset. Overall, our results suggest a lack of a well-defined population structure of the TKS germplasm with little support for the more differentiated population 35162.

#### *Analyses of US* Taraxacum officinale

**Figure 6:**
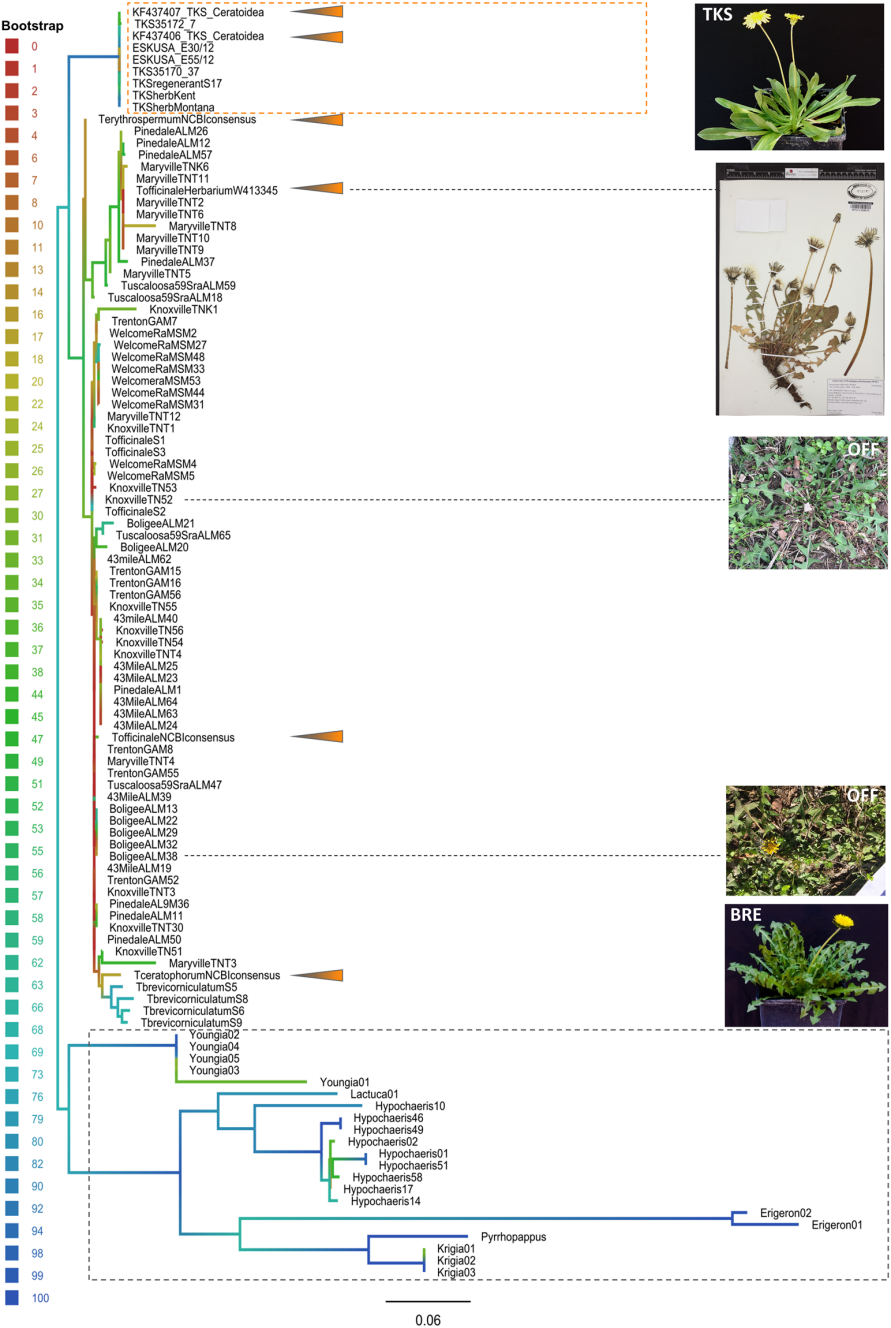
Phylogenetic relationships between dandelions used in the study. The ITS sequences of *Taraxacum kok-saghyz* (TKS), other *Taraxacum* species of interest, US dandelions, and outgroup specimens from Southeastern US were aligned using MAFFT^62,63^, trimmed with SeaView-Gblocks^65^ to remove the uninformative characters, and used for thorough-bootstrap RAxML^66^ analysis (100 runs with 10,000 repeats, rapid hill-climbing mode, GTR substitutions, multiple outgroup), over 451 distinct alignment patterns. The Gblocks regions alignment was 660 bp long. Proportion of gaps and completely undetermined characters in this alignment: 3.39%. The resultant tree was built using FigTree, and the branches are colored as per the bootstrap support (indicated on the color legend). Arrows indicate positions of sequences pulled from NCBI (*T*. *ceratophorum*: consensus of three ITS sequences deposited; *T*. *erythrospermum*: consensus of 12 ITS sequences; *T*. *officinale*: consensus of 49 sequences; Supplementary Table S2), of the historical *T*. *officinale* specimen (TofficinaleW413345: WTU 413345; picture published with permission of the Burke Museum, University of Washington) and the historical specimens of TKS (TKSherbKent: KE 650; TKSherbMontana: MONT 51683), or other species^34^ (for TKS, *Ceratoidea*). No ITS sequences for *T*. *brevicorniculatum* were found at NCBI. The sample origin (population names) or *Taraxacum* species names are indicated. Dotted grey box delimits the out-group for RAxML (non-*Taraxacum* species by ITS BLAST of sequences); orange box indicates the TKS. Sample ESKUSA E55/12 was used for *de novo* sequencing and development of the gSSRs used in this study. Pictures of exemplary specimens show TKS, *T*. *brevicorniculatum* (BRE), and *T*. *officinalis* (OFF; the dashed lines are indicating which specimens are shown). Samples of TKS, *T*. *officinale*, and *T*. *brevicorniculatum* marked with S were grown for another study^26^.

Species genotyping and assignment and ITS phylogeny of the plant materials: Species identity of the samples collected from Tennessee, Georgia, Alabama, and Mississippi (Tables 3 and S1) was confirmed by Internal Transcribed Spacer region (ITS) sequencing (Fig. 6; Supplementary Tables S1 and S2). Samples identified as *Taraxacum* spp. lacked major differences in their ITS sequences (Fig. 6) and could not be unambiguously classified at species level based on this criterion alone (NCBI BLAST; data not shown). Grouping with the *T*. *officinale* and other *Taraxacum* species sequences for ITS^34^ and NCBI consensi (Supplementary Table S2) did not resolve our collection into distinct species (Fig. 6 and Supplementary Files S2). Therefore, based on that non-resolution and due to the plants sharing major morphologic similarities, we treated those samples as a presumptive *T*. *officinale* collection. ITS sequencing also identified a number of outgroup specimens, morphologically resembling the *T*. *officinale* but from distant genera such as Y*oungia* (*Y*. *japonica*; GU724281.1; 99% ITS sequence identity over 100% coverage), *Hypochaeris* spp. (several species hit with 99% and higher identity over 99% and higher coverage), *Krigia* spp. (L13945.1; 98% identity over 100% coverage), *Lactuca* (*L*. *canadensis*; GU818575.1; 99% identity over 99% coverage), *Pyrrhopappus* (*P*. *carolinianus*; AY218955.1; 99% identity over 90% coverage), and *Erigeron* (*E*. *annuus*; EF107653.1; 99% identity over 100% coverage, *E*. *philadelphicus*; AF046989.1; 99% identity over 90% coverage).

**Table 3:** Population genetics indices of the US dandelion (*Taraxacum officinale*) populations.

US dandelion sub-population^a^	GPS^b^	N^c^	N_A_/population^c^	MLG^cb^	Ploidy estimated by gSSRs^d^	H_O_^c^	H_E_^c^	F^c^
43mileAL	32.844146; −87.951552	9	78	9	3×; 4×	0.02	0.97	0.97**
BoligeeAL	32.798746; −88.032085	7	59	7	4×	0.03	0.95	0.95**
Herbarium	(Supplementary Table S1)	10	75	10	3×; 4×	0.04	0.93	0.93**
KnoxvilleTN	(Supplementary Table S1)	6	55	6	2×; 3×; 4×	0.16	0.73	0.73*
MaryvilleTN	(Supplementary Table S1)	16	93	16	2×; 3×; 4×	0.09	0.86	0.86**
MSwelcomeRA	32.411028; −88.533979	9	60	9	4×	0.00	1.00	1.00**
PinedaleAL	33.851114; −86.306398	8	60	8	4×	0.09	0.86	0.86**
TrentonGA	34.869886; −85.513952	5	66	5	3×; 4×	0.00	1.00	1.00**
Tuscaloosa59Sra	33.174298; −87.448362	4	59	4	4×	0.00	1.00	1.00*
**Overall**		**74**	**605**	**74**		**0**.**05**	**0**.**71**	**0**.**93**

^a^Dandelion population description, as per sampling locations (also see Supplementary Table S1). Herbarium: Specimens of *T. officinale* submitted for destructive sampling.

^b^Locality of the given collection site, by GPS coordinates. In cases when multiple localities were sampled for a given collection site, the per-sample details are listed out in the Supplementary Table S1.

^c^Diversity indices calculated: N: Number of individuals tested per population; N_A_/population: Total number of alleles detected per population (after binning); MLG: Number of Multi-Locus Genotypes (MLGs) detected; H_O_: Observed heterozygosity; H_E_: Expected heterozygosity (Nei’s unbiased gene diversity^92^); F: Fixation inbreeding coefficient ([1− (H_O_/H_E_)], with significance tested by 1,000 permutations of the dataset: ^*^P < 0.05; ^**^P < 0.01). Due to the mixed-ploidy character of this dataset, we were unable to calculate the standard fixation indices.

^d^Ploidy of specimens in a given collection site, estimated by the allele numbers detected with the chosen 17 gSSRs (also see Supplementary Table S1).

Analysis of *Taraxacum officinale* spatial fixation genetics indices, Multi-locus genotype (MLG) networks, and population structure: The majority of the TKS-derived gSSRs cross-amplified the gDNA of the related US native dandelions (*T*. *officinale*) and of the outgroup specimens (Tables 2, 4 and S1). From the 25 gSSRs tested using the TKS gDNA collection, 21 gSSRs (five di- and 16 tri-nucleotide repeats) cross-amplified to the *T*. *officinale* gDNA collection as confirmed on four gDNA samples (Knoxville, TN population). Overall, the cross-amplification was broad and proved effective even in the specimens of related genera and tribe (Supplementary Table S1).

The analyses of the species diversity and population structure included 74 samples of *T*. *officinale* collected in several locations in the US using the 14 best-performing gSSRs (five di- and nine trinucleotide repeats) developed for TKS. Our results indicated violations of HWE in both locus- and population-manner (Supplementary Fig. S2). The MLG accumulation in this dataset was comparatively the slowest among all the datasets analyzed as 13 gSSRs saturated the genotype accumulation curve (Supplementary Fig. S3). Moreover, the index of association (Ia) was typical for clonal/asexual organisms (Supplementary Fig. S4; *P* = 0.051). Linkage disequilibrium range for this dataset was similar to that of the gSSR study of TKS (Supplementary Fig. S5) with the difference of fewer and smaller negative values recorded for the *T*. *officinale* dataset. As expected, the ploidy of the apomictic *T*. *officinale* samples estimated by the number of detected alleles often reached the tetraploid levels (diploid, *n = *4; triploid, *n = *17; tetraploid; *n = *53; Supplementary Table S1), which limited the scope of the population genetics analyses, in particular the *F*-statistics (fixation indices analyses). To gain access to that data, we coded the whole dataset as tetraploid with occasionally missing alleles and corrected the ploidy with the R package *polysat* before analyses.

**Figure 7:**
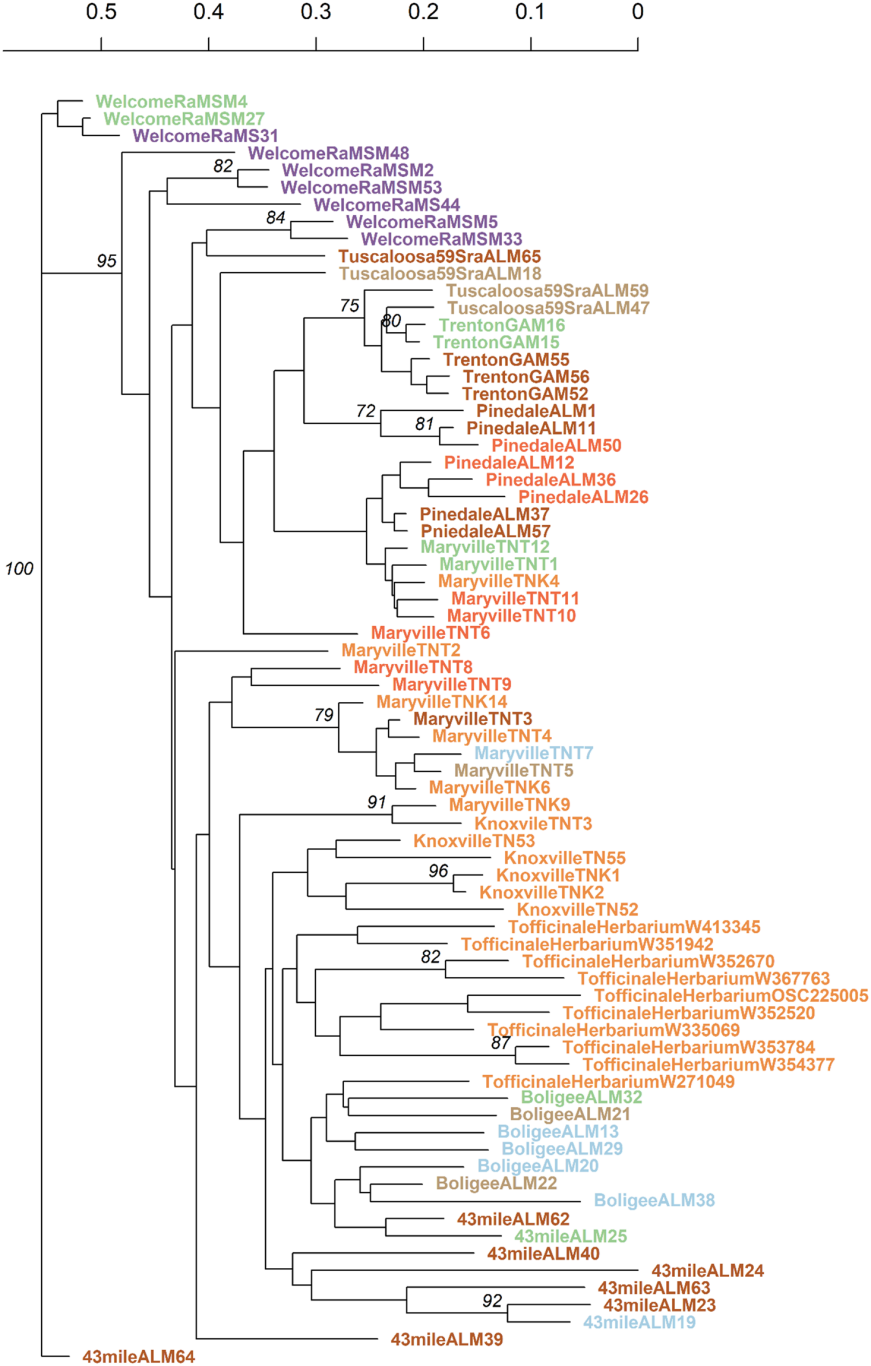
Phylogenetic relationships among the US *Taraxacum officinale* using 14 gSSRs selected for this study. Bruvo’s distances among the specimens were calculated to generate the FastME tree^94^ (1,000 permutations; bootstrap support of 70% and more is indicated). The dandelion individuals were color-coded as per the Bayesian Information Criterion in R package *poppr*^78,79^ (K-means hierarchical clustering; K_min_ = 7).

The *T*. *officinale* dataset displayed between 5 and 16 alleles per locus (averaging about 10; Table 4). The estimated dataset-wide F_ST_ value was 0.044 and the D’_ST_ was 0.048. Population-wise F_IS_ values (Table 3) indicated a considerable degree of homozygote excess in this dataset, further supporting the conclusion of asexual reproduction in this species. The population-wise Prevosti distance tree for *T*. *officinale* indicated its genetic distances were lower than TKS using the same markers (range: 0.008 to 0.157, averaging 0.055 ± 0.045; Fig. 2), indicating the lowest resolution in this dataset among those analyzed in the study. Further, the majority of the tree remained unresolved, with the samples from Herbarium (US western coast) and KnoxvilleTN forming an outgroup to the bulk of the dataset yet separated from one another. Similar separation was observed when analyzing the genetic and geographic distance matrices using the Mantel test (Fig. 3). Herbarium samples from the US western coast clustered separately from the remaining individuals based on the geographic spacing (Fig. 3). The majority of the molecular variance was retained among the individuals within the populations, whereas about one quarter of the total variance was partitioned among the populations (AMOVA: Φ_IS_ = 74.98%; Φ_ST_ = 25.02%). Several analyses indicated the presence of population structure in this dataset. The MSN analyses (Fig. 4) took into account motif lengths in the gSSRs and grouped individuals of several populations together using the Bruvo distance. In agreement with the population-wise tree of distances (Fig. 2), the DAPC analyses separated the WelcomeRaMS, as well as the Herbarium and KnoxvilleTN samples from the bulk of the remaining ones (Fig. 5). Comparatively larger resolution of this dataset than either of the TKS datasets suggested more pronounced population structure in the *T*. *officinale*, as (sub-)populations are more diverged from one another than in TKS. Bruvo’s distance-based tree of individuals (Fig. 7; motif lengths considered) was visualized using the Bayesian Information Criterion and grouped individuals from the geographically close populations together yet further implying population structure in the common dandelion species. Collectively, the results for *T*. *officinale* indicated the existence of low-diversity populations clonal in character but differentiated geographically.

**Table 4:** The US *Taraxacum officinale* and summary statistics, using the *T*. *kok-saghyz* (TKS) gSSR markers.

gSSR locus	% PCR successful^a^	N_A_^b^	1−D^b^	H_O_^b^	H_E_^b^	Evenness
Tara003	93	12	0.72	1	0.75	0.65
Tara004	100	16	0.88	1	0.88	0.75
Tara007	99	8	0.67	1	0.74	0.66
Tara011	96	10	0.76	1	0.67	0.68
Tara014	53	5	0.65	1	0.65	0.82
Tara026	100	12	0.59	1	0.74	0.54
Tara027	92	10	0.69	1	0.67	0.57
Tara029	91	7	0.63	1	0.64	0.70
Tara030	91	11	0.81	1	0.69	0.73
Tara031	89	9	0.70	1	0.67	0.73
Tara032	100	10	0.75	1	0.78	0.79
Tara043	100	7	0.41	1	0.63	0.54
Tara045	89	12	0.82	1	0.84	0.76
Tara046	72	15	0.81	1	0.79	0.76
**Mean**	**91**	**10**.**3**	**0**.**71**	**1**	**0**.**70**	**0**.**69**

^a^Percentage of successfully amplified TKS gDNA samples, from *n* = 74.

^b^Diversity indices calculated: N_A_: Number of alleles per locus (after binning); 1-D: Simpson’s Diversity index; H_O_: Observed heterozygosity; H_E_: Expected heterozygosity (Nei’s unbiased gene diversity^92^); Evenness: A measure of genotypes distribution within a population. Due to the mixed-ploidy character of this dataset, we were unable to calculate the standard fixation indices.

## Discussion

In this study, we aimed to gain a deeper understanding of the genetic diversity of TKS, a potential alternative, sustainable rubber crop^2,13,14^. To reach this goal, we developed a set of genomic SSRs (gSSRs) based on our *de novo* sequencing of TKS and utilized them for evaluating the genetic diversity of TKS germplasm. We then carried-out an array of comparative population genetics analyses, re-analyzing the recently published genic SSR (eSSR) dataset generated on the same TKS germplasm^32^, and an expanded cross-amplification study with the local US dandelions using those gSSRs.

Our *de novo* gSSRs were distributed across the TKS genome, based on the linkage disequilibrium data, as were the eSSRs^32^. We mapped both types of SSRs (gSSRs and eSSRs) along with the TKS markers used for the linkage map, back to the related and well-annotated *H*. *annuus* genome, based on the TKS contigs^4^. This is very likely to be helpful for the future breeding efforts. We chose not to use the TKS genome assembly^4^ or the closely related *Lactuca sativa* genome assembly^35^ because both are more fragmented and have fewer scaffolds anchored to chromosome locations in comparison to the *H*. *annuus* genome. To further underscore the need for improved TKS genome resources, the gSSR Tara003 sequence could not be found in the TKS contigs published^4^. Moreover, only 15 markers out of the 65 that constructed the TKS linkage map^27^ were mapped back together (in pairs or in threes) to six TKS scaffolds of Lin *et al*.^4^. Also, only one of the SSRs analyzed (gSSR Tara026) co-localized with two other map markers of Arias *et al*.^27^ within a single TKS contig of Lin *et al*.^4^. Several studies independently reported the TKS genome size as ~1,420 Mb based on flow cytometry (1.45 pg/1C^27,31^). Other studies estimated the diploid plant genome size at 2,400 Mb^21,28^. Comparatively, the draft TKS genome estimates at 1,040 Mb by 19 mer, 1,140 to 1,210 Mb by flow cytometry, or the 1,290 Mb assembly (all in^4^) represent an underestimation, which signifies room for improvement in the TKS genome completeness and assembly. As *H*. *annuus* is related, but somewhat distant to TKS, we expected mis-localizations and/or ambiguities in the marker placement due to genome rearrangements and/or sequence diversity. It is noteworthy though, that many chromosome regions in the map (Fig. 1) were enriched in the markers from the same linkage groups of TKS^27^, with the gSSRs and eSSRs placed among them. This might indicate that despite a tentative character of this placement, the markers may be physically close. Thus, the markers found close on the *H*. *annuus* may indeed be linked on the TKS genome, extending the linkage information to new markers. gSSRs were slightly more ambiguously placed than eSSRs (excess of the sunflower genome BLAST hits of 2.8-fold vs. 2.2-fold, respectively), which could stem from targeting the parts of genome different in character, duplications of the non-coding regions targeted by gSSRs^36,37^, or differences in the genomes of TKS (2n = 16) and *H*. *annuus* (2n = 34).

Several studies addressed the TKS diversity at various levels; agronomic performance and rubber/inulin production was of primary concern due to the industrial potential of the plant^15,27^. Seedling growth characteristics were also studied^38^. The first attempt at estimating the species genetic diversity using molecular methods was focused on a wide collection of TKS materials and allowed for a genetic distinction of the Russian/Kazakh and Chinese TKS germplasms^33^. A milestone in the TKS molecular diversity research was the study of the Kazakhstan-originating USDA-ARS germplasm using a set of eSSRs^32^ with which we compare the statistics of our gSSR dataset. Despite our sampling scheme being lower in number than in the previous eSSRs study of TKS, our study yielded very similar results and provided significant correlation of the population distances/indices. This result was possibly accrued by employing ~50% more gSSRs at lower population sampling, yet, ensured reliability of our results. This also confirmed the general observation on the TKS diversity formulated before^32^ that the overall low species diversity resides mainly within populations. This observation is in agreement with our research hypothesis for this outcrossing, self-incompatible dandelion species. Comparison of the HWE violations in the gSSR and eSSR datasets shows much lower occurrence in the former dataset. This could be intrinsically related to the sequences targeted by either SSR type, or variable mutational frequency of the targeted loci^39,40^. This is further substantiated by the patterns of HWE violations detected. The (sub-) population violations in gSSR dataset could stem from the limited sampling, whereas locus-wise HWE violations in the eSSR dataset suggest a different underlying reason, with abundant (sub-) population TKS sampling^32^.

Developing eSSRs is generally achieved faster and easier than the gSSRs due to comparatively more conserved character of the transcriptome^39,40^. Owing to the fact of differences in parts of the genome targeted, in their conserved character, and in cross-amplification rates, both types of SSRs provide slightly different but complementary information^39,41,42^. Thus, inferences made from both types of SSRs together will provide more substantiated conclusions on the species diversity (or other studies for which they were used). Diversity of several economically important crops was analyzed using both types of SSRs, and in almost all cases led to similar results, which could also be taken as a confirmation study. For instance, deployment of both types of SSRs on the cucumber germplasm provided consistent positioning of most of the accessions analyzed on dendrograms and detected higher polymorphism rates using the gSSRs^43^. Similarly, high similarity was found between the gSSR and eSSR dendrograms among the tomato germplasms with higher polymorphism rate for the gSSRs, albeit slightly lower polymorphic information content^44^. The authors of that study postulated that combining both marker types in tomato would be effective for the species genetic diversity analyses. In contrast, studies of soybean indicated comparatively lower agreement between the gSSRs and eSSRs^45,46^. Authors argued for use of the eSSRs in soybean diversity studies for direct access to the population diversity in genes of agronomic interest but concluded that the species diversity was effectively estimated by both types of SSRs^46^. Analyses of the genetic diversity in wheat repeatedly indicated higher polymorphism of the gSSRs over eSSRs, but the authors of the studies argued that use of the eSSRs allowed for a more accurate delineation of the genetic relationships^47–49^. Studies in other cereal species observed the highest proportion of trimeric eSSRs, especially those encoding for neutral bulky amino acids^42,50^. Both studies also stated that the lower level of polymorphism detected by eSSRs compared to gSSRs might be due to the more conserved character of the targeted regions with selection acting against variation, a feature that could drive the relatively higher transferability of the eSSRs and a comparatively superior genotypic identification. Another conclusion emerged from the studies of the *Prunus* species. Although both types of SSRs resulted in similar dendrograms, combination of both datasets increased the genotypic discrimination^44^ and indicated a higher polymorphism and more effective resolution by the gSSRs than by the eSSRs^51^. The emerging conclusion from those and other studies is that similar levels of genetic diversity between populations or species may be recorded by using either SSR type with eSSRs often detecting lower variation, but performing more reliably at species differentiation^52–54^.

Cross-amplification with the TKS gSSRs proved very successful and our markers transferred to other genera of the Asteraceae (Fig. 6; Supplementary Table S1). Within the *Taraxacum* genus, the 14 gSSRs tested extensively in this study also cross-amplified to four independent gDNA samples of *T*. *brevicorniculatum* (^26^; Nowicki *et al*. unpublished data; Fig. 6). The outgroup specimens that cross-amplified with our gSSRs for TKS belonged to distant subtribes (*Taraxacum* and *Youngia* are in the subtribe *Crepidinae*; *Hypochaeris* in the *Hypochaeridinae*; *Krigia* in the *Mricroseridinae*; *Lactuca* in the *Lactucinae*; and *Pyrrhopappus* in the *Cichoriinae*), but the *Erigeron* specimens belong to a distant tribe *Asterae*. This indicates a possible broad application of our gSSRs in the Asteraceae crops analyses. The TKS eSSRs also cross-amplified with four gDNA samples of local dandelions^32^. Thus, our gSSRs present additional resources to the classical (GA/CT)_n_ gSSRs identified by restriction digest, hybridization, and Sanger sequencing^55^.

Both eSSR and gSSR datasets of TKS confirmed its sexual reproduction as observed in nature^26,32,34,56^. In contrast, results of the US dandelions are in agreement with the previous studies^25,54,57,58^ that provided evidence of both sexual and asexual modes of reproduction present in *T*. *officinale* with a broad cross-amplification to related species. The retrieved ITS sequences remained largely indiscriminate as to the species identity of the local US dandelions, co-localizing with the *T*. *officinale* ITS sequence consensus and the historical Herbarium specimen. Yet, previous research indicated predominance of only three *Taraxacum* species in North America (*T*. *ceratophorum*, *T*. *erythrospermum*, and *T*. *officinale*^25,57–59^). Including in the phylogenetic analyses the respective ITS consensus sequences of those three species, of the historical *T*. *officinale* specimen, and of *T*. *officinale* used for previous research^26^ (and data not shown) suggested the bulk of the US local dandelions could belong to *T*. *officinale*, if the microspecies of *Taraxacum* are disregarded^20,60^. Notably, the obligatory sexual diploid TKS was segregated with high confidence from the bulk of the US dandelions, as was the Central Asia-frequent *T*. *brevicorniculatum*.

The results of our gSSR analyses of this collection of US dandelions are in agreement with the recent ploidy analyses of the North America common dandelions^25^. The majority of our dataset was tri- or tetra-ploid, and it is possible that we used too few markers to capture the higher levels of ploidy of the remaining several local dandelions samples classified as diploid based on the allele counts alone. In contrast to TKS, the US *T*. *officinale* presented evidence of population structure. This is in agreement with the biology of both species, especially considering the postulated clonal reproduction of the alloploid apomictic *T*. *officinale* in North America^25,57,60^. The higher frequency of sampling the outgroup specimens belonging to distant genera in the Southeastern US may be worth investigating in regard to the species range.

Species of *Taraxacum* are notorious for hybridization, which often results in genome rearrangements, regional gDNA duplications, and/or polyploidization^21,34,57^. Cross-amplification of the TKS gSSRs (this study) and eSSRs (confirmed on four samples^32^), could help invigorate the molecular and genomic analyses of the more demanding polyploid dandelions^25,55,57^. Our study distinguishes the local US populations of *T*. *officinale* from TKS in several aspects. First, higher frequency of HWE violations indicated a difference in the US dandelions dataset. Second, the higher ploidy in this dataset inferred from the number of alleles detected indicated the possibility of clonal/asexual reproduction, which was further supported by the Index of association (I_A_). Third, several analyses indicated presence of population structure in this dataset contrary to the outcrossing diploid TKS. Overall, our gSSRs present a useful analytical tool for *Taraxacum* spp., due to cross-amplification in related species, even in distant genera.

## Conclusions and Outlook

Results on the genetic diversity of TKS accrued in the course of this study may help current and future breeding efforts of this potential crop for renewable rubber. Complementary and congruent data obtained from both gSSR and eSSR study on the same germplasm provided thorough insights into the species biology. Although the TKS well-annotated genome is still to come, the combined marker map located on the related sunflower genome may help advance future TKS studies. Furthermore, cross-amplification of our gSSRs into related species of dandelions and even other genera augments the currently available resources to analyze their biodiversity and provides a platform for their further research.

## Materials and Methods

### Plant materials

#### TKS germplasm

TKS germplasm (seeds) collected in Kazakhstan^24^ was obtained from USDA-ARS and identified in a previous study^15^ (Table 1 and Supplementary Table S1). Plants were grown from seed as described earlier^26^. Young fresh leaves of 60 individuals from 19 different populations as designated by USDA-ARS^24^ with their mapped locations of origin^32^ were used for genomic DNA (gDNA) extraction. We extracted three to five independent plant specimens per population for population diversity study (Tables 1 and S1). In addition, two TKS herbarium specimens, MONT 51683 (H.E. Morris, September 11, 1942) and KE 650 (C. Hobbs, July 02, 1949) submitted to us for destructive sampling, were used for comparison with the freshly collected samples. Plant tissue was subject to gDNA isolation using the DNeasy Plant Mini Kit (Qiagen, Germantown, MD) following the manufacturer’s protocol. The gDNA of the herbarium samples was isolated using the E.Z.N.A. Plant DNA Kit (Omega Bio-Tek, Norcross, GA) according to the manufacturer’s protocol. Isolated gDNA was evaluated for integrity by electrophoresing it in 2% agarose gels stained with ethidium bromide, and purity and concentration were assessed using Nanodrop ND-1000 UV/Vis (Fisher Scientific, Pittsburgh, PA).

#### United States plant materials and sequencing for species identification

Leaves of wild *T*. *officinale* Weber (*n* = 74) accessions from the Southeastern US and plants morphologically very similar were collected across different geographical regions (Tennessee, Georgia, Alabama, and Mississippi) and from eight distinct populations, as well as from historical herbarium specimens (Table 3 and Supplementary Table S1). Upon species identification by ITS sequencing (see below), specimens identified as not-*Taraxacum* spp. (*n* = 23) were set as a multiple outgroup. Leaf samples were collected in January and February of 2017, before the majority of the plants set bloom. No specific permissions were required for these locations/activities, as the materials are considered common weeds and regarded as neither endangered nor protected. Collected plant tissue was placed in ziplock bags containing silica gel (50 g each; Dri Splendor H&P Sales Inc., Vista, CA). gDNA was isolated from the freshly collected tissues with the DNeasy Plant Mini Kit (Qiagen, Germantown, MD) as per the manufacturer’s protocol. Samples of the historical *T*. *officinale* were provided to us by the University of Washington Herbarium (WTU, Seattle, WA, USA; *n* = 9) and Oregon State University (OSC, Corvalis, OR, USA; OSC 225005; Halse 7823; March 2010) for destructive sampling and analyses (Supplementary Table S1). Those samples’ gDNA was isolated using the E.Z.N.A. Plant DNA Kit (Omega Bio-Tek) according to the manufacturer’s protocol. Isolated gDNA was evaluated for integrity by electrophoresing it in 2% agarose gels stained with ethidium bromide, and purity and concentration were assessed using Nanodrop ND-1000 UV/Vis (Fisher Scientific, Pittsburgh, PA).

#### Genotyping of the Internal Transcribed Spacer (ITS) region and sequence analyses

The genotyping of the TKS and the US dandelions collection was completed using the primers ITS1 (Fw: 5′-TCCGTAGGTGAACCTGCGG-3′) and ITS4 (Rv: 5′-TCCTCCGCTTATTGATATGC-3′)^61^. Each PCR of 30 µl was composed of 1 × PCR buffer, 2.5 mM MgCl_2_, 0.25 mM dNTP, 10 ng gDNA, 0.5 µM of each primer, and 1 U of AmpliTaq Gold DNA Polymerase (Fisher Scientific, Waltham, MA). The optimized thermal profile used included an initial denaturation at 94 °C for 2 min, 40 cycles of 95 °C for 30 s, 60 °C for 1 min, 72 °C for 90 s, and the final extension at 72 °C for 7 min. For each PCR, 5 µl of products were electrophoresed in 2% agarose-TAE buffered gels stained with ethidium bromide to confirm the amplification, and the rest was purified with ExoSAP-IT (Thermo Fisher Scientific) according to the kit manual. Analytical sequencing was done at McLab (Molecular Cloning Laboratories, South San Francisco, CA) or University of Tennessee – Knoxville Genomics Core (UT; Knoxville, TN). Sequences were assembled using LaserGene SeqMan version 7.0.0 (DNAStar Inc., Madison, WI), manually inspected and corrected, and identified using BLAST at NCBI. The obtained sequence matrix was enriched for published TKS ITS data^34^ (Genbank: KF437406 and KF 437407) and the ITS consensus sequences of *T*. *ceratophorum* (*n = *3), *T*. *erythrospermum* (*n* = 12), and *T*. *officinale* (*n* = 53) from NCBI, respectively (Supplementary Table S2 and the references within). Sequences were then aligned using MAFFT with default settings^62,63^, truncated at the low-quality ends using Mesquite version 2.1^64^, and the uninformative characters removed using Seaview (version 4) Gblocks function with all the ‘less stringent selection’ options^65^. This sequence matrix was then submitted for phylogenetic analyses using RAxML GUI version 1.5^66^ for Maximum Likelihood using 100 runs, with thorough bootstrap of 10,000, bootstrap branch lengths activated, and General Time Reversible (GTR) substitution model^67^. Multiple outgroup was set by selecting the 23 samples identified as not *Taraxacum* spp. (Supplementary Table S1 and Supplementary File S1) collected from the Southeastern US along with *T*. *officinale*. Phylogenetic relationships among the samples were visualized using FigTree version 1.4.3^68^.

### Genome sequencing and gSSR discovery

Genomic DNA from the leaf sample E55/12 (hybrid progeny of the TKS USDA germplasm^24^; the detailed lineage is a proprietary information of ESKUSA GmbH, Parkstetten, Germany; chosen owing to abundant plant growth and thus availability of fresh leaf material) was isolated with the method described by Stein *et al*.^69^ and submitted to the UT Genomics Core for Illumina MiSeq sequencing at 275 bp, paired-end, on a v3,600 cycle flow cell. The gDNA library was prepared using the Nextera XT kit (Illumina Inc., San Diego, CA, USA) following the manufacturer’s protocol with minor modifications, that included doubled incubation times and omission of the Normalizing step.

Illumina sequencing adapters, low quality bases (mean quality <30), and short reads (<30 bases) were trimmed off with Skewer version 0.2.2^70^. Read quality control was performed using FastQC^71^. *De novo* assembly was performed with ABySS version 1.9.0^72^ with a k-mer size of 64. Sequence filtering for low complexity repeats was completed using the utility DustMasker^73^ on the resulting unitigs. gSSRs were identified using an in-house developed perl script. The minimum and maximum motif frequency definitions on the gSSRs were six to 20 bp for the di- and tri-nucleotide repeats and four to 20 bp for the tetra-nucleotide repeats. A pair of primers flanking each SSR was designed using Primer3^74^. For the primer design, the following parameters were selected: optimum primer size of 21 bp (in the range of 18 to 27); optimum annealing temperature of 60.0 °C (in the range of 55 to 65 °C); primer GC content in the range of 40 to 60%.

### SSR and marker map

The TKS genome sequence^4^ was used in combination with the TKS linkage map information^27^ to infer the genomic locations of the SSR markers in this study. We used the marker sequences published therein, those obtained from our *de novo* sequencing gSSR search, as well the marker information and/or primer sequences of the published TKS eSSRs^32^ for comparison. The marker sequences were compared to the TKS genome contigs assembly of Lin *et al*.^4^ using gmap with default scoring settings (except for –allow-close-indels = 2 and –nosplicing). For each best sequence match to the TKS genome, a ~1 kb region containing the marker (500 bp on either side) was selected. The resultant contig fragments were used to BLAST the genome of related species, sunflower *Helianthus annuus* L., HA412-HO bronze assembly^75^. Best-hit sequences were then drawn on a map, respective to their physical locations on the sunflower chromosomes. If multiple best-hits had the same e-value, all were retained.

### SSR genotyping and analyses

PCR genotyping of the collection of TKS gDNA samples was completed using a set of 25 gSSR primers identified as described above (Tables 2 and S1) with subsequent capillary electrophoresis (QIAxcel Advanced Electrophoresis System, Qiagen). The single gDNA sample E55/12 that served for *de novo* sequencing was used for an initial genotyping screen with 50 primer pairs (25 di- and 25 tri-nucleotide repeats) with the PCR procedure described below. The results were visualized by capillary electrophoresis using Qiaxcel (Qiagen) and analyzed by using 25 to 500 bp DNA size marker and internal 15/600 bp alignment marker. We screened the results of genotyping with the 50 gSSRs for specificity on this gDNA sample, and the best-performing 25 gSSRs were selected for the analysis of the TKS gDNA collection (see Supplementary Table S1 for primer sequences). Cross-amplification to the US dandelions collection (*T*. *officinale* and outgroup specimens, Supplementary Table S1) was first checked on the four random gDNA samples isolated from plants local to Knoxville, TN using the 25 best-performing gSSRs on the TKS gDNA collection. The results were then screened in a fashion similar to the TKS screening procedure.

PCR reactions of 10 µl were composed of 1 × PCR buffer, 2 mM MgCl_2_, 0.25 mM dNTP, 5% (v/v) DMSO, 4 ng gDNA, 1 µM of each primer, and 1 U of AmpliTaq Gold DNA Polymerase (Fisher Scientific). The experimentally optimized thermal profile used included an initial denaturation at 94 °C for 3 min, 15 touch-down cycles of 95 °C for 40 s, 63-0.5 °C/cycle for 40 s, 72 °C for 30 s, 25 cycles of 95 °C for 40 s, 55 °C for 40 s, 72 °C for 30 s, and the final extension at 72 °C for 4 min.

### Analysis of population structure

A total of 62 TKS gDNA samples were genotyped using 25 gSSRs and binned using FlexiBin (an MS Excel macro^76^). In addition, the published dataset of TKS-eSSR study was retrieved^32^ and binned to allow comparison of the datasets. Lastly, the dataset of *T*. *officinale* collected in the US (*n* = 74) and genotyped using 14 gSSRs was also binned, following the same procedure as the two datasets mentioned above. The binned datasets were analyzed separately for an array of population genetics parameters. To estimate the fixation and differentiation indices (F_ST_ and F’_ST_, respectively^77^), we used packages: *poppr*^78,79^, *hierfstat*^80,81^, and *polysat*^82,83^ in R version 3.4.3^84^. Due to the detected variation in ploidy levels in the US dandelions dataset, the data was corrected for ploidy in R version 3.4.3 using the package *polysat* and then recoded as tetraploid with occasionally missing alleles when samples were actually di- or tri-ploid. The mixed ploidy of that dataset limited the scope of the indices accrued, notably the differentiation index F’_ST_^77^; we resorted to GenoType/GenoDive^85^ to calculate the respective *T*. *officinale* dataset-wide F_ST_ and D’_ST_ indices. As per convention, the F_ST_ bins considered were low (F_ST_ < 0.05); moderate (0.05 < F_ST_ < 0.15), and high (F_ST_ > 0.15). Deviations of Hardy-Weinberg equilibrium (HWE) were calculated using package *pegas* version 0.10^86^ in R version 3.4.3, using the exact test based on Monte Carlo permutations of alleles (B = 1,000) and α = 0.05. The results were depicted as a probabilistic heatmap for HWE deviation in a locus- and subpopulation-manner. The multi-locus genotype (MLG) networks were constructed using the Bruvo distances, using the minimum-spanning networks (MSN) reticulation algorithm in the package *poppr* in R version 3.4.3. POPTREE2^87^ was used to calculate the population-wise distance matrices using either F_ST_ or D_ST_ indices (both standardized and unstandardized). Mantel tests were performed in R version 3.4.3 using the package MASS^88^. Analysis of the molecular variance (AMOVA) was performed in R version 3.4.3 using the package *poppr*, and the resulting Φ indices are reported as [%] values, after 1,000 permutations, at. the three levels of each dataset hierarchy: within individuals Φ_IT_, within individuals between subpopulations Φ_IS_, and among subpopulation and Φ_ST_. The mixed-ploidy *T*. *officinale* dataset did not lend itself to the Φ_IT_ calculations using AMOVA. Discriminant Analysis of Principal Components (DAPC) was performed in R version 3.4.3 using the package *adegenet* version 2.1.1^89,90^.

### Compliance with ethical standards

Research involving Human Participants and/or Animals: This article does not contain any studies with human participants or animals performed by any of the authors.

## Supplementary information

Supplementary Information

Supplementary Table S1

## Data Availability

All data generated or analyzed during this study are included in this published article and its supplementary information files. The Skewer-trimmed MiSeq reads og TKS gDNA are available at NCBI BioSample SAMN10414186, BioProject PRJNA505305.
