# Supplementary material for: Taraxacum kok-saghyz (rubber dandelion) genomic microsatellite loci reveal modest genetic diversity and cross-amplify broadly to related species
Source: Sci Rep. 2019 Feb 13;9:1915. doi: 10.1038/s41598-019-38532-8 (PMC6374447; doi:10.1038/s41598-019-38532-8)
Supplement: Supplementary file 1 — Supplementary Information [file 41598_2019_38532_MOESM1_ESM.docx]

***Taraxacum kok-saghyz* (rubber dandelion) genomic microsatellite loci reveal modest species diversity and cross-amplify broadly to related species**

Marcin Nowicki, Yichen Zhao, Sarah L Boggess, Helge Fluess, Miriam Payá-Milans, Margaret Staton, Logan C. Houston, Denita Hadziabdic, Robert N Trigiano

Supplementary Figure S1. Annual global rubber production and search for alternatives. Rubber production data were extracted from FAOSTAT (right vertical axis; dashed black line). For the alternative rubber-producing crops, top three plant species considered for natural latex production were datamined from scholar.google.com (May 2018) as Latin binomials or popular names present in the title, for the respective annual publication counts. This data is expressed as % of maximum annual publication counts (left vertical axis; *Ficus elastica*_MAX_ = 13; blue line; *Hevea brasiliensis*_MAX_ = 323; grey line; *Parthenium argentatum*_MAX_ = 99; red line; *Taraxacum kok-saghyz* [TKS] _MAX_ = 21; yellow line).


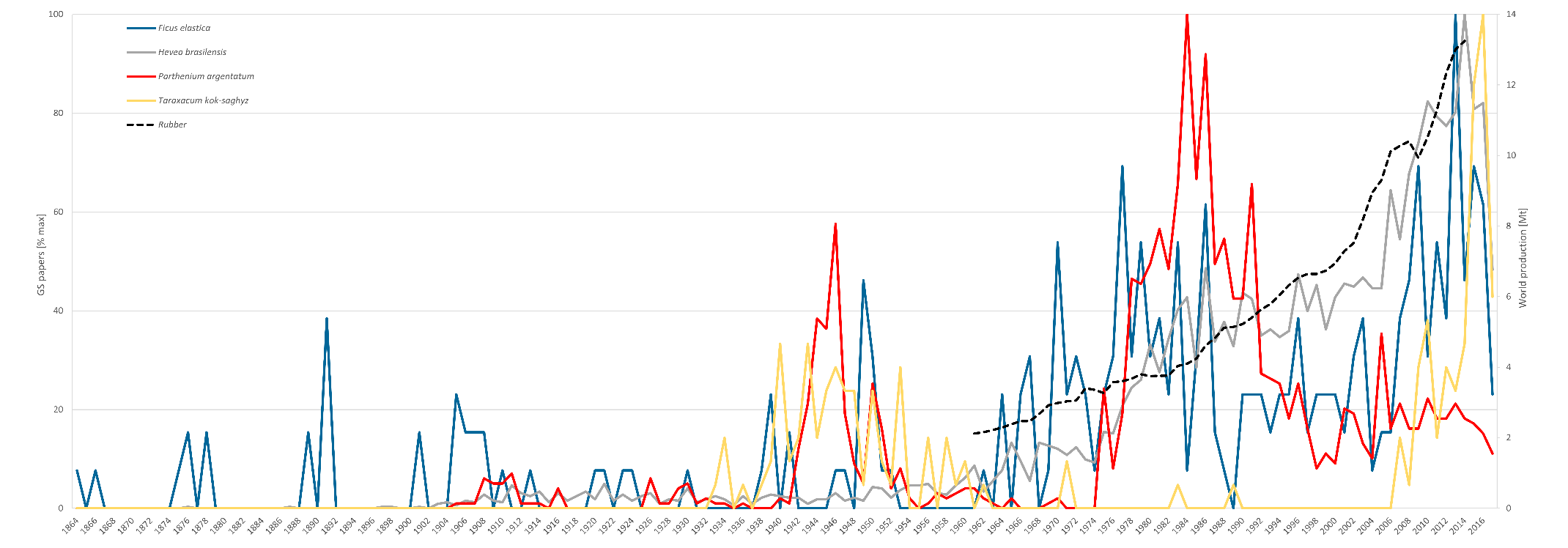


Supplementary Figure S2. Hardy-Weinberg equilibrium (HWE) for the *Taraxacum kok-saghyz* (TKS) gSSR dataset (left panel), TKS eSSR dataset of McAssey et al.^32^ (middle panel), and US *T. officinale* using 14 gSSRs selected for this study (right panel). For each dataset, the respective loci (vertical axis) and populations (horizontal axis) are indicated. Lower values (pink) violate the HWE assumptions, based on 1,000 permutations of each dataset, respectively.
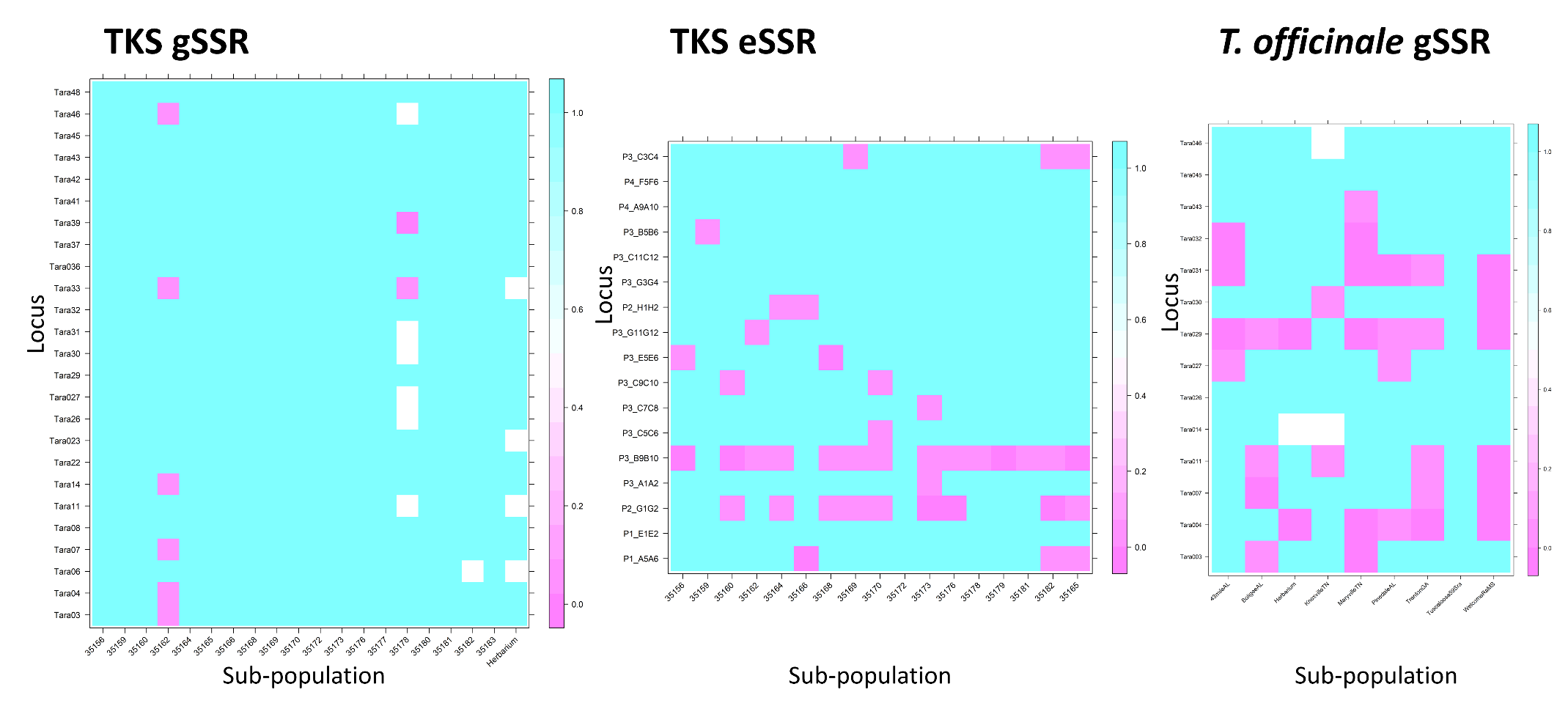


Supplementary Figure S3. Genotype accumulation curves for the *Taraxacum kok-saghyz* (TKS) gSSR dataset (left panel), TKS eSSR dataset of McAssey et al.^32^ (middle panel), and US US *T. officinale* using 14 gSSRs selected for this study (right panel). Each graph indicates number of loci necessary to saturate the MLG detected in each dataset, respectively.


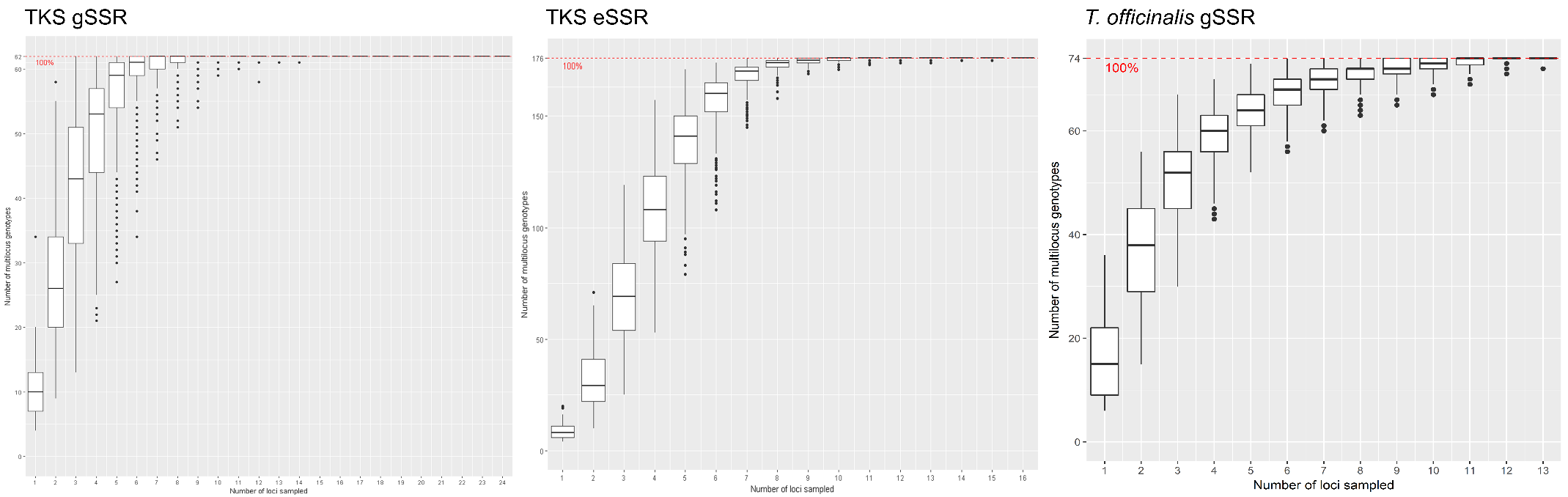


Supplementary Figure S4. Assessment of clonality by Index of Association for the *Taraxacum kok-saghyz* (TKS) gSSR dataset (left panel), TKS eSSR dataset of McAssey et al.^32^ (middle panel), and US US *T. officinale* using 14 gSSRs selected for this study (right panel). Each graph indicates the standardized overall index of association$(\bar{r}$_d_) with the corresponding statistical support (*p*), respectively.


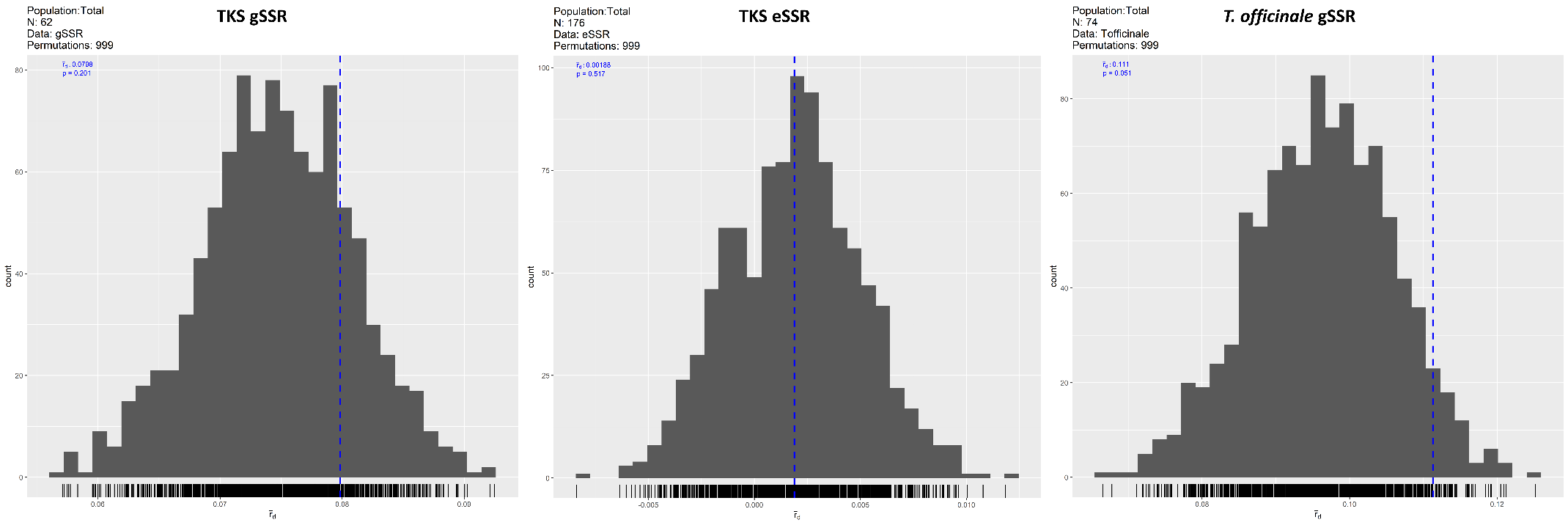


Supplementary Figure S5. Assessment of Linkage Disequilibrium by standardized overall Index of Association $(\bar{r}$_d_) for the *Taraxacum kok-saghyz* (TKS) gSSR dataset (left panel), TKS eSSR dataset of McAssey et al.^32^ (middle panel), and US *T. officinale* using 14 gSSRs selected for this study (right panel). Each graph indicates the pairwise standardized overall index of association among the loci, with the corresponding scales, respectively. Higher values (red) denote pairs of loci more likely to be inherited together (linked), and lower values (negative; blue) denote pairs of loci more likely to not be inherited together (unlinked).


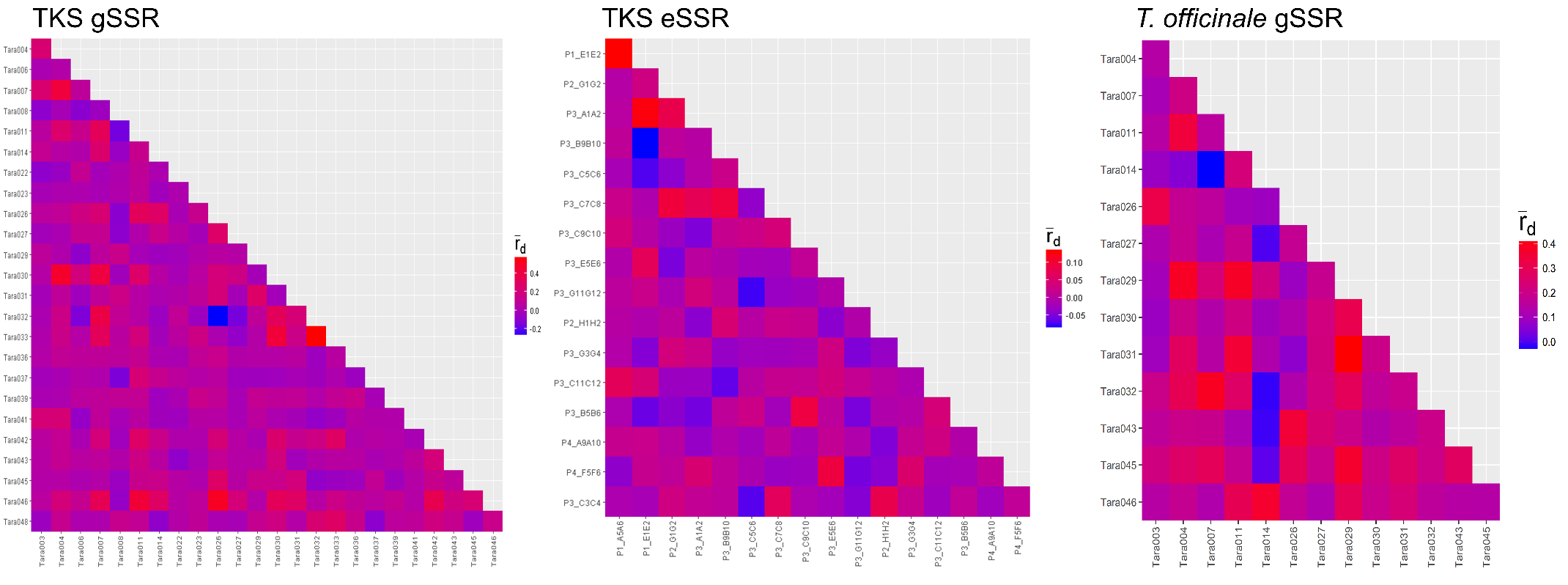


Supplementary Figure S6. Mantel comparison of population-wise genetic distance (F_ST_) matrices for *Taraxacum kok-saghyz* (TKS) datasets. The genomic SSR (gSSR) and genic SSR (eSSR) data were analyzed for their correlation, each stratified with the population as denoted by USDA-ARS for the germplasm studied. Distance matrices were calculated for populations present in both datasets.

^
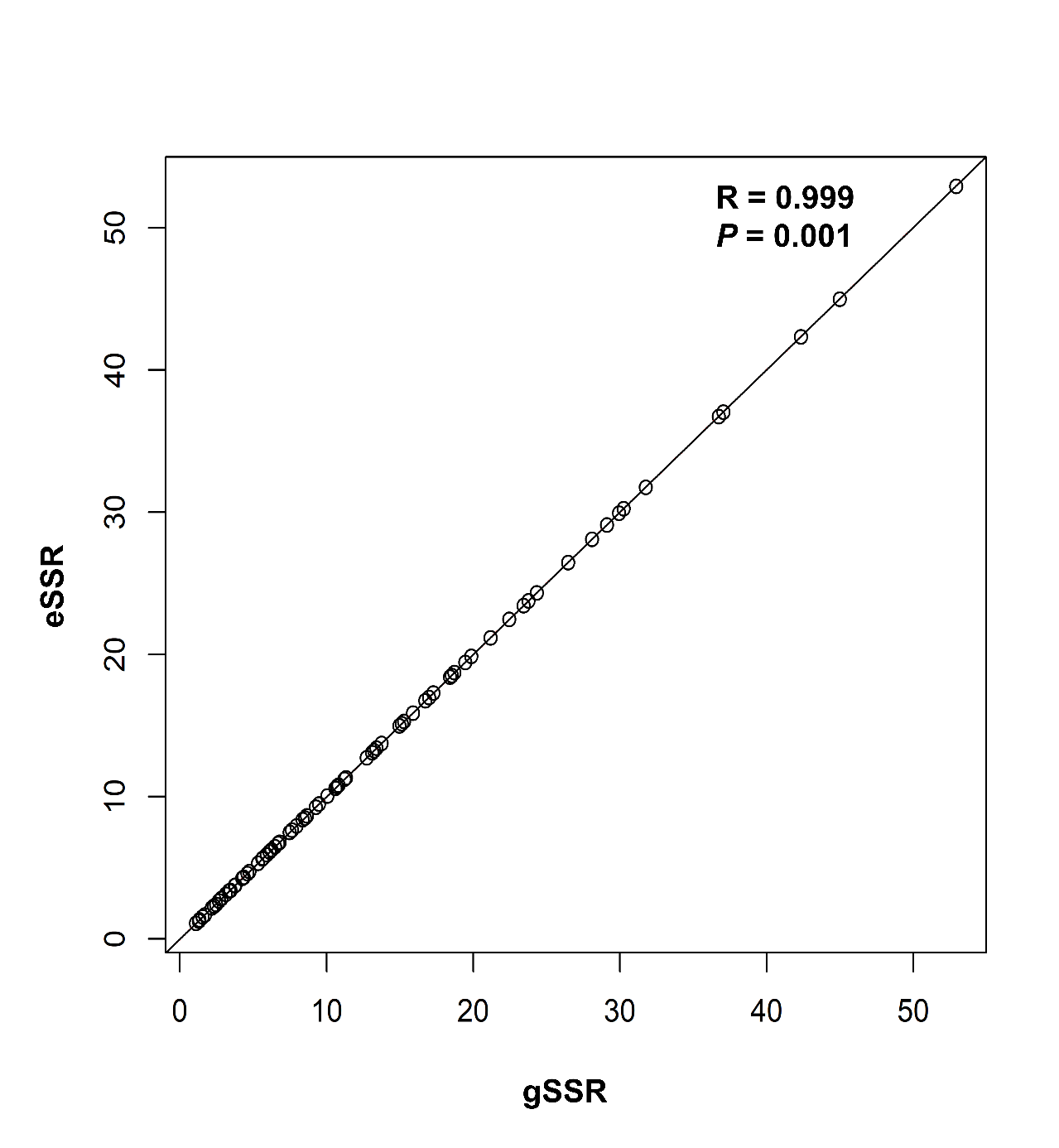
^

Supplementary Table S2. *Taraxacum* spp. ITS sequences pulled from the NCBI Genbank for the molecular species identification of the US plant materials. For each species, respectively, the Genbank accession numbers (and reference, if present) are indicated.

| **Species** | **Genbank#** | **Ref.** |
| --- | --- | --- |
| *T. ceratophorum* | JN999666_1 | ^1^ |
|  | JN999665_1 | ^1^ |
|  | JN999664_1 | ^1^ |
| *T. erythrospermum* | MG217892.1 | ^2^ |
|  | MG217610.1 | ^2^ |
|  | MG519633.1 |  |
|  | MG519632.1 |  |
|  | MG519631.1 |  |
|  | MG519630.1 |  |
|  | MG519629.1 |  |
|  | KF437457.1 |  |
|  | KF437430.1 |  |
|  | KF437429.1 |  |
|  | KF437416.1 |  |
|  | AJ633291.1 | ^3^ |
| *T. officinale* | KT249884.1 | ^4^ |
|  | KT249883.1 | ^4^ |
|  | KT249880.1 | ^4^ |
|  | JQ230979.1 |  |
|  | HQ161934.1 | ^5^ |
|  | AY862583.1 |  |
|  | AY862576.1 |  |
|  | AY548211.1 |  |
|  | MG519322.1 | ^6^ |
|  | MG519321.1 |  |
|  | MG519320.1 |  |
|  | MG519319.1 |  |
|  | MG519318.1 |  |
|  | MG519317.1 |  |
|  | MG519316.1 |  |
|  | MG519315.1 |  |
|  | MG519314.1 |  |
|  | MG519313.1 |  |
|  | MG519312.1 |  |
|  | MG519311.1 |  |
|  | MG519310.1 |  |
|  | MG519309.1 |  |
|  | MG519308.1 |  |
|  | MG519307.1 |  |
|  | MG519306.1 |  |
|  | MG519305.1 |  |
|  | MG519304.1 |  |
|  | MG519303.1 |  |
|  | MG519302.1 |  |
|  | MG519301.1 |  |
|  | MG519300.1 |  |
|  | MG519299.1 |  |
|  | MG519298.1 |  |
|  | MG519297.1 |  |
|  | MG519296.1 |  |
|  | MG519295.1 |  |
|  | MG519294.1 |  |
|  | MG519293.1 |  |
|  | MG519292.1 |  |
|  | MG519291.1 |  |
|  | MG519290.1 |  |
|  | MG519289.1 |  |
|  | MG519288.1 |  |
|  | KY860926.1 |  |
|  | KM887383.1 |  |
|  | MG219112.1 | ^2^ |
|  | MG217884.1 | ^2^ |
|  | L48338.1 T | ^7^ |
|  | L48337.1 T | ^7^ |
|  | AJ228657.1 | ^8^ |
|  | AJ228656.1 | ^8^ |
|  | AB766235.1 |  |
|  | AJ633290.1 | ^3^ |

^1^ Kuzmina, M. L., Johnson, K. L., Barron, H. R. & Hebert, P. D. Identification of the vascular plants of Churchill, Manitoba, using a DNA barcode library. *BMC ecology* **12**, 25 (2012).

^2^ Kuzmina, M. L. *et al.* Using Herbarium‐derived DNAs to assemble a large‐scale DNA barcode library for the cascular plants of Canada. *Applications in plant sciences* **5**, 1700079 (2017).

^3^ Gemeinholzer, B., Oberprieler, C. & Bachmann, K. Using GenBank data for plant identification: possibilities and limitations using the ITS 1 of Asteraceae species belonging to the tribes Lactuceae and Anthemideae. *Taxon* **55**, 173-187 (2006).

^4^ Choi, Y.-J. & Thines, M. Host jumps and radiation, not co‐divergence drives diversification of obligate pathogens. A case study in downy mildews and Asteraceae. *PLoS One* **10**, e0133655 (2015).

^5^ Schilling, E. E., Floden, A. & Schilling, D. E. Barcoding the Asteraceae of Tennessee, tribe Cichorieae. *Phytoneuron* **19**, 1-8 (2015).

^6^ Jafari, M. *et al.* Genetic diversity and biogeography of *T. officinale* inferred from multi locus sequence typing approach. *PloS one* **13**, e0203275 (2018).

^7^ Kim, S.-C., Crawford, D. J., Francisco-Ortega, J. & Santos-Guerra, A. A common origin for woody *Sonchus* and five related genera in the Macaronesian islands: molecular evidence for extensive radiation. *Proceedings of the National Academy of Sciences* **93**, 7743-7748 (1996).

^8^ Koopman, W. J., Guetta, E., van de Wiel, C. C., Vosman, B. & van den Berg, R. G. Phylogenetic relationships among *Lactuca* (Asteraceae) species and related genera based on ITS‐1 DNA sequences. *American Journal of Botany* **85**, 1517-1530 (1998).

Supplementary File S1. ITS sequences of Taraxacum spp. and outgroup (identified as non-Taraxacum spp.) retrieved in study, consensi retrieved from NCBI (Supplementary Table S2), or Kirschner et al.34 for T. kok-saghyz. The complete analytical pipeline for the sequence matrix manipulation and construction of the phylogenetic tree (Fig. 6) is described in detail in the Materials and Methods section.

>TerythrospermumNCBIconsensus

TCGAACCCTGCAAAGGCAGAACGACCTGTGAACACGTAAATACAANTGGGTGATGGGGAGATGGATCTTGGTTCTGATCCTCAACACCTCCTAGCGTGCCTGCATGCTTTCTCTTTTGGGCTATCATGCATGTATTGTTGGAATTTAACAAAACCCCGGCACGGCATGTGCCAAGGAAAACAAATAAACNAGAAGGACTCGACCTGTTATGCCCCGTTTGTGGTGTGCATTCTGAGCGTGTCCTCCTTTGAATCACAAACGACTCTCGGCAACGGATATCTCGGCTCACGCATCGATGAAGAACGTAGCAAAATGCGATACTTGGTGTGAATTGCAGAATCCCGTGAACCATCGAGTTTTTGAACGCAAGTTGCGCCCGAAGCCATCCGGTTNAGGGCACGCCTGCCTGGGCGTCACGCATCGCGTNGCCCCCCATCATACTTCCCTTAAGGGTAGTCGTGGTGATTGGGAGCGGAGATTGGCNTCCCGTGCTTGTNGTGCGGTTGGTCAAAATAGGAGTCCCCTTCGGTGGACACACGGCTAGTGGTGGTTGTGAAAGACCCTTTTCTTNTGCTGTGTGTTGTGAGCTGTNTNGGGAAACCCTCAAAAAAGACCCCAATGTAGTCGTTCTAGGATGATGCTTCGACCGCGACCCCAGGGGTCAGGCGGGGACTACCCGCTGAGTTTAAGCATATCAATAAGCGGAGGAAAAGAAACTTACAAGGATTCCCTTAGTAACGGCGAGCGAACCGGGATCAGCCCAGCTTGAAAATCGGGCGGCTTTGCTGTCCGA

>TofficinaleNCBIconsensus

TGCGAACCCTGCAAGGCAGAACGACCTGTGAACACGTAAATACAACTGGGTGATGGGGAGATGGATCTTGGTTCTGGATCCTCAACACCTCCTAGCGTGCCTGCATGCTTTCTCTTTTGGGCTATCATGCATGTATTGTTGGAATTTTAACAAAACCCCGGCACGGCATGTGCCAAGGAAAACAATAAACGAGAAGGACTCGACCTGTTATGCCCCGTTTTGTGGTGTGCATTCTGAGCGTGTCCTCCTTTGAATCACAAACGACTCTCGGCAACGGATATCTCGGCTCACGCATCGATGAAGAACGTAGCAAAATGCGATACTTGGTGTGAATTGCAGAATCCCGTGAACCATCGAGTTTTTGAACGCAAGTTGCGCCCGAAGCCATCCGGTTCAGGGCACGCCTGCCTGGGCGTCACGCATCGCGTCGCCCCCCATCATACTTCCCTTAAGGGTAGTCGTGGTGATTGGGAGCGGAGATTGGCTTCCCGTGCTTGTTGTGCGGTTGGTCAAAATAGGAGTCCCCTTCGGTGGACACACGGCTAGTGGTGGTTGTAAAGACCCTTTTCTTCTGCTGTGTGTTGTGAGCTGCTAGGGAAACCCTCAAAAAAGAACCCAATGTATCGTTCTAGGACGGATGCTTCGACC

>KF437406_TKS_Ceratoidea

ggctatcatgcatgtattgttgggcattaacaaaaccccggcacggcatgtgccaaggaaaataataaacgagaaggacacgacttgttatgccccgtttgcggtgtgcattccgagcgtgtcctcctttgaatcacaaacgactctcggcaacggatatctcggctcacgcatcgatgaagaacgtagcaaaatgcgatacttggtgtgaattgcagaatcccgtgaaccatcgagtttttgaacgcaagttgcgcctgaagccatccggttgagggcacgcctgcctgggcgtcacgcatcgcgtcgccccccatcatacttctcttaagggtagttttggtgattgggagcggagattggcctcctgtgcttgttgtgcggttggtccaaataggagtccccttcggtggacacacggctagtggtggttgtaaagacccttttcttctgttgtgtgtcgtgagctgcwaggaaaccctcaaaaaagaccccattgtattgtcctaggatgatgc

>KF437407_TKS_Ceratoidea

ggctatcatgcatgtattgttgggcattaacaaaaccccggcacggcatgtgccaaggaaaataataaacgagaaggacacgacttgttatgccccgtttgcggtgtgcattccgagcgtgtcctcctttgaatcacaaacgactctcggcaacggatatctcggctcacgcatcgatgaagaacgtagcaaaatgcgatacttggtgtgaattgcagaatcccgtgaaccatcgagtttttgaacgcaagttgcgcctgaagccatccggttgagggcacgcctgcctgggcgtcacgcatcgcgtcgccccccatcatacttctcttaagggtagttttggtgattgggagcggagattggcctcctgtgcttgttgtgcggttggtccaaataggagtccccttcggtggacacacggctagtggtggttgtaaagacccttttcttctgttgtgtgtcgtgagctgcaaggaaaccctcaaaaaagaccccattgtattgtcctaggat

>TKSherbMontana

AAGGATCATTGTCGAACCCTGCACGGCAGAACGACCTGTGAACACGTAAATACAACCGGGTGATGGGGAGATGGATCTTGGTTTTGATTCTCAACACCTCCCAACGTGCCTGCGTGCTTTCTCTTTTGGGCTATCATGCATGTATTGTTGGGCATTAACAAAACCCCGGCACGGCATGTGCCAAGGAAAATAATAAACGAGAAGGACACGACTTGTTATGCCCCGTTTGCGGTGTGCATTCCGAGCGTGTCCTCCTTtGAATCACAAACGACTCTCGGCAACGGATATCTCGGCTCACGCATCGATGAaGAACGTAGCAAAATGCGATACTTGGTGTGAATTGCAGAATCCCGTGAACCATcGAGtTTTTGAACGCAAGTTGCGCCTGAAGCCATCCGGTTGAGGGCACGCCTGCCTGGGCGTCACGCATCGCGTCGCCCCCCATCATACTTCTCTTAAGGGTAGTTTTGGTGATTGGGAGCGGAGATTGGCCTCCTGTGCTTGTTGTGCGGTTGGTCCAAATAGGAGTCCCCTTCGGTGGACACACGGCTAGTGGTGGTTGTAAAGACCCTTTTCTTCTGTTGTGTGTCGTGAGCTGCAAGGAAACCCTCAAAAAAGACCCCATTGTATTGTCCTAGGATGATGCTCGACCGCGACCCCAGGTCAGCG

>TKShebrKent

AAGGATCATTGTCGAACCCTGCACGGCAGAACGACCTGTGAACACGTAAATACAACCGGGTGATGGGGAGATGGATCTTGGTTTTGATTCTCAACACCTCCCAACGTGCCTGCGTGCTTTCTCTTTTGGGCTATCATGCATGTATTGTTGGGCATTAACAAAACCCCGGCACGGCATGTGCCAAGGAAAATAATAAACGAGAAGGACACGACTTGTTATGCCCCGTTTGCGGTGTGCATTCCGAGCGTGTCCTCCTTtGAATCACAAACGACTCTCGGCAACGGATATCTCGGCTCACGCATCGATGAAGAACGTAGCAAAATgcGataCtTGGTGtGaAttgcAGAATcccGTGAACCATCGAGTTTTTGAACGCAAGTTGCGCCTGAAGCCATCCGGTTGAGGGCACGCCTGCCTGGGCGTCACGCATCGCGTCGCCCCCCATCATACTTCTCTTAAGGGTAGTTTTGGTGATTGGGAGCGGAGATTGGCCTCCTGTGCTTGTTGTGCGGTTGGTCCAAATAGGAGTCCCCTTCGGTGGACACACGGCTAGTGGTGGTTGTAAAGACCCTTTTCTTCTGTTGTGTGTCGTGAGCTGCAAGGAAACCCTCAAAAAAGACCCCATTGTATTGTCCTAGGATGATGCTTCGACCGCGACCgCAGTCAgGCgG

>ESKUSA_E55/12

AAGGATCATTGTCGAACCCTGCACGGCAGAACGACCTGTGAACACGTAAATACAACCGGGTGATGGGGAGATGGATCTTGGTTTTGATTCTCAACACCTCCCAACGTGCCTGCGTGCTTTCTCTTTTGGGCTATCATGCATGTATTGTTGGGCATTAACAAAACCCCGGCACGGCATGTGCCAAGGAAAATAATAAACGAGAAGGACACGACTTGTTATGCCCCGTTTGCGGTGTGCATTCCGAGCGTGTCCTCCTTTGAATCACAAACGACTCTCGGCAACGGATATCTCGGCTCACGCATCGATGAAGAACGTAGCAAAATGCGATACTTGGTGTGAATTGCAGAATCCCGTGAACCATCGAGTTTTTGAACGCAAGTTGCGCCTGAAGCCATCCGGTTGAGGGCACGCCTGCCTGGGCGTCACGCATCGCGTCGCCCCCCATCATACTTCTCTTAAGGGTAGTTTTGGTGATTGGGAGCGGAGATTGGCCTCCTGTGCTTGTTGTGCGGTTGGTCCAAATAGGAGTCCCCTTCGGTGGACACACGGCTAGTGGTGGTTGTAAAGACCCTTTTCTTCTGTTGTGTGTCGTGAGCTGCAAGGAAACCCTCAAAAAAGACCCCATTGTATTGTCCTAGGATGATGCTTCGACCGCGACCCCAGGTCAGGCGGGACTACCCGCTGAGTTT

>ESKUSA_E30/12

AAGGATCATTGTCGAACCCTGCACGGCAGAACGACCTGTGAACACGTAAATACAACCGGGTGATGGGGAGATGGATCTTGGTTTTGATTCTCAACACCTCCCAACGTGCCTGCGTGCTTTCTCTTTTGGGCTATCATGCATGTATTGTTGGGCATTAACAAAACCCCGGCACGGCATGTGCCAAGGAAAATAATAAACGAGAAGGACACGACTTGTTATGCCCCGTTTGCGGTGTGCATTCCGAGCGTGTCCTCCTTTGAATCACAAACGACTCTCGGCAACGGATATCTCGGCTCACGCATCGATGAAGAACGTAGCAAAATGCGATACTTGGTGTGAATTGCAGAATCCCGTGAACCATCGAGTTTTTGAACGCAAGTTGCGCCTGAAGCCATCCGGTTGAGGGCACGCCTGCCTGGGCGTCACGCATCGCGTCGCCCCCCATCATACTTCTCTTAAGGGTAGTTTTGGTGATTGGGAGCGGAGATTGGCCTCCTGTGCTTGTTGTGCGGTTGGTCCAAATAGGAGTCCCCTTCGGTGGACACACGGCTAGTGGTGGTTGTAAAGACCCTTTTCTTCTGTTGTGTGTCGTGAGCTGCAAGGAAACCCTCAAAAAAGACCCCATTGTATTGTCCTAGGATGATGCTTCGACCGCGACCCCAGGTCAGGCGGGACTACCCGCTGAGTTTAAGCATATCAATAAGGCGGAGGAA

>TKSregenerantS17

AAGGATCATTGTCGAACCCTGCACGGCAGAACGACCTGTGAACACGTAAATACAACCGGGTGATGGGGAGATGGATCTTGGTTTTGATTCTCAACACCTCCCAACGTGCCTGCGTGCTTTCTCTTTTGGGCTATCATGCATGTATTGTTGGGCATTAACAAAACCCCGGCACGGCATGTGCCAAGGAAAATAATAAACGAGAAGGACACGACTTGTTATGCCCCGTTTGCGGTGTGCATTCCGAGCGTGTCCTCCTTTGAATCACAAACGACTCTCGGCAACGGATATCTCGGCTCACGCATCGATGAAGAACGTAGCAAAATGCGATACTTGGTGTGAATTGCAGAATCCCGTGAACCATCGAGTTTTTGAACGCAAGTTGCGCCTGAAGCCATCCGGTTGAGGGCACGCCTGCCTGGGCGTCACGCATCGCGTCGCCCCCCATCATACTTCTCTTAAGGGTAGTTTTGGTGATTGGGAGCGGAGATTGGCCTCCTGTGCTTGTTGTGCGGTTGGTCCAAATAGGAGTCCCCTTCGGTGGACACACGGCTAGTGGTGGTTGTAAAGACCCTTTTCTTCTGTTGTGTGTCGTGAGCTGCAAGGAAACCCTCAAAAAAGACCCCATTGTATTGTCCTAGGATGATGCTTCGACCGCGACCCCAGGTCAGGCGGGACTACCCGCTGAGTTTAA

>TKS35170_37

AAGGATCATTGTCGAACCCTGCACGGCAGAACGACCTGTGAACACGTAAATACAACCGGGTGATGGGGAGATGGATCTTGGTTTTGATTCTCAACACCTCCCAACGTGCCTGCGTGCTTTCTCTTTTGGGCTATCATGCATGTATTGTTGGGCATTAACAAAACCCCGGCACGGCATGTGCCAAGGAAAATAATAAACGAGAAGGACACGACTTGTTATGCCCCGTTTGCGGTGTGCATTCCGAGCGTGTCCTCCTTTGAATCACAAACGACTCTCGGCAACGGATATCTCGGCTCACGCATCGATGAAGAACGTAGCAAAATGCGATACTTGGTGTGAATTGCAGAATCCCGTGAACCATCGAGTTTTTGAACGCAAGTTGCGCCTGAAGCCATCCGGTTGAGGGCACGCCTGCCTGGGCGTCACGCATCGCGTCGCCCCCCATCATACTTCTCTTAAGGGTAGTTTTGGTGATTGGGAGCGGAGATTGGCCTCCTGTGCTTGTTGTGCGGTTGGTCCAAATAGGAGTCCCCTTCGGTGGACACACGGCTAGTGGTGGTTGTAAAGACCCTTTTCTTCTGTTGTGTGTCGTGAGCTGCAAGGAAACCCTCAAAAAAGACCCCATTGTATTGTCCTAGGATGATGCTTCGACCNGCGACCCCAGGTCAGGCGGGACTACCCGCTGAGTTTAAGCATATCAATAAAGCGGAGGAA

>TKS35172_7

AAGGATCATTGTCGAACCCTGCACGGCAGAACGACCTGTGAACACGTAAATACAACCGGGTGATGGGGAGATGGATCTTGGTTTTGATTCTCAACACCTCCCAACGTGCCTGCGTGCTTTCTCTTTTGGGCTATCATGCATGTATTGTTGGGCATTAACAAAACCCCGGCACGGCATGTGCCAAGGAAAATAATAAACGAGAAGGACACGACTTGTTATGCCCCGTTTGCGGTGTGCATTCCGAGCGTGTCCTCCTTTGAATCACAAACGACTCTCGGCAACGGATATCTCGGCTCACGCATCGATGAAGAACGTAGCAAAATGCGATACTTGGTGTGAATTGCAGAATCCCGTGAACCATCGAGTTTTTGAACGCAAGTTGCGCCTGAAGCCATCCGGTTGAGGGCACGCCTGCCTGGGCGTCACGCATCGCGTCGCCCCCCATCATACTTCTCTTAAGGGTAGTTTTGGTGATTGGGAGCGGAGATTGGCCTCCTGTGCTTGTTGTGCGGTTGGTCCAAATAGGAGTCCCCTTCGGTGGACACACGGCTAGTGGTGGTTGTAAAGACCCTTTTCTTCTGTTGTGTGTCGTGAGCTGCAAGGAAACCCTCAAAAAAGACCCCATTGTATTGTCCTAGGATGATGCTTCCACCGCGACCCCAGGTCAGGCGGGACTACCCGCTGAGTTTAAGCNTATCAATAAGCGGAGGAA

>KnoxvilleTN52

ACcTGCAgGCAGAaCGACCTGTGAaCACGTAAATACAATTGGGTGATGGGGAGATGGATCTTGGTTCTGATCCTCAACACCTCCTAGCGTGCCTGCATGCTTTCTCTTTTGGGCTATCATGCATGTATTGTTGGAATTTAACAAAACCCCGGCACGGCATGTGCCAAGGAAAACAATAAACTAGAAGGACTCGACCTGTTATGCCCCGTTTGTGGTGTGCATTCTGAGCGTGTCCTCCTTTGAATCACAAACGACTCTCGGCAACGGATATCTCGGCTCACGCATCGATGAAGAACGTAGCAAAATGCGATACTTGGTGTGAATTGCAGAATCCCGTGAACCATCGAGTTTTTGAACGCAAGTTGCGCCCGAAGCCATCCGGTTCAGGGCACGCCTGCCTGGGCGTCACGCATCGCGTCGCCCCCCATCATACTTCCCTTAAGGGTAGTCGTGGCGATTGGGAGCGGAGATTGGCTTCCCGTGCTTGTTGTGCGGTTGGTCAAAATAGGAGTCCCCTTCGGTGGACACACGGCTAgTGGTGGTTGTAAAGACCCTTTTCtTCTGCTGTGTGTTGTGAGCTGCTAGGGAAACCCTCAAAAAAGAACCCAATGTATCGTTCTAGGACGATGCTTCGACCGCGACCCCAGgTCAGGCgGA

>KnoxvilleTN53

ACcTGCAgGCAGAaCGACCTGTGAaCACGTAAATACAAyTGGGTGATGGGGAGATGGATCTTGGTTCTGATCCTCAACACCTCCTAGCGTGCCTGCATGCTTTCTCTTTTGGGCTATCATGCATGTATTGTTGGAATTTAACAAAACCCCGGCACGGCATGTGCCAAGGAAAACAATAAACGAGAAGGACTTGACCTGTTATGCCCCGTTTGTGGTGTGCATTCTGAGaCGTGTCCTCCTTTGAATCACAAACGACTCTCGGCAACGGATATCTCGGCTCACGCATCGATGAAGAACGTAGCAAAATGCGATACTTGGTGTGAATTGCAGAATCCCGTGAACCATCGAGTTTTTGAACGCAAGTTGCGCCCGAAGCCATCCGGTTCAGGGCACGCCTGCCTGGGCGTCACGCATCGCGTCGCCCCCCATCATACTTCCCTTAAGGGTAGTCGTGGTGATTGGGAGCGGAGATTGGCTTCCCGTGCTTGTTGTGCGGTTGGTCAAAATAGGAGTCCCCTTYGGTGGACACACGGCTAGTGGTGGTTGTAAAGACCCTTTTCTTCTGCTGTGTGTTGTGAGCTGCTAGGGAAACCCTCAAAAAAGAACCCAATGTATCGTTCTAGGACGATGCTTCGACCGCGACCCCAGgTCAGGCgGA

>KnoxvilleTN55

AAGGATCATTGTCGAACCCTGCaAGGCAGAACGACCTGTGAACACGTAAATACAACTGGGTGATGGGGAGATGGATCTTGGTTCTGATCCTCAACACCTCCTAGCGTGCCTGCATGCTTTCTCTTTTGGGCTATCATGCATGTATTGTTGGAATTTAACAAAACCCCGGCACGGCATGTGCCAAGGAAAACAATAAACTAGAAGGACTCGACCTGTTATGCCCCGTTTGTGGTGTGCATTCTGAGCGTGTCCTCCTTTGAATCACAAACGACTCTCGGCAACGGATATCTCGGCTCACGCATCGATGAAGAACGTAGCAAAATGCGATACTTGGTGTGAATTGCAGAATCCCGTGAACCATCGAGTTTTTGAACGCAAGTTGCGCCCGAAGCCATCCGGTTCAGGGCACGCCTGCCTGGGCGTCACGCATCGCGTCGCCCCCCATCATACTTCCCTTAAGGGTAGTCGTGGYGATTGGGAGCGGAGATTGGCTTCCCGTGCTTGTTGTGCGGTTGGTCAAAATAGGAGTCCCCTTCGGTGGACACACGGCTAGTGGTGGTTGTAAAGACCCTTTTCTTCTGCTGTGTGTTGTGAGCTGCTAGGGAAACCCTCAAAAAAGAACCCAATGTATCGTTCTAGGACGATGCTTCGACCGCGACCCCAGgTCAGGCgGA

>KnoxvilleTN52

AAGGATCATTGTCGAACCCTGCAAGGCAGAACGACCTGTGAACACGTAAATACAACTGGGTGATGGGGAGATGGATCTTGGTTCTGATCCTCAACACCTCCTAGCGTGCCTGCATGCTTTCTCTTTTGGGCTATCATGCATGTATTGTTGGAATTTAACAAAACCCCGGCACGGCATGTGCCAAGGAAAACAATAAACGAGAAGGACTCGACCTGTTATGCCCCGTTTGTGGTGTGCATTCTGAGCGTGTCCTCCTTTGAATCACAAACGACTCTCGGCAACGGATATCTCGGCTCACGCATCGATGAAGAACGTAGCAAAATGCGATACTTGGTGTGAATTGCAGAATCCCGTGAACCATCGAGTTTTTGAACGCAAGTTGCGCCCGAAGCCATCCGGTTTAGGGCACGCCTGCCTGGGCGTCACGCATCGCGTCGCCCCCCATCATACTTCCCTTAAGGGTAGTCGTGGTGATTGGGAGCGGAGATTGGCTTCCCGTGCTTGTTGTGCGGTTGGTCAAAATAGGAGTCCCCTTCGGTGGACACACGGCTAGTGGTGGTTGTAAAGACCCTTTTCTTCTGCTGTGTGTTGTGAGCTGCTAGGGAAACCCTCAAAAAAGAACCCAATGTATCGTTCTAGGACGATGCTTCGACCGCGACCCCAGgTCAGGCgGA

>TofficinaleS2

TTCCGTAGGGTGAACCTGCGGAAGGATCATTGTCGAACCCTGCAAGGCAGAACGACCTGTGAACACGTAAATACAACTGGGTGATGGGGAGATGGATCTTGGTTCTGATCCTCAACACCTCCTAGCGTGCCTGCATGCTTTCTCTTTTGGGCTATCATGCATGTATTGTTGGAATTTAACAAAACCCCGGCACGGCATGTGCCAAGGAAAACAATAAACGAGAAGGACTCGACCTGTTATGCCCCGTTTGTGGTGTGCATTCTGAGCGTGTCCTCCTTTGAATCACAAACGACTCTCGGCAACGGATATCTCGGCTCACGCATCGATGAAGAACGTAGCAAAATGCGATACTTGGTGTGAATTGCAGAATCCCGTGAACCATCGAGTTTTTGAACGCAAGTTGCGCCCGAAGCCATCCGGTTTAGGGCACGCCTGCCTGGGCGTCACGCATCGCGTCGCCCCCCATCATACTTCCCTTAAGGGTAGTCGTGGTGATTGGGAGCGGAGATTGGCTTCCCGTGCTTGTTGTGCGGTTGGTCAAAATAGGAGTCCCCTTCGGTGGACACACGGCTAGTGGTGGTTGTAAAGACCCTTTTCTTCTGCTGTGTGTTGTGAGCTGCTAGGGAAACCCTCAAAAAAGAACCCAATGTATCGTTCTAGGACGATGCTTCGACCGCGACCCCAGGTCAGGCGGA

>KnoxvilleTN54

AAGGATCATTGTCGAACCCTGCAAGGCAGAACGACCTGTGAACACGTAAATACAATTGGGTGATGGGGAGATGGATCTTGGTTCTGATCCTCAACACCTCCTAGCGTGCCTGCATGCTTTCTCTTTTGGGCTATCATGCATGTATTGTTGGAATTTAACAAAACCCCGGCACGGCATGTGCCAAGGAAAACAATAAACGAGAAGGACTCGACCTGTTATGCCCCGTTTGTGGTGTGCATTCTGAGCGTGTCCTCCTTTGAATCACAAACGACTCTCGGCAACGGATATCTCGGCTCACGCATCGATGAAGAACGTAGCAAAATGCGATACTTGGTGTGAATTGCAGAATCCCGTGAACCATCGAGTTTTTGAACGCAAGTTGCGCCCGAAGCCATCCGGTTCAGGGCACGCCTGCCTGGGCGTCACGCATCGCGTCGCCCCCCATCATACTTCCCTTAAGGGTAGTCGTGGCGATTGGGAGCGGAGATTGGCTTCCCGTGCTTGTTGTGCGGTTGGTCAAAATAGGAGTCCCCTTCGGTGGACACACGGCTAGTGGTGGTTGTAAAGACCCTTTTCTTCTGCTGTGTGTTGTGAGCTGCTAGGGAAACCCTCAAAAAAGAACCCAATGTATCGTTCTAGGACGATGCTTCGACCGCGACCCCAGGTCAGGCGGGACTACCCGCTGAGTTTAAGCATATCAATAAGCGGAGGAA

>PinedaleALM11

AAGGATCATTGTCGAACCCTGCAAGGCAGAACGACCTGTGAACACGTAAATACAACTGGGTGATGGGGAGATGGATCTTGGTTCTGATCCTCAACACCTTCTAGCGTGCCTGCATGCTTTCTCTTTTGGGCTATCATGCATGTATTGTTGGAATTTAACAAAACCCCGGCACGGCATGTGCCAAGGAAAACAATAAACGAGAAGGACTCGACCTGTTATGCCCCGTTTGTGGTGTGCATTCTGAGCGTGTCCTCCTTTGAATCACAAACGACTCTCGGCAACGGATATCTCGGCTCACGCATCGATGAAGAACGTAGCAAAATGCGATACTTGGTGTGAATTGCAGAATCCCGTGAACCATCGAGTTTTTGAACGCAAGTTGCGCCCGAAGCCATCCGGTTCAGGGCACGCCTGCCTGGGCGTCACGCATCGCGTCGCCCCCCATCATACTTCCCTTAAGGGTAGTCGTGGTGATTGGGAGCGGAGATTGGCTTCCCGTGCTTGTTGTGCGGTTGGTCAAAATAGGAGTCCCCTTCGGTGGACACACGGCTAGTGGTGGTTGTAAAGACCCTTTTCTTCTGCTGTGTGTTGTGAGCTGCTAGGGAAACCCTCAAAAAAGAACCCAATGTATCGTTCTAGGACGATGCTTCGACCGCGACCCCAGGTCAGGCGGGACTACCCGCTGAGTTTAAGCATATCAATAAGCGGAGGAA

>PinedaleALM19

AAGGATCATTGTCGAACCCTGCAAGGCAGAACGACCTGTGAACACGTAAATACAACTGGGTGATGGGGAGATGGATCTTGGTTCTGATCCTCAACACCTCCTAGCGTGCCTGCATGCTTTCTCTTTTGGGCTATCATGCATGTATTGTTGGAATTTAACAAAACCCCGGCACGGCATGTGCCAAGGAAAACAATAAACGAGAAGGACTCGACCTGTTATGCCCCGTTTGTGGTGTGCATTCTGAGCGTGTCCTCCTTTGAATCACAAACGACTCTCGGCAACGGATATCTCGGCTCACGCATCGATGAAGAACGTAGCAAAATGCGATACTTGGTGTGAATTGCAGAATCCCGTGAACCATCGAGTTTTTGAACGCAAGTTGCGCCCGAAGCCATCCGGTTCAGGGCACGCCTGCCTGGGCGTCACGCATCGCGTCGCCCCCCATCATACTTCCCTTAAGGGTAGTCGTGGTGATTGGGAGCGGAGATTGGCTTCCCGTGCTTGTTGTGCGGTTGGTCAAAATAGGAGTCCCCTTCGGTGGACACACGGCTAGTGGTGGTTGTAAAGACCCTTTTCTTCTGCTGTGTGTTGTGAGCTGCTAGGGAAACCCTCAAAAAAGAACCCAATGTATCGTTCTAGGACGATGCTTCGACCGCGACCCCAGGTCAGGCGGGACTACCCGCTGAGTTTAAGCATATCAATAAGCGGAGGAA

>MaryvilleTNT4

AAGGATCATTGTCGAACCCTGCAAGGCAGAACGACCTGTGAACACGTAAATACAACTGGGTGATGGGGAGATGGATCTTGGTTCTGATCCTCAACACCTCCTAGCGTGCCTGCATGCTTTCTCTTTTGGGCTATCATGCATGTATTGTTGGAATTTAACAAAACCCCGGCACGGCATGTGCCAAGGAAAACAATAAACGAGAAGGACTCGACCTGTTATGCCCCGTTTGTGGTGTGCATTCTGAGCGTGTCCTCCTTTGAATCACAAACGACTCTCGGCAACGGATATCTCGGCTCACGCATCGATGAAGAACGTAGCAAAATGCGATACTTGGTGTGAATTGCAGAATCCCGTGAACCATCGAGTTTTTGAACGCAAGTTGCGCCCGAAGCCATCCGGTTCAGGGCACGCCTGCCTGGGCGTCACGCATCGCGTCGCCCCCCATCATACTTCCCTTAAGGGTAGTCGTGGTGATTGGGAGCGGAGATTGGCTTCCCGTGCTTGTTGTGCGGTTGGTCAAAATAGGAGTCCCCTTCGGTGGACACACGGCTAGTGGTGGTTGTAAAGACCCTTTTCTTCTGCTGTGTGTTGTGAGCTGCTAGGGAAACCCTCAAAAAAGAACCCAATGTATCGTTCTAGGACGATGCTTCGACCGCGACCCCAGGTCAGGCGGGACTACCCGCTGAGTTTTAAGCATATCNAATAAGCGGGAGGAA

>TrentonGAM8

AAGGATCATTGTCGAACCCTGCAAGGCAGAACGACCTGTGAACACGTAAATACAACTGGGTGATGGGGAGATGGATCTTGGTTCTGATCCTCAACACCTCCTAGCGTGCCTGCATGCTTTCTCTTTTGGGCTATCATGCATGTATTGTTGGAATTTAACAAAACCCCGGCACGGCATGTGCCAAGGAAAACAATAAACGAGAAGGACTCGACCTGTTATGCCCCGTTTGTGGTGTGCATTCTGAGCGTGTCCTCCTTTGAATCACAAACGACTCTCGGCAACGGATATCTCGGCTCACGCATCGATGAAGAACGTAGCAAAATGCGATACTTGGTGTGAATTGCAGAATCCCGTGAACCATCGAGTTTTTGAACGCAAGTTGCGCCCGAAGCCATCCGGTTCAGGGCACGCCTGCCTGGGCGTCACGCATCGCGTCGCCCCCCATCATACTTCCCTTAAGGGTAGTCGTGGTGATTGGGAGCGGAGATTGGCTTCCCGTGCTTGTTGTGCGGTTGGTCAAAATAGGAGTCCCCTTCGGTGGACACACGGCTAGTGGTGGTTGTAAAGACCCTTTTCTTCTGCTGTGTGTTGTGAGCTGCTAGGGAAACCCTCAAAAAAGAACCCAATGTATCGTTCTAGGACGATGCTTCGACCGCG

>43mileALM23

AAGGATCATTGTCGAACCCTGCAAGGCAGaACGACCTGTGAACACGTAAATACAATTGGGTGATGGGGAGATGGATCTTGGTTCTGATCCTCAACACCTCCTAGCGTGCCTGCATGCTTTCTCTTTTGGGCTATCATGCATGTATTGTTGGAATTTAACAAAACCCCGGCACGGCATGTGCCAAGGAAAACAATAAACTAGAAGGACTCGACCTGTTATGCCCCGTTTGTGGTGTGCATTCTGAGCGTGTCCTCCTTTGAATCACAAACGACTCTCGGCAACGGATATCTCGGCTCACGCATCGATGAAGAACGTAGCAAAATGCGATACTTGGTGTGAATTGCAGAATCCCGTGAACCATCGAGTTTTTGAACGCAAGTTGCGCCCGAAGCCATCCGGTTCAGGGCACGCCTGCCTGGGCGTCACGCATCGCGTCGCCCCCCATCATACTTCCCTTAAGGGTAGTCGTGGCGATTGGGAGCGGAGATTGGCTTCCCGTGCTTGTTGTGCGGTTGGTCAAAATAGGAGTCCCCTTCGGTGGACACACGGCTAGTGGTGGTTGTAAAGACCCTTTTCTTCTGCTGTGTGTTGTGAGCTGCTAGGGAAACCCTCAAAAAAGAACCCAATGTATCGTTCTAGGACGATGCTTCGACCGCGACCCCAGGTCAGGCGGGACTACCCGCTGAGTTTAA

>43mileALM24

AAGGATCATTGTCGAACCCTGCAAGGCAGAACGACCTGTgAACACGTAAATACAATTGGGTGATGGGGAGATGGATCTTGGTTCTGATCCTCAACACCTCCTAGCGTGCCTGCATGCTTTCTCTTTTGGGCTATCATGCATGTATTGTTGGAATTTAACAAAACCCCGGCACGGCATGTGCCAAGGAAAACAATAAACTAGAAGGACTCGACCTGTTATGCCCCGTTTGTGGTGTGCATTCTGAGCGTGTCCTCCTTTGAATCACAAACGACTCTCGGCAACGGATATCTCGGCTCACGCATCGATGAAGAACGTAGCAAAATGCGATACTTGGTGTGAATTGCAGAATCCCGTGAACCATCGAGTTTTTGAACGCAAGTTGCGCCCGAAGCCATCCGGTTCAGGGCACGCCTGCCTGGGCGTCACGCATCGCGTCGCCCCCCATCATACTTCCCTTAAGGGTAGTCGTGGCGATTGGGAGCGGAGATTGGCTTCCCGTGCTTGTTGTGCGGTTGGTCAAAATAGGAGTCCCCTTCGGTGGACACACGGCTAGTGGTGGTTGTAAAGACCCTTTTCTTCTGCTGTGTGTTGTGAGCTGCTAGGGAAACCCTCAAAAAAGAACCCAATGTATCGTTCTAGGACGATGCTTCGACCGCGACCCCAGGTCAGGCGGGACTACCCGCTGAGTTTAA

>43mileALM63

AAGGATCATTGTCGAACCCTGCAAGGCAGAACGACCTGTGAACACGTAAATACAATTGGGTGATGGGGAGATGGATCTTGGTTCTGATCCTCAACACCTCCTAGCGTGCCTGCATGCTTTCTCTTTTGGGCTATCATGCATGTATTGTTGGAATTTAACAAAACCCCGGCACGGCATGTGCCAAGGAAAACAATAAACTAGAAGGACTCGACCTGTTATGCCCCGTTTGTGGTGTGCATTCTGAGCGTGTCCTCCTTTGAATCACAAACGACTCTCGGCAACGGATATCTCGGCTCACGCATCGATGAAGAACGTAGCAAAATGCGATACTTGGTGTGAATTGCAGAATCCCGTGAACCATCGAGTTTTTGAACGCAAGTTGCGCCCGAAGCCATCCGGTTCAGGGCACGCCTGCCTGGGCGTCACGCATCGCGTCGCCCCCCATCATACTTCCCTTAAGGGTAGTCGTGGCGATTGGGAGCGGAGATTGGCTTCCCGTGCTTGTTGTGCGGTTGGTCAAAATAGGAGTCCCCTTCGGTGGACACACGGCTAGTGGTGGTTGTAAAGACCCTTTTCTTCTGCTGTGTGTTGTGAGCTGCTAGGGAAACCCTCAAAAAAGAACCCAATGTATCGTTCTAGGACGATGCTTCGACCGCGACCCCAGGTCAGGCGGGACTACCCGCTGAGTTTAA

>43mileALM64

AAGGATCATTGTCGAACCCTGCAAGGCAGAACGACCTGTGaACACGTAAATACAATTGGGTGATGGGGAGATGGATCTTGGTTCTGATCCTCAACACCTCCTAGCGTGCCTGCATGCTTTCTCTTTTGGGCTATCATGCATGTATTGTTGGAATTTAACAAAACCCCGGCACGGCATGTGCCAAGGAAAACAATAAACTAGAAGGACTCGACCTGTTATGCCCCGTTTGTGGTGTGCATTCTGAGCGTGTCCTCCTTTGAATCACAAACGACTCTCGGCAACGGATATCTCGGCTCACGCATCGATGAAGAACGTAGCAAAATGCGATACTTGGTGTGAATTGCAGAATCCCGTGAACCATCGAGTTTTTGAACGCAAGTTGCGCCCGAAGCCATCCGGTTCAGGGCACGCCTGCCTGGGCGTCACGCATCGCGTCGCCCCCCATCATACTTCCCTTAAGGGTAGTCGTGGCGATTGGGAGCGGAGATTGGCTTCCCGTGCTTGTTGTGCGGTTGGTCAAAATAGGAGTCCCCTTCGGTGGACACACGGCTAGTGGTGGTTGTAAAGACCCTTTTCTTCTGCTGTGTGTTGTGAGCTGCTAGGGAAACCCTCAAAAAAGAACCCAATGTATCGTTCTAGGACGATGCTTCGACCGCGACCCCAGGTCAGGCGGGACTACCCGCTGAGTTTAA

>43mileALM40

AAGGATCATTGTCGAACCCTGCAAGGCAGAACGACCTGTGaACACGTAAATACAATTGGGTGATGGGGAGATGGATCTTGGTTCTGATCCTCAACACCTCCTAGCGTGCCTGCATGCTTTCTCTTTTGGGCTATCATGCATGTATTGTTGGAATTTAACAAAACCCCGGCACGGCATGTGCCAAGGAAAACAATAAACTAGAAGGACTCGACCTGTTATGCCCCGTTTGTGGTGTGCATTCTGAGCGTGTCCTCCTTTGAATCACAAACGACTCTCGGCAACGGATATCTCGGCTCACGCATCGATGAAGAACGTAGCAAAATGCGATACTTGGTGTGAATTGCAGAATCCCGTGAACCATCGAGTTTTTGAACGCAAGTTGCGCCCGAAGCCATCCGGTTCAGGGCACGCCTGCCTGGGCGTCACGCATCGCGTCGCCCCCCATCATACTTCCCTTAAGGGTAGTCGTGGYGATTGGGAGCGGAGATTGGCTTCCCGTGCTTGTTGTGCGGTTGGTCAAAATAGGAGTCCCCTTCGGTGGACACACGGCTAGTGGTGGTTGTAAAGACCCTTTTCTTCTGCTGTGTGTTGTGAGCTGCTAGGGAAACCCTCAAAAAAGAACCCAATGTATCGTTCTAGGACGATGCTTCGACCGCGACCCCAGGTCAGGCGGGACTACCCGCTGAGTTTAA

>BoligeeALM32

AAGGATCATTGTCGAACCCTGCAAGGCAGaACGACCTGTGAACACGTAAATACAACTGGGTGATGGGGAGATGGATCTTGGTTCTGATCCTCAACGCCTCCTAGCGTGCCTGCATGCTTTCTCTTTTGGGCTATCATGCATGTATTGTTGGAATTTAACAAAACCCCGGCACGGCATGTGCCAAGGAAAACAATAAACGAGAAGGACTCGACCTGTTATGCCCCGTTTGTGGTGTGCATTCTGAGCGTGTCCTCCTTTGAATCACAAACGACTCTCGGCAACGGATATCTCGGCTCACGCATCGATGAAGAACGTAGCAAAATGCGATACTTGGTGTGAATTGCAGAATCCCGTGAACCATCGAGTTTTTGAACGCAAGTTGCGCCCGAAGCCATCCGGTTCAGGGCACGCCTGCCTGGGCGTCACGCATCGCGTCGCCCCCCATCATACTTCCCTTAAGGGTAGTCGTGGTGATTGGGAGCGGAGATTGGCTTCCCGTGCTTGTTGTGCGGTTGGTCAAAATAGGAGTCCCCTTCGGTGGACACACGGCTAGTGGTGGTTGTAAAGACCCTTTTCTTCTGCTGTGTGTTGTGAGCTGCTAGGGAAACCCTCAAAAAAGAACCCAATGTATCGTTCTAGGACGATGCTTCGACCGCGACCCCAGGTCAGGCGGGACTACCCGCTGAGTTTAA

>BoligeeALM38

AAGGATCATTGTCGAACCCTGCAAGGCAGAACGACCTGTGaACACGTAAATACAACTGGGTGATGGGGAGATGGATCTTGGTTCTGATCCTCAACGCCTCCTAGCGTGCCTGCATGCTTTCTCTTTTGGGCTATCATGCATGTATTGTTGGAATTTAACAAAACCCCGGCACGGCATGTGCCAAGGAAAACAATAAACGAGAAGGACTCGACCTGTTATGCCCCGTTTGTGGTGTGCATTCTGAGCGTGTCCTCCTTTGAATCACAAACGACTCTCGGCAACGGATATCTCGGCTCACGCATCGATGAAGAACGTAGCAAAATGCGATACTTGGTGTGAATTGCAGAATCCCGTGAACCATCGAGTTTTTGAACGCAAGTTGCGCCCGAAGCCATCCGGTTCAGGGCACGCCTGCCTGGGCGTCACGCATCGCGTCGCCCCCCATCATACTTCCCTTAAGGGTAGTCGTGGTGATTGGGAGCGGAGATTGGCTTCCCGTGCTTGTTGTGCGGTTGGTCAAAATAGGAGTCCCCTTCGGTGGACACACGGCTAGTGGTGGTTGTAAAGACCCTTTTCTTCTGCTGTGTGTTGTGAGCTGCTAGGGAAACCCTCAAAAAAGAACCCAATGTATCGTTCTAGGACGATGCTTCGACCGCGACCCCAGGTCAGGCGGGACTACCCGCTGAGTTTAA

>PinedaleALM36

AAGGATCATTGTCGAACCCTGCAAGGCAGAACGACCTGTGAACACGTAAATACAACTGGGTGATGGGGAGATGGATCTTGGTTCTGATCCTCAACACCTTCTAGCGTGCCTGCATGCTTTCTCTTTTGGGCTATCATGCATGTATTGTTGGAATTTAACAAAACCCCGGCACGGCATGTGCCAAGGAAAACAATAAACGAGAAGGACTCGACCTGTTATGCCCCGTTTGTGGTGTGCATTCTGAGCGTGTCCTCCTTTGAATCACAAACGACTCTCGGCAACGGATATCTCGGCTCACGCATCGATGAAGAACGTAGCAAAATGCGATACTTGGTGTGAATTGCAGAATCCCGTGAACCATCGAGTTTTTGAACGCAAGTTGCGCCCGAAGCCATCCGGTTCAGGGCACGCCTGCCTGGGCGTCACGCATCGCGTCGCCCCCCATCATACTTCCCTTAAGGGTAGTCGTGGTGATTGGGAGCGGAGATTGGCTTCCCGTGCTTGTTGTGCGGTTGGTCAAAATAGGAGTCCCCTTCGGTGGACACACGGCTAGTGGTGGTTGTAAAGACCCTTTTCTTCTGCTGTGTGTTGTGAGCTGCTAGGGAAACCCTCAAAAAAGAACCCAATGTATCGTTCTAGGACGATGCTTCGACCGCGACCCCAGGTCAGGCGGGACTACCCGCTGAGTTTAA

>PinedaleALM50

AAGGATCATTGTCGAACCCTGCaAGGCAGAACGACCTGTGAACACGTAAATACAACTGGGTGATGGGGAGATGGATCTTGGTTCTGATCCTCAACACCTYCTAGCGTGCCTGCATGCTTTCTCTTTTGGGCTATCATGCATGTATTGTTGGAATTTAACAAAACCCCGGCACGGCATGTGCCAAGGAAAACAATAAACGAGAAGGACTCGACCTGTTATGCCCCGTTTGTGGTGTGCATTCTGAGCGTGTCCTCCTTTGAATCACAAACGACTCTCGGCAACGGATATCTCGGCTCACGCATCGATGAAGAACGTAGCAAAATGCGATACTTGGTGTGAATTGCAGAATCCCGTGAACCATCGAGTTTTTGAACGCAAGTTGCGCCCGAAGCCATCCGGTTCAGGGCACGCCTGCCTGGGCGTCACGCATCGCGTCGCCCCCCATCATACTTCCCTTAAGGGTAGTCGTGGTGATTGGGAGCGGAGATTGGCTTCCCGTGCTTGTTGTGCGGTTGGTCAAAATAGGAGTCCCCTTCGGTGGACACACGGCTAGTGGTGGTTGTAAAGACCCTTTTCTTCTGCTGTGTGTTGTGAGCTGCTAGGGAAACCCTCAAAAAAGAACCCAATGTATCGTTCTAGGACGATGCTtCGACCGCGACCCCAGGTCAGGCGGGACTACCCGCTGAGTTTAA

>TrentonGAM52

AAGGATCATTGTCGAACCCTGCAAGGCAGAACGACCTGTGAACACGTAAATACAACTGGGTGATGGGGAGATGGATCTTGGTTCTGATCCTCAACACCTCCTAGCGTGCCTGCATGCTTTCTCTTTTGGGCTATCATGCATGTATTGTTGGAATTTAACAAAACCCCGGCACGGCATGTGCCAAGGAAAACAATAAACGAGAAGGACTCGACCTGTTATGCCCCGTTTGTGGTGTGCATTCTGAGCGTGTCCTCCTTTGAATCACAAACGACTCTCGGCAACGGATATCTCGGCTCACGCATCGATGAAGAACGTAGCAAAATGCGATACTTGGTGTGAATTGCAGAATCCCGTGAACCATCGAGTTTTTGAACGCAAGTTGCGCCCGAAGCCATCCGGTTCAGGGCACGCCTGCCTGGGCGTCACGCATCGCGTCGCCCCCCATCATACTTCCCTTAAGGGTAGTCGTGGTGATTGGGAGCGGAGATTGGCTTCCCGTGCTTGTTGTGCGGTTGGTCAAAATAGGAGTCCCCTTCGGTGGACACACGGCTAGTGGTGGTTGTAAAGACCCTTTTCTTCTGCTGTGTGTTGTGAGCTGCTAGGGAAACCCTCAAAAAAGAACCCAATGTATCGTTCTAGGACGATGCTTCGACCGCGACCCCAGGTCAGGCGGGACTACCCGCTGAGTTTAA

>TrentonGAM55

AAGGATCATTGTCGAACCCTGCAAGGCAGAACGACCTGTGAACACGTAAATACAACTGGGTGATGGGGAGATGGATCTTGGTTCTGATCCTCAACACCTCCTAGCGTGCCTGCATGCTTTCTCTTTTGGGCTATCATGCATGTATTGTTGGAATTTAACAAAACCCCGGCACGGCATGTGCCAAGGAAAACAATAAACGAGAAGGACTCGACCTGTTATGCCCCGTTTGTGGTGTGCATTCTGAGCGTGTCCTCCTTTGAATCACAAACGACTCTCGGCAACGGATATCTCGGCTCACGCATCGATGAAGAACGTAGCAAAATGCGATACTTGGTGTGAATTGCAGAATCCCGTGAACCATCGAGTTTTTGAACGCAAGTTGCGCCCGAAGCCATCCGGTTCAGGGCACGCCTGCCTGGGCGTCACGCATCGCGTCGCCCCCCATCATACTTCCCTTAAGGGTAGTCGTGGTGATTGGGAGCGGAGATTGGCTTCCCGTGCTTGTTGTGCGGTTGGTCAAAATAGGAGTCCCCTTCGGTGGACACACGGCTAGTGGTGGTTGTAAAGACCCTTTTCTTCTGCTGTGTGTTGTGAGCTGCTAGGGAAACCCTCAAAAAAGAACCCAATGTATCGTTCTAGGACGATGCTTCGACCGCGACCCCAGGTCAGGCGGGACTACCCGCTGAGTTTAA

>KnoxvilleTNT3

AAGGATCATTGTCGAACCCTGCAAGGCAGAACGACCTGTGAACACGTAAATACAACTGGGTGATGGGGAGATGGATCTTGGTTCTGATCCTCAACACCTCCTAGCGTGCCTGCATGCTTTCTCTTTTGGGCTATCATGCATGTATTGTTGGAATTTAACAAAACCCCGGCACGGCATGTGCCAAGGAAAACAATAAACGAGAAGGACTCGACCTGTTATGCCCCGTTTGTGGTGTGCATTCTGAGCGTGTCCTCCTTTGAATCACAAACGACTCTCGGCAACGGATATCTCGGCTCACGCATCGATGAAGAACGTAGCAAAATGCGATACTTGGTGTGAATTGCAGAATCCCGTGAACCATCGAGTTTTTGAACGCAAGTTGCGCCCGAAGCCATCCGGTTCAGGGCACGCCTGCCTGGGCGTCACGCATCGCGTCGCCCCCCATCATACTTCCCTTAAGGGTAGTCGTGGTGATTGGGAGCGGAGATTGGCTTCCCGTGCTTGTTGTGCGGTTGGTCAAAATAGGAGTCCCCTTCGGTGGACACACGGCTAGTGGTGGTTGTAAAGACCCTTTTCTTCTGCTGTGTGTTGTGAGCTGCTAGGGAAACCCTCAAAAAAGAACCCAATGTATCGTTCTAGGACGATGCTTCGACCGCGACCCCAGGTCAGGCGGGACTACCCGCTGAGTTTAA

>TofficinaleS3

AAGGATCATTGTCGAACCCTGCAAGGCAGaACGACCTGTGAACACGTAAATACAACTGGGTGATGGGGAGATGGATCTTGGTTCTGATCCTCAACACCTCCTAGCGTGCCTGCATGCTTTCTCTTTTGGGCTATCATGCATGTATTGTTGGAATTTAACAAAACCCCGGCACGGCATGTGCCAAGGAAAACAATAAACGAGAAGGACTCGACCTGTTATGCCCCGTTTGTGGTGTGCATTCTGAGCGTGTCCTCCTTTGAATCACAAACGACTCTCGGCAACGGATATCTCGGCTCACGCATCGATGAAGAACGTAGCAAAATGCGATACTTGGTGTGAATTGCAGAATCCCGTGAACCATCGAGTTTTTGAACGCAAGTTGCGCCCGAAGCCATCCGGTTTAGGGCACGCCTGCCTGGGCGTCACGCATCGCGTCGCCCCCCATCATACTTCCCTTAAGGGTAGTCGTGGTGATTGGGAGCGGAGATTGGCTTCCCGTGCTTGTTGTGCGGTTGGTCAAAATAGGAGTCCCCTTCGGTGGACACACGGCTAGTGGTGGTTGTAAAGACCCTTTTCTTCTGCTGTGTGTTGTGAGCTGCTAGGGAAACCCTCAAAAAAGAACCCAATGTATCGTTCTAGGACGATGCTTCGACCGCGACCCCAgGTCAGGCGGGACTACCCGCTGAGTTTAA

>TrentonGAM56

AAGGATCATTGTCGAACCCTGCAAGGCAGAACGACCTGTGAACACGTAAATACAACTGGGTGATGGGGAGATGGATCTTGGTTCTGATCCTCAACACCTCCTAGCGTGCCTGCATGCTTTCTCTTTTGGGCTATCATGCATGTATTGTTGGAATTTAACAAAACCCCGGCACGGCATGTGCCAAGGAAAACAATAAACTAGAAGGACTCGACCTGTTATGCCCCGTTTGTGGTGTGCATTCTGAGCGTGTCCTCCTTTGAATCACAAACGACTCTCGGCAACGGATATCTCGGCTCACGCATCGATGAAGAACGTAGCAAAATGCGATACTTGGTGTGAATTGCAGAATCCCGTGAACCATCGAGTTTTTGAACGCAAGTTGCGCCCGAAGCCATCCGGTTCAGGGCACGCCTGCCTGGGCGTCACGCATCGCGTCGCCCCCCATCATACTTCCCTTAAGGGTAGTCGTGGTGATTGGGAGCGGAGATTGGCTTCCCGTGCTTGTTGTGCGGTTGGTCAAAATAGGAGTCCCCTTCGGTGGACACACGGCTAGTGGTGGTTGTAAAGACCCTTTTCTTCTGCTGTGTGTTGTGAGCTGCTAGGGAAACCCTCAAAAAAGAACCCAATGTATCGTTCTAGGACGATGCTTCGACCGCGACCCCAGGTCAGGCGGGACTACCCGCTGAGTTTAA

>43mileALM62

AAGGATCATTGTCGAACCCTGCAAGGcAGAACGACCTGTGAACACGTAAATACAACTGGGTGATGGGGAGATGGATCTTGGTTCTGATCCTCAACACCTCCTAGCGTGCCTGCATGCTTTCTCTTTTGGGCTATCATGCATGTATTGTTGGAATTTAACAAAACCCCGGCACGGCATGTGCCAAGGAAAACAATAAACKAGAAGGACTCGACCTGTTATGCCCCGTTTGTGGTGTGCATTCTGAGCGTGTCCTCCTTTGAATCACAAACGACTCTCGGCAACGGATATCTCGGCTCACGCATCGATGAAGAACGTAGCAAAATGCGATACTTGGTGTGAATTGCAGAATCCCGTGAACCATCGAGTTTTTGAACGCAAGTTGCGCCCGAAGCCATCCGGTTCAGGGCACGCCTGCCTGGGCGTCACGCATCGCGTCGCCCCCCATCATACTTCCCTTAAGGGTAGTCGTGGTGATTGGGAGCGGAGATTGGCTTCCCGTGCTTGTTGTGCGGTTGGTCAAAATAGGAGTCCCCTTCGGTGGACACACGGCTAGTGGTGGTTGTAAAGACCCTTTTCTTCTGCTGTGTGTTGTGAGCTGCTAGGGAAACCCTCAAAAAAGAACCCAATGTATCGTTCTAGGACGATGCTTCGACCGCGACCCCAGGTCAGGCGGGACTACCCGCTGAGTTTAA

>KnoxvilleTNT1

AAGGATCATTGTCGAACCCTGCAAGGcAGAACGACCTGTGAACACGTAAATACAACTGGGTGATGGGGAGATGGATCTTGGTTCTGATCCTCAACACCTCCTAGCGTGCCTGCATGCTTTCTCTTTTGGGCTATCATGCATGTATTGTTGGAATTTAACAAAACCCCGGCACGGCATGTGCCAAGGAAAACAATAAACGAGAAGGACTCGACCTGTTATGCCCCGTTTGTGGTGTGCATTCTGAGCGTGTCCTCCTTTGAATCACAAACGACTCTCGGCAACGGATATCTCGGCTCACGCATCGATGAAGAACGTAGCAAAATGCGATACTTGGTGTGAATTGCAGAATCCCGTGAACCATCGAGTTTTTGAACGCAAGTTGCGCCCGAAGCCATCCGGTTTAGGGCACGCCTGCCTGGGCGTCACGCATCGCGTCGCCCCCCATCATACTTCCCTTAAGGGTAGTCGTGGTGATTGGGAGCGGAGATTGGCTTCCCGTGCTTGTTGTGCGGTTGGTCAAAATAGGAGTCCCCTTCGGTGGACACACGGCTAGTGGTGGTTGTAAAGACCCTTTTCTTCTGCTGTGTGTTGTGAGCTGCTAGGGAAACCCTCAAATAAGAACCCAATGTATCGTTCTAGGACGATGCTtCGACCGCGACCCCAGGTCAGGCGGGACTACCCGCTGAGTTTAA

>MaryvilleTNT12

AAGGATCATTGTCGAACCCTGCaAGGCAGAACGACCTGTGAACACGTAAATACAACTGGGTGATGGGGAGATGGATCTTGGTTCTGATCCTCAACACCTCCTAGCGTGCCTGCATGCTTTCTCTTTTGGGCTATCATGCATGTATTGTTGGAATTTAACAAAACCCCGGCACGGCATGTGCCAAGGAAAACAATAAACGAGAAGGACTCGACCTGTTATGCCCCGTTTGTGGTGTGCATTCTGAGCGTGTCCTCCTTTGAATCACAAACGACTCTCGGCAACGGATATCTCGGCTCACGCATCGATGAAGAACGTAGCAAAATGCGATACTTGGTGTGAATTGCAGAATCCCGTGAACCATCGAGTTTTTGAACGCAAGTTGCGCCCGAAGCCATCCGGTTTAGGGCACGCCTGCCTGGGCGTCACGCATCGCGTCGCCCCCCATCATACTTCCCTTAAGGGTAGTCGTGGTGATTGGGAGCGGAGATTGGCTTCCCGTGCTTGTTGTGCGGTTGGTCAAAATAGGAGTCCCCTTCGGTGGACACACGGCTAGTGGTGGTTGTAAAGACCCTTTTCTTCTGCTGTGTGTTGTGAGCTGCTAGGGAAACCCTCAAATAAGAACCCAATGTATCGTTCTAGGACGATGCTTCGACCGCGACCCCAGGTCAGGCGGGACTACCCGCTGAGTTTAA

>WelcomeRaMSM31

AAGGATCATTGTCGAACCCTGCAAGGCAGAACGACCTGTGAACACGTAAATACAACTGGGTGATGGGGAGATGGATCTTGGTTCTGATCCTCAACACCTTCTAGCGTGCCTGCATGCTTTCTCTTTTGGGCTATCATGCATGTATTGTTGGAATTTAACAAAACCCCGGCACGGCATGTGCCAAGGAAAACAATAAACGAGAAGGACTCGACCTGTTATGCCCCGTTTGTGGTGTGCATTCTGAGCGTGTCCTCCTTTGAATCACAAACGACTCTCGGCAACGGATATCTCGGCTCACGCATCGATGAAGAACGTAGCAAAATGCGATACTTGGTGTGAATTGCAGAATCCCGTGAACCATCGAGTTTTTGAACGCAAGTTGCGCCCGAAGCCATCCGGTTTAGGGCACGCCTGCCTGGGCGTCACGCATCGCGTCGCCCCCCATCATACTTCCCTTAAGGGTAGTCGTGGTGATTGGGAGCGGAGATTGGCTTCCCGTGCTTGTTGTGCGGTTGGTCAAAATAGGAGTCCCCTTCGGTGGACACACGGCTAGTGGTGGTTGTAAAGACCCTTTTCTTCTGCTGTGTGTTGTGAGCTGCTAGGGAAACCCTCATATAAGAACCCAATGTATCGTTCTAGGACGATGCTTCGACCGCGACCCCAGGTCAGGCGGGACTACCCGCTGAGTTTAA

>WelcomeRaMSM33

AAGGATCATTGTCGAACCCTGCAAGGCAGAACGACCTGTGAACACGTAAATACAACTGGGTGATGGGGAGATGGATCTTGGTTCTGATCCTCAACACCTTCTAGCGTGCCTGCATGCTTTCTCTTTTGGGCTATCATGCATGTATTGTTGGAATTTAACAAAACCCCGGCACGGCATGTGCCAAGGAAAACAATAAACGAGAAGGACTCGACCTGTTATGCCCCGTTTGTGGTGTGCATTCTGAGCGTGTCCTCCTTTGAATCACAAACGACTCTCGGCAACGGATATCTCGGCTCACGCATCGATGAAGAACGTAGCAAAATGCGATACTTGGTGTGAATTGCAGAATCCCGTGAACCATCGAGTTTTTGAACGCAAGTTGCGCCCGAAGCCATCCGGTTTAGGGCACGCCTGCCTGGGCGTCACGCATCGCGTCGCCCCCCATCATACTTCCCTTAAGGGTAGTCGTGGTGATTGGGAGCGGAGATTGGCTTCCCGTGCTTGTTGTGCGGTTGGTCAAAATAGGAGTCCCCTTCGGTGGACACACGGCTAGTGGTGGTTGTAAAGACCCTTTTCTTCTGCTGTGTGTTGTGAGCTGCTAGGGAAACCCTCATATAAGAACCCAATGTATCGTTCTAGGACGATGCTTCGACCGCGACCCCAGGTCAGGCGGGACTACCCGCTGAGTTTAA

>WelcomeRaMSM44

AAGGATCATTGTCGAACCCTGCAAGGCAGAACGACCTGTGAACACGTAAATACAACTGGGTGATGGGGAGATGGATCTTGGTTCTGATCCTCAACACCTTCTAGCGTGCCTGCATGCTTTCTCTTTTGGGCTATCATGCATGTATTGTTGGAATTTAACAAAACCCCGGCACGGCATGTGCCAAGGAAAACAATAAACGAGAAGGACTCGACCTGTTATGCCCCGTTTGTGGTGTGCATTCTGAGCGTGTCCTCCTTTGAATCACAAACGACTCTCGGCAACGGATATCTCGGCTCACGCATCGATGAAGAACGTAGCAAAATGCGATACTTGGTGTGAATTGCAGAATCCCGTGAACCATCGAGTTTTTGAACGCAAGTTGCGCCCGAAGCCATCCGGTTTAGGGCACGCCTGCCTGGGCGTCACGCATCGCGTCGCCCCCCATCATACTTCCCTTAAGGGTAGTCGTGGTGATTGGGAGCGGAGATTGGCTTCCCGTGCTTGTTGTGCGGTTGGTCAAAATAGGAGTCCCCTTCGGTGGACACACGGCTAGTGGTGGTTGTAAAGACCCTTTTCTTCTGCTGTGTGTTGTGAGCTGCTAGGGAAACCCTCATATAAGAACCCAATGTATCGTTCTAGGACGATGCTtCGACCGCGACCCCAGGTCAGGCGGGACTACCCGCTGAGTTTAA

>WelcomeRaMSM48

AAGGATCATTGTCGAACCCTGCAAGGCAGAACGACCTGTGaACACGTAAATACAACTGGGTGATGGGGAGATGGATCTTGGTTCTGATCCTCAACACCTTCTAGCGTGCCTGCATGCTTTCTCTTTTGGGCTATCATGCATGTATTGTTGGAATTTAACAAAACCCCGGCACGGCATGTGCCAAGGAAAACAATAAACGAGAAGGACTCGACCTGTTATGCCCCGTTTGTGGTGTGCATTCTGAGCGTGTCCTCCTTTGAATCACAAACGACTCTCGGCAACGGATATCTCGGCTCACGCATCGATGAAGAACGTAGCAAAATGCGATACTTGGTGTGAATTGCAGAATCCCGTGAACCATCGAGTTTTTGAACGCAAGTTGCGCCCGAAGCCATCCGGTTTAGGGCACGCCTGCCTGGGCGTCACGCATCGCGTCGCCCCCCATCATACTTCCCTTAAGGGTAGTCGTGGTGATTGGGAGCGGAGATTGGCTTCCCGTGCTTGTTGTGCGGTTGGTCAAAATAGGAGTCCCCTTCGGTGGACACACGGCTAGTGGTGGTTGTAAAGACCCTTTTCTTCTGCTGTGTGTTGTGAGCTGCTAGGGAAACCCTCATATAAGAACCCAATGTATCGTTCTAGGACGATGCTTCGACCGCGACCCCAGGTCAGGCGGGAcTACCCGCTGAGTTTAA

>WelcomeRaMSM53

AAGGATCATTGTCGAACCCTGCAAGGCAGAACGACCTGTGaACACGTAAATACAACTGGGTGATGGGGAGATGGATCTTGGTTCTGATCCTCAACACCTTCTAGCGTGCCTGCATGCTTTCTCTTTTGGGCTATCATGCATGTATTGTTGGAATTTAACAAAACCCCGGCACGGCATGTGCCAAGGAAAACAATAAACGAGAAGGACTCGACCTGTTATGCCCCGTTTGTGGTGTGCATTCTGAGCGTGTCCTCCTTTGAATCACAAACGACTCTCGGCAACGGATATCTCGGCTCACGCATCGATGAAGAACGTAGCAAAATGCGATACTTGGTGTGAATTGCAGAATCCCGTGAACCATCGAGTTTTTGAACGCAAGTTGCGCCCGAAGCCATCCGGTTTAGGGCACGCCTGCCTGGGCGTCACGCATCGCGTCGCCCCCCATCATACTTCCCTTAAGGGTAGTCGTGGTGATTGGGAGCGGAGATTGGCTTCCCGTGCTTGTTGTGCGGTTGGTCAAAATAGGAGTCCCCTTCGGTGGACACACGGCTAGTGGTGGTTGTAAAGACCCTTTTCTTCTGCTGTGTGTTGTGAGCTGCTAGGGAAACCCTCATATAAGAACCCAATGTATCGTTCTAGGACGATGCTTCGACCGCGACCCCAGGTCAGGCGGGACTACCCGCTGAGTTTAA

>TrentonGAM15

AAGGATCATTGTCGAACCCTGCAAGGCAGAACGACCTGTGAACACGTAAATACAACTGGGTGATGGGGAGATGGATCTTGGTTCTGATCCTCAACACCTCCTAGCGTGCCTGCATGCTTTCTCTTTTGGGCTATCATGCATGTATTGTTGGAATTTAACAAAACCCCGGCACGGCATGTGCCAAGGAAAACAATAAACTAGAAGGACTCGACCTGTTATGCCCCGTTTGTGGTGTGCATTCTGAGCGTGTCCTCCTTTGAATCACAAACGACTCTCGGCAACGGATATCTCGGCTCACGCATCGATGAAGAACGTAGCAAAATGCGATACTTGGTGTGAATTGCAGAATCCCGTGAACCATCGAGTTTTTGAACGCAAGTTGCGCCCGAAGCCATCCGGTTCAGGGCACGCCTGCCTGGGCGTCACGCATCGCGTCGCCCCCCATCATACTTCCCTTAAGGGTAGTCGTGGTGATTGGGAGCGGAGATTGGCTTCCCGTGCTTGTTGTGCGGTTGGTCAAAATAGGAGTCCCCTTCGGTGGACACACGGCTAGTGGTGGTTGTAAAGACCCTTTTCTTCTGCTGTGTGTTGTGAGCTGCTAGGGAAACCCTCAAAAAAGAACCCAATGTATCGTTCTAGGACGATGCTTCGACCGCGACCCCAGGTCAGGCGGGACTACCCGCTGAGTTTAAGCATATCAATAAAGCGGAGGAA

>TrentonGAM16

AAGGATCATTGTCGAACCCTGCAAGGCAGAACGACCTGTGAACACGTAAATACAACTGGGTGATGGGGAGATGGATCTTGGTTCTGATCCTCAACACCTCCTAGCGTGCCTGCATGCTTTCTCTTTTGGGCTATCATGCATGTATTGTTGGAATTTAACAAAACCCCGGCACGGCATGTGCCAAGGAAAACAATAAACTAGAAGGACTCGACCTGTTATGCCCCGTTTGTGGTGTGCATTCTGAGCGTGTCCTCCTTTGAATCACAAACGACTCTCGGCAACGGATATCTCGGCTCACGCATCGATGAAGAACGTAGCAAAATGCGATACTTGGTGTGAATTGCAGAATCCCGTGAACCATCGAGTTTTTGAACGCAAGTTGCGCCCGAAGCCATCCGGTTCAGGGCACGCCTGCCTGGGCGTCACGCATCGCGTCGCCCCCCATCATACTTCCCTTAAGGGTAGTCGTGGTGATTGGGAGCGGAGATTGGCTTCCCGTGCTTGTTGTGCGGTTGGTCAAAATAGGAGTCCCCTTCGGTGGACACACGGCTAGTGGTGGTTGTAAAGACCCTTTTCTTCTGCTGTGTGTTGTGAGCTGCTAGGGAAACCCTCAAAAAAGAACCCAATGTATCGTTCTAGGACGATGCTTCGACCGCGACCCCAGGTCAGGCGGGACTACCCGCTGAGTTTAAGCATATCAATAAGGCGGAGGAA

>BoligeeALM13

AAGGATCATTGTCGAACCCTGCAAGGCAGAACGACCTGTGAACACGTAAATACAACTGGGTGATGGGGAGATGGATCTTGGTTCTGATCCTCAACGCCTCCTAGCGTGCCTGCATGCTTTCTCTTTTGGGCTATCATGCATGTATTGTTGGAATTTAACAAAACCCCGGCACGGCATGTGCCAAGGAAAACAATAAACGAGAAGGACTCGACCTGTTATGCCCCGTTTGTGGTGTGCATTCTGAGCGTGTCCTCCTTTGAATCACAAACGACTCTCGGCAACGGATATCTCGGCTCACGCATCGATGAAGAACGTAGCAAAATGCGATACTTGGTGTGAATTGCAGAATCCCGTGAACCATCGAGTTTTTGAACGCAAGTTGCGCCCGAAGCCATCCGGTTCAGGGCACGCCTGCCTGGGCGTCACGCATCGCGTCGCCCCCCATCATACTTCCCTTAAGGGTAGTCGTGGTGATTGGGAGCGGAGATTGGCTTCCCGTGCTTGTTGTGCGGTTGGTCAAAATAGGAGTCCCCTTCGGTGGACACACGGCTAGTGGTGGTTGTAAAGACCCTTTTCTTCTGCTGTGTGTTGTGAGCTGCTAGGGAAACCCTCAAAAAAGAACCCAATGTATCGTTCTAGGACGATGCTTCGACC

>WelcomeRaMSM2

AAGGATCATTGTCGAACCCTGCAAGGCAGAACGACCTGTGAACACGTAAATACAACTGGGTGATGGGGAGATGGATCTTGGTTCTGATCCTCAACACCTTCTAGCGTGCCTGCATGCTTTCTCTTTTGGGCTATCATGCATGTATTGTTGGAATTTAACAAAACCCCGGCACGGCATGTGCCAAGGAAAACAATAAACGAGAAGGACTCGACCTGTTATGCCCCGTTTGTGGTGTGCATTCTGAGCGTGTCCTCCTTTGAATCACAAACGACTCTCGGCAACGGATATCTCGGCTCACGCATCGATGAAGAACGTAGCAAAATGCGATACTTGGTGTGAATTGCAGAATCCCGTGAACCATCGAGTTTTTGAACGCAAGTTGCGCCCGAAGCCATCCGGTTTAGGGCACGCCTGCCTGGGCGTCACGCATCGCGTCGCCCCCCATCATACTTCCCTTAAGGGTAGTCGTGGTGATTGGGAGCGGAGATTGGCTTCCCGTGCTTGTTGTGCGGTTGGTCAAAATAGGAGTCCCCTTCGGTGGACACACGGCTAGTGGTGGTTGTAAAGACCCTTTTCTTCTGCTGTGTGTTGTGAGCTGCTAGGGAAACCCTCAAATAAGAACCCAATGTATCGTTCTAGGACGATGCTTCGACCGCGACC

>TrentonGAM7

AAGGATCATTGTCGAACCCTGCAAGGCAGAACGACCTGTGAACACGTAAATACAACTGGGTGATGGGGAGATGGATCTTGGTTCTGATCCTCAACACCTTCTAGCGTGCCTGCATGCTTTCTCTTTTGGGCTATCATGCATGTATTGTTGGAATTTAACAAAACCCCGGCACGGCATGTGCCAAGGAAAACAATAAACGAGAAGGACTCGACCTGTTATGCCCCGTTTGTGGTGTGCATTCTGAGCGTGTCCTCCTTTGAATCACAAACGACTCTCGGCAACGGATATCTCGGCTCACGCATCGATGAAGAACGTAGCAAAATGCGATACTTGGTGTGAATTGCAGAATCCCGTGAACCATCGAGTTTTTGAACGCAAGTTGCGCCCGAAGCCATCCGGTTCAGGGCACGCCTGCCTGGGCGTCACGCATCGCGTCGCCCCCCATCATACTTCCCTTAAGGGTAGTCGTGGTGATTGGGAGCGGAGATTGGCTTCCCGTGCTTGTTGTGCGGTTGGTCAAAATAGGAGTCCCCTTCGGTGGACACACGGCTAGTGGTGGTTGTAAAGACCCTTTTCTTCTGCTGTGTGTTGTGAGCTGCTAGGGAAACCCTCAAATAAGAACCCAATGTATCGTTCTAGGACGATGCTTCGACCG

>KnoxvilleTNT3

AAGGATCATTGTCGAACCCTGCAAGGCAGAACGACCTGTGAACACGTAAATACAACTGGGTGATGGGGAGATGGATCTTGGTTCTGATCCTCAACACCTTCTAGCGTGCCTGCATGCTTTCTCTTTTGGGCTATCATGCATGTATTGTTGGAATTTAACAAAACCCCGGCACGGCATGTGCCAAGGAAAACAATAAACGAGAAGGACTYGACCTGTTATGCCCCGTTTGTGGTGTGCATTCTGAGCGTGTCCTCCTTTGAATCACAAACGACTCTCGGCAACGGATATCTCGGCTCACGCATCGATGAAGAACGTAGCAAAATGCGATACTTGGTGTGAATTGCAGAATCCCGTGAACCATCGAGTTTTTGAACGCAAGTTGCGCCCGAAGCCATCCGGTTCAGGGCACGCCTGCCTGGGCGTCACGCATCGCGTCGCCCCCCATCATACTTCCCTTAAGGGTAGTCGTGGTGATTGGGAGCGGAGATTGGCTTCCCGTGCTTGTTGTGCGGTTGGTCAAAATAGGAGTCCCCTTCGGTGGACACACGGCTAGTGGTGGTTGTAAAGACCCTTTTCTTCTGCTGTGTGTTGTGAGCTGCTAGGGAAACCCTCAAAAAAGAACCCAATGTATCGTTCTAGGACGATGCTTCGACCGCGACCCCAGGTCAGGCGGGGACTACCCGCTGAGTTTAAGCATATCAATAAAGCCGGAGGAA

>BoligeeALM29

AAGGATCATTGTCGAACCCTGCAAGGCAGAACGACCTGTGACACGTAAATACAACTGGGTGATGGGGAGATGGATCTTGGTTCTGATCCTCAACGCCTCCTAGCGTGCCTGCATGCTTTCTCTTTTGGGCTATCATGCATGTATTGTTGGAATTTAACAAAACCCCGGCACGGCATGTGCCAAGGAAAACAATAAACGAGAAGGACTCGACCTGTTATGCCCCGTTTGTGGTGTGCATTCTGAGCGTGTCCTCCTTTGAATCACAAACGACTCTCGGCAACGGATATCTCGGCTCACGCATCGATGAAGAACGTAGCAAAATGCGATACTTGGTGTGAATTGCAGAATCCCGTGAACCATCGAGTTTTTGAACGCAAGTTGCGCCCGAAGCCATCCGGTTCAGGGCACGCCTGCCTGGGCGTCACGCATCGCGTCGCCCCCCATCATACTTCCCTTAAGGGTAGTCGTGGTGATTGGGAGCGGAGATTGGCTTCCCGTGCTTGTTGTGCGGTTGGTCAAAATAGGAGTCCCCTTCGGTGGACACACGGCTAGTGGTGGTTGTAAAGACCCTTTTCTTCTGCTGTGTGTTGTGAGCTGCTAGGGAAACCCTCAAAAAAGAACCCAATGTATCGTTCTAGGACGATGCTtCGACCGCGACCCCAGGTCAGGCGGgGACTACCCGCTGAGTTTAA

>43MileALM39

AAGGATCATTGTCGAACCCTGCAAGGCAGAACGACCTGTGACACGTAAATACAACTGGGTGATGGGGAGATGGATCTTGGTTCTGATCCTCAACRCCTCCTAGCGTGCCTGCATGCTTTCTCTTTTGGGCTATCATGCATGTATTGTTGGAATTTAACAAAACCCCGGCACGGCATGTGCCAAGGAAAACAATAAACGAGAAGGACTCGACCTGTTATGCCCCGTTTGTGGTGTGCATTCTGAGCGTGTCCTCCTTTGAATCACAAACGACTCTCGGCAACGGATATCTCGGCTCACGCATCGATGAAGAACGTAGCAAAATGCGATACTTGGTGTGAATTGCAGAATCCCGTGAACCATCGAGTTTTTGAACGCAAGTTGCGCCCGAAGCCATCCGGTTCAGGGCACGCCTGCCTGGGCGTCACGCATCGCGTCGCCCCCCATCATACTTCCCTTAAGGGTAGTCGTGGTGATTGGGAGCGGAGATTGGCTTCCCGTGCTTGTTGTGCGGTTGGTCAAAATAGGAGTCCCCTTCGGTGGACACACGGCTAGTGGTGGTTGTAAAGACCCTTTTCTTCTGCTGTGTGTTGTGAGCTGCTAGGGAAACCCTCAAAAAAGAACCCAATGTATCGTTCTAGGACGATGCTTCGACCGCGACCCCAgGTCAGGCGGGACTACCCGCTGAGTTTAA

>Tuscaloosa59SraALM47

AAGGATCATTGTCGAACCCTGCAAGGCAGaACGACCTGTGACACGTAAATACAACTGGGTGATGGGGAGATGGATCTTGGTTCTGATCCTCAACACCTCCTAGCGTGCCTGCATGCTTTCTCTTTTGGGCTATCATGCATGTATTGTTGGAATTTAACAAAACCCCGGCACGGCATGTGCCAAGGAAAACAATAAACGAGAAGGACTCGACCTGTTATGCCCCGTTTGTGGTGTGCATTCTGAGCGTGTCCTCCTTTGAATCACAAACGACTCTCGGCAACGGATATCTCGGCTCACGCATCGATGAAGAACGTAGCAAAATGCGATACTTGGTGTGAATTGCAGAATCCCGTGAACCATCGAGTTTTTGAACGCAAGTTGCGCCCGAAGCCATCCGGTTCAGGGCACGCCTGCCTGGGCGTCACGCATCGCGTCGCCCCCCATCATACTTCCCTTAAGGGTAGTCGTGGTGATTGGGAGCGGAGATTGGCTTCCCGTGCTTGTTGTGCGGTTGGTCAAAATAGGAGTCCCCTTCGGTGGACACACGGCTAGTGGTGGTTGTAAAGACCCTTTTCTTCTGCTGTGTGTTGTGAGCTGCTAGGGAAACCCTCAAAAAAGAACCCAATGTATCGTTCTAGGACGATGCTTCGACCGCGACCCCAGGTCAGGCGGGaCTACCCGCTGAGTTTAA

>KnoxvilleTN53

AAGGATCATTGTCGAACCCTGCAAGGCAGAACGACCTGTGAACACGTAAATACAACTGGGTGATGGGGAGATGGATCTTGGTTCTGATCCTCAACACCTCCTAGCGTGCCTGCATGCTTTCTCTTTTGGGCTATCATGCATGTATTGTTGGAATTTAACAAAACCCCGGCACGGCATGTGCCAAGGAAAACAATAAACGAGAAGGACTCGACCTGTTATGCCCCGTTTGTGGTGTGCATTCTGAGCGTGTCCTCCTTTGAATCACAAACGACTCTCGGCAACGGATATCTCGGCTCACGCATCGATGAAGAACGTAGCAAAATGCGATACTTGGTGTGAATTGCAGAATCCCGTGAACCATCGAGTTTTTGAACGCAAGTTGCGCCCGAAGCCATCCGGTTTAGGGCACGCCTGCCTGGGCGTCACGCATCGCGTCGCCCCCCATCATACTTCCCTTAAGGGTAGTCGTGGTGATTGGGAGCGGAGATTGACTTCCTGTGCTTGTTGTGCGGTTGGTCAAAATAGGAGTCCCCTTCGGTGGACACACGGCTAGTGGTGGTTGTAAAGACCCTTTTCTTCTGCTGTGTGTTGTGAGCTGCTAGGGAAACCCTCAAAAAAGAACCCAATGTATCGTTCTAGGACGATGCTTCGACCGCGACCCCAGGTCAGGCGGGACTACCCCGCTGAGTTTAA

>WelcomeRaMSM5

AAGGATCATTGTCGAACCCTGCAAGGCAGAACGACCTGTGAACACGTGAAATACAACTGGGTGATGGGGAGATGGATCTTGGTTCTGATCCTCAACACCTTCTAGCGTGCCTGCATGCTTTCTCTTTTGGGCTATCATGCATGTATTGTTGGAATTTAACAAAACCCCGGCACGGCATGTGCCAAGGAAAACAATAAACGAGAAGGACTCGACCTGTTATGCCCCGTTTGTGGTGTGCATTCTGAGCGTGTCCTCCTTTGAATCACAAACGACTCTCGGCAACGGATATCTCGGCTCACGCATCGATGAAGAACGTAGCAAAATGCGATACTTGGTGTGAATTGCAGAATCCCGTGAACCATCGAGTTTTTGAACGCAAGTTGCGCCCGAAGCCATCCGGTTTAGGGCACGCCTGCCTGGGCGTCACGCATCGCGTCGCCCCCCATCATACTTCCCTTAAGGGTAGTCGTGGTGATTGGGAGCGGAGATTGGCTTCCCGTGCTTGTTGTGCGGTTGGTCAAAATAGGAGTCCCCTTCGGTGGACACACGGCTAGTGGTGGTTGTAAAGACCCTTTTCTTCTGCTGTGTGTTGTGAGCTGCTAGGGAAACCCTCAAAAAAGAACCCAATGTATCGTTCTAGGACGATGCTTCGACCGCGACCCCAGGTCAGGCGGGACTACCCGCTGAGTTTAAGCATATCAATAAGCGGAA

>BoligeeALM22

AAGGATCATTGTCGAACCCTGCAAGGCAGAACGACCTGTGaACACGTgAAATACAACTGGGTGATGGGGAGATGGATCTTGGTTCTGATCCTCAACGCCTCCTAGCGTGCCTGCATGCTTTCTCTTTTGGGCTATCATGCATGTATTGTTGGAATTTAACAAAACCCCGGCACGGCATGTGCCAAGGAAAACAATAAACGAGAAGGACTCGACCTGTTATGCCCCGTTTGTGGTGTGCATTCTGAGCGTGTCCTCCTTTGAATCACAAACGACTCTCGGCAACGGATATCTCGGCTCACGCATCGATGAAGAACGTAGCAAAATGCGATACTTGGTGTGAATTGCAGAATCCCGTGAACCATCGAGTTTTTGAACGCAAGTTGCGCCCGAAGCCATCCGGTTCAGGGCACGCCTGCCTGGGCGTCACGCATCGCGTCGCCCCCCATCATACTTCCCTTAAGGGTAGTCGTGGTGATTGGGAGCGGAGATTGGCTTCCCGTGCTTGTTGTGCGGTTGGTCAAAATAGGAGTCCCCTTCGGTGGACACACGGCTAGTGGTGGTTGTAAAGACCCTTTTCTTCTGCTGTGTGTTGTGAGCTGCTAGGGAAACCCTCAAAAAAGAACCCAATGTATCGTTCTAGGACGATGCTTCGACCGCGACCCCAGGTCAGGCGGGACTACCCGCTGAGTTTAA

>43mileALM25

AAGGATCATTGTCGAACCCTGCAAGGCAGACGACCTGTGACACGTAAATACAATTGGGTGATGGGGAGATGGATCTTGGTTCTGATCCTCAACACCTCCTAGCGTGCCTGCATGCTTTCTCTTTTGGGCTATCATGCATGTATTGTTGGAATTTAACAAAACCCCGGCACGGCATGTGCCAAGGAAAACAATAAACTAGAAGGACTCGACCTGTTATGCCCCGTTTGTGGTGTGCATTCTGAGCGTGTCCTCCTTTGAATCACAAACGACTCTCGGCAACGGATATCTCGGCTCACGCATCGATGAAGAACGTAGCAAAATGCGATACTTGGTGTGAATTGCAGAATCCCGTGAACCATCGAGTTTTTGAACGCAAGTTGCGCCCGAAGCCATCCGGTTCAGGGCACGCCTGCCTGGGCGTCACGCATCGCGTCGCCCCCCATCATACTTCCCTTAAGGGTAGTCGTGGCGATTGGGAGCGGAGATTGGCTTCCCGTGCTTGTTGTGCGGTTGGTCAAAATAGGAGTCCCCTTCGGTGGACACACGGCTAGTGGTGGTTGTAAAGACCCTTTTCTTCTGCTGTGTGTTGTGAGCTGCTAGGGAAACCCTCAAAAAAGAACCCAATGTATCGTTCTAGGACGATGCTTCGACCGCGACCCCAGGTCAGGCGGGACTACCCGCTGAGTTTAA

>PinedaleALM1

CATCGATGCGTGAGCCGAGATATCCCGTTGCCGAGAGTCGTTTGTGATTCAAAGGAGGGACACGCTCAGAATGCACACCACAAACGGGGCATAACAGGTCGAGTCCTTCTAGTTTATTGTTTTCCTTGGCACATGCCGTGCCGGGGTTTTGTTAAATTCCAACAATACATGCATGATAGCCCAAAAGAGAAAGCATGCAGGCACGCTAGGAGGTGTTGAGGATCAGAACCAAGATCCATCTCCCCATCACCCAATTGTATTTACGTGTTCACAGGTCGTTCTGCCTTGCAGGGTTCGACAATGATCCTTCCGTAGGTGAACCTGCGGAAGGATCATTGTCGAACCCTGCAAGGCAGAACGACCTGTGAACACGTAAATACAATTGGGTGATGGGGAGATGGATCTTGGTTCTGATCCTCAACACCTCCTAGCGTGCCTGCATGCTTTCTCTTTTGGGCTATCATGCATGTATTGTTGGAATTTAACAAAACCCCGGCACGGCATGTGCCAAGGAAAACAATAAACTAGAAGGACTCGACCTGTTATGCCCCGTTTGTGGTGTGCATTCTGAGCGTGTCCTCCTTTGAATCACAAACGACTCTCGGCAACGGATATCTCGGCTCACGCATCGATGAAGAACGTAGCAAAATGCGATACTTGGTGTGAATTGCAGAATCCCGTGAACCATCGAGTTTTTGAACGCAAGTTGCGCCCGAAGCCATCCGGTTCAGGGCACGCCTGCCTGGGCGTCACGCATCGCGTCGCCCCCCATCATACTTCCCTTAAGGGTAGTCGTGGCGATTGGGAGCGGAGATTGGCTTCCCGTGCTTGTTGTGCGGTTGGTCAAAATAGGAGTCCCCTTCGGTGGACACACGGCTAGTGGTGGTTGTAAAGACCCTTTTCTTCTGCTGTGTGTTGTGAGCTGCTAGGGAAACCCTCAAAAAAGAACCCAATGTATCGTTCTAGGACGATGCTTCGACCGCGACCCCAGGTCAGGCGGGACTACCCGCTGAGTTTAAGCATATCAATAAAGCGGAGGAA

>KnoxvilleTNT4

AAGGATCATTGTCGAACCCTGCAAGGCAGAACGACCTGTGAACACGTAAATACAATTGGGTGATGGGGAGATGGATCTTGGTTCTGATCCTCAACACCTCCTAGCGTGCCTGCATGCTTTCTCTTTTGGGCTATCATGCATGTATTGTTGGAATTTAACAAAACCCCGGCACGGCATGTGCCAAGGAAAACAATAAACTAGAAGGACTCGACCTGTTATGCCCCGTTTGTGGTGTGCATTCTGAGCGTGTCCTCCTTTGAATCACAAACGACTCTCGGCAACGGATATCTCGGCTCACGCATCGATGAAGAACGTAGCAAAATGCGATACTTGGTGTGAATTGCAGAATCCCGTGAACCATCGAGTTTTTGAACGCAAGTTGCGCCCGAAGCCATCCGGTTCAGGGCACGCCTGCCTGGGCGTCACGCATCGCGTCGCCCCCCATCATACTTCCCTTAAGGGTAGTCGTGGCGATTGGGAGCGGAGATTGGCTTCCCGTGCTTGTTGTGCGGTTGGTCAAAATAGGAGTCCCCTTCGGTGGACACACGGCTAGTGGTGGTTGTAAAGACCCTTTTCTTtCTGCTGTGTGTTGTGAGCTGCTAGGGAAACCCTCAAAAAAGAACCCAATGTATCGTTCTAGGACGATGCTTCGACCGCGACCCCAGGTCAGGCGGGACTACCCGCTGAGTTTAT

>WelcomeRaMSM27

AAGGATCATTGTCGAACCCTGCaAGGCAGAACGACCtTGtTGAACACGTAAATACAACTGGGTGATGGGGAGATGGATCTTGGTTCTGATCCTCAACACCTTCTAGCGTGCCTGCATGCTTTCTCTTTTGGGCTATCATGCATGTATTGTTGGAATTTAACAAAACCCCGGCACGGCATGTGCCAAGGAAAACAATAAACGAGAAGGACTCGACCTGTTATGCCCCGTTTGTGGTGTGCATTCTGAGCGTGTCCTCCTTTGAATCACAAACGACTCTCGGCAACGGATATCTCGGCTCACGCATCGATGAAGAACGTAGCAAAATGCGATACTTGGTGTGAATTGCAGAATCCCGTGAACCATCGAGTTTTTGAACGCAAGTTGCGCCCGAAGCCATCCGGTTTAGGGCACGCCTGCCTGGGCGTCACGCATCGCGTCGCCCCCCATCATACTTCCCTTAAGGGTAGTCGTGGTGATTGGGAGCGGAGATTGGCTTCCCGTGCTTGTTGTGCGGTTGGTCAAAATAGGAGTCCCCTTCGGTGGACACACGGCTAGTGGTGGTTGTAAAGACCCTTTTCTTCTGCTGTGTGTTGTGAGCTGCTAGGGAAACCCTCATATAAGAACCCAATGTATCGTTCTAGGACGATGCTTCGACCGCGACCCCAGGTCAGGCGGGACTACCCGCTGAGTTTAA

>WelcomeRaMSM4

TCCCCATCACCCAGTTGTATTTACGTGTTCACAGGTCGTTCTGCCTTGCAGGGTTCGACAATGATCCTTCCGTAGGTGAACCTGCGGAAGGATCATTGTCGAACCCTGCAAGGCCGAACGACCTGTGAACACGTAAATACAACTGGGTGATGGGGAGATGGATCTTGGTTCTGATCCTCAACACCTTCTAGCGTGCCTGCATGCTTTCTCTTTTGGGCTATCATGCATGTATTGTTGGAATTTAACAAAACCCCGGCACGGCATGTGCCAAGGAAAACAATAAACGAGAAGGACTCGACCTGTTATGCCCCGTTTGTGGTGTGCATTCTGAGCGTGTCCTCCTTTGAATCACAAACGACTCTCGGCAACGGATATCTCGGCTCACGCATCGATGAAGAACGTAGCAAAATGCGATACTTGGTGTGAATTGCAGAATCCCGTGAACCATCGAGTTTTTGAACGCAAGTTGCGCCCGAAGCCATCCGGTTTAGGGCACGCCTGCCTGGGCGTCACGCATCGCGTCGCCCCCCATCATACTTCCCTTAAGGGTAGTCGTGGTGATTGGGAGCGGAGATTGGCTTCCCGTGCTTGTTGTGCGGTTGGTCAAAATAGGAGTCCCCTTCGGTGGACACACGGCTAGTGGTGGTTGTAAAGACCCTTTTCTTCTGCTGTGTGTTGTGAGCTGCTAGGGAAACCCTCAAAAAAGAACCCAATGTATCGTTCTAGGACGATGCTTCGACCGCGACCCCAGGTCAGGCGGGACTACCCGCTGAGTTTAA

>TofficinaleS1

AAGGATCATTGTCGAACCCTGCAAGGCAGAACGACCTGTGAACACGTAAATACAACTGGGTGATGGGGAGATGGATCTTGGTTCTGATCCTCAACACCTCCTAGCGTGCCTGCATGCTTTCTCTTTTGGGCTATCATGCATGTATTGTTGGAATTTAACAAAACCCCGGCACGGCATGTGCCAAGGAAAACAATAAACGAGAAGGACTCGACCTGTTATGCCCCGTTTGTGGTGTGCATTCTGAGCGTGTCCTCCTTTGAATCACAAACGACTCTCGGCAACGGATATCTCGGCTCACGCATCGATGAAGAACGTAGCAAAATGCGATACTTGGTGTGAATTGCAGAATCCCGTGAACCATCGAGTTTTTGAACGCAAGTTGCGCCCGAAGCCATCCGGTTTAGGGCACGCCTGCCTGGGCGTCACGCATCGCGTCGCCCCCCATCATACTTCCCTTAAGGGTAgTCGTGGTGATTGGGAGCGGAGATTGGCTTCCCGTGCTTGTGTGCGGtTGGtCAAAATAGGAGTCCCCTTCGGtGGACACACGGCTAGTGGtGGtTGTAAAGACCCTTTTCTTCTGCTGTGTGTGTGAGCTGCTAGGGAAACCCTCAAAAAAGAACCCAATGTATCGTTCTAGGACGATGCTTCGACCGCGACCCCAGgTCAGGCGGGACTACCCGCTGAGTTTAA

>BoligeeALM20

CCTTCCGTAGGTGACCTGCGGAAGGATCATTGTCGAACCCTGCAAGGcAgACGACCTcGTGACACGTAAATACAACTcGGGTGATGGGGAGATGGATCTTGATTCTGATCCTCAACACCTCCTAGCGTGCCTGCATGCTTTCTCTTTTGGGCTATCATGCATGTATTGTTGGAATTTAACAAAACCCCGGCACGGCATGTGCCAAGGAAAACAATAAACGAGAAGGACTCGACCTGTTATGCCCCGTTTGTGGTGTGCATTCTGAGCGTGTCCTCCTTTGAATCACAAACGACTCTCGGCAACGGATATCTCGGCTCACGCATCGATGAAGAACGTAGCAAAATGCGATACTTGGTGTGAATTGCAGAATCCCGTGAACCATCGAGTTTTTGAACGCAAGTTGCGCCCGAAGCCATCCGGTTCAGGGCACGCCTGCCTGGGCGTCACGCATCGCGTCGCCCCCCATCATACTTCCCTTAAGGGTAGTCGTGGTGATTGGGAGCGGAGATTGGCTTCCCGTGCTTGTTGTGCGGTTGGTCAAAATAGGAGTCCCCTTCGGTGGACACATGGCTAGTGGTGGTTGTAAAGACCCTTTTCTTCTGCTGTGTGTTGTGAGCTGCTAGGGAAACCCTCAAAAAAGACACCCAATGTATCGTTCTAGGACGATGCTTCGACCGCGACCCCAGGTCAGGCGGGACTACCCGCTGAGTTTAA

>Tuscaloosa59SraALM65

AAGGATCATTGTCGAACCCTGCATGGCAGaACGACCTGTGAACACGTAAATACAACTcGGGTGATGGGGAGATGGATCTTGATTCTGATCCTCAACACCTCCTAGCGTGCCTGCATGCTTTCTCTTTTGGGCTATCATGCATGTATTGTTGGAATTTAACAAAACCCCGGCACGGCATGTGCCAAGGAAAACAATAAATGAGAAGGACTCGACCTGTTATGCCCCGTTTGTGGTGTGCATTCTGAGCGTGTCCTCCTTTGAATCACAAACGACTCTCGGCAACGGATATCTCGGCTCACGCATCGATGAAGAACGTAGCAAAATGCGATACTTGGTGTGAATTGCAGAATCCCGTGAACCATCGAGTTTTTGAACGCAAGTTGCGCCCGAAGCCATCCGGTTCAGGGCACGCCTGCCTGGGCGTCACGCATCGCGTCGCCCCCCATCATACTTCCCTTAAGGGTAGTCGTGGTGATTGGGAGCGGAGATTGGCTTCCCGTGCTTGTGGTGCGGTTGGTCAAAATAGGAGTCCCCTTCGGTGGACACACGGCTAGTGGTGGTTGTAAAGACCCTTTTCTTCTGCTGTGTGTTGTGAGCTGCTAGGGAAACCCTCAAAAAAGACACCCAATGTATCGTTCTAGGACGATGCTTCGACCGCGACCCCAGGTCAGGCGGGACTACCCGCTGAGTTTAA

>MaryvilleTNK6AAGGATCATNGTCGAACCCTGCATGGCAGAACGACCTGTGTACATGTAAATACAATCGGGTGATGGGGAAATGGATCTTGGTTCTGATCCTCAACACCTCCTAGCGTGCCTGCATGCTTTCTCTTTTGGGCTATCATGCATGTATTGTTGGAATTTAACAAAACCCCGGCACGGCATGTGCCAAGGAAAACAAAAAACGAGAAGGACTCGACCTGTAATGCCCCGTTTGTGGTGTGCATTCTGAGCGTGTCCTCCTTTGAATCACAAACGACTCTCGGCAACGGATATCTCGGCTCACGCATCGATGAAGAACGTAGCAAAATGCGATACTTGGTGTGAATTGCAGAATCCCGTGAACCATCGAGTTTTTGAACGCAAGTTGCGCCTGAAGCCATCCGGTTGAGGGCACGCCTGCCTGGGCGTCACGCATCGCGTTGCCCCCCATCATACTTCCCTTAAGGGTAGTCGTGGTGATTGGGAGCGGAGATTGGCCTCCCGTACTTGTGGTGCGGTTGGTCAAAATAGGAGTCCCCTTCGGTGGACACACGGCTAGTGGTGGTTGTAAAGACCCTTTTCTTCTGCTGTGTGTTGTGAGCTGTTTGGGAAACCCTCAAAAAAGACCCCAATGTGTCGTTCTAGGATGATAT

>PinedaleALM12

AAGGATCATTGTCGAACCCTGCATGGCAGAACGACCTGTGAACATGTAAATACAATCGGGTGATGGGGAAATGGATCTTGGTTCTGATCCTCAACACCTCCTAGCGTGCCTGCATGCTTTCTCTTTTGGGCTATCATGCATGTATTGTTGGAATTTAACAAAACCCCGGCACGGCATGTGCCAAGGAAAACAAAAAACGAGAAGGACTCGACCTGTAATGCCCCGTTTGTGGTGTGCATTCTGAGCGTGTCCTCCTTTGAATCACAAACGACTCTCGGCAACGGATATCTCGGCTCACGCATCGATGAAGAACGTAGCAAAATGCGATACTTGGTGTGAATTGCAGAATCCCGTGAACCATCGAGTTTTTGAACGCAAGTTGCGCCTGAAGCCATCCGGTTGAGGGCACGCCTGCCTGGGCGTCACGCATCGCGTTGCCCCCCATCATACTTCCCTTAAGGGTAGTCGTGGTGATTGGGAGCGGAGATTGGCCTCCCGTACTTGTGGTGCGGTTGGTCAAAATAGGAGTCCCCTTCGGTGGACACACGGCTAGTGGTGGTTGTAAAGACCCTTTTCTTTTGCTGTGTGTTGTGAGCTGTTTGGGAAACCCTCAAATAAGACCCCAATGTGTCGTTCTAGGATGATACTTCGACCGCGACCCCAGGTCAGGCGGGACTACCCGCTGAGTTTAAGCATATCAATAAGGCGGAGGAA

>PinedaleALM26

AAGGATCATTGTCGAACCCTGCATGGCAGAACGACCTGTGaACATGTAAATACAATtCGGGTGATGGGGAAATGGATCTTGGTTCTGATCCTCAACACCTCCTAGCGTGCCTGCATGCTTTCTCTTTTGGGCTATCATGCATGTATTGTTGGAATTTAACAAAACCCCGGCACGGCATGTGCCAAGGAAAACAAAAAACGAGAAGGACTCGACCTGTAATGCCCCGTTTGTGGTGTGCATTCTGAGCGTGTCCTCCTTTGAATCACAAACGACTCTCGGCAACGGATATCTCGGCTCACGCATCGATGAAGAACGTAGCAAAATGCGATACTTGGTGTGAATTGCAGAATCCCGTGAACCATCGAGTTTTTGAACGCAAGTTGCGCCTGAAGCCATCCGGTTGAGGGCACGCCTGCCTGGGCGTCACGCATCGCGTTGCCCCCCATCATACTTCCCTTAAGGGTAGTCGTGGTGATTGGGAGCGGAGATTGGCCTCCCGTACTTGTGGTGCGGTTGGTCAAAATAGGAGTCCCCTTCGGTGGACACACGGCTAGTGGTGGTTGTAAAGACCCTTTTCTTTTGCTGTGTGTTGTGAGCTGTTTGGGAAACCCTCAAAAAAGACCCCAATGTGTCGTTCTAGGATGATACTTCGACCGCGACCCcAgGTCAGGCGGGACTACCCGCTGAGTTTAA

>MaryvilleTNT6

AAGGATCATTGTCGAACCCTGCATGGCAGAACGACCTGTGAACATGTAAATACAATcCGGGTGATGGGGAAATGGATCTTGGTTCTGATCCTCAACACCTCCTAGCGTGCCTGCATGCTTTCTCTTTTGGGCTATCATGCATGTATTGTTGGAATTTAACAAAACCCCGGCACGGCATGTGCCAAGGAAAACAAAAAACGAGAAGGACTCGACCTGTAATGCCCCGTTTGTGGTGTGCATTCTGAGCGTGTCCTCCTTTGAATCACAAACGACTCTCGGCAACGGATATCTCGGCTCACGCATCGATGAAGAACGTAGCAAAATGCGATACTTGGTGTGAATTGCAGAATCCCGTGAACCATCGAGTTTTTGAACGCAAGTTGCGCCTGAAGCCATCCGGTTGAGGGCACGCCTGCCTGGGCGTCACGCATCGCGTTGCCCCCCATCATACTTCCCTTAAGGGTAGTCGTGGTGATTGGGAGCGGAGATTGGCCTCCCGTACTTGTGGTGCGGTTGGTCAAAATAGGAGTCCCCTTCGGTGGACACACGGCTAGTGGTGGTTGTAAAGACCCTTTTCTTTTGCTGTGTGTTGTGAGCTGTTTGGGAAACCCTCAAAAAAGACCCCAATGTGTCGTTCTAGGATGATACTTCGACCGCGACCCCAGGTCAGGCGGGACTACCCGCTGAGTTTAA

>MaryvilleTNT10

AAGGATCATTGTCGAACCCTGCATGGCAGAACGACCTGTGAACATGTAAATACAATCGGGTGATGGGGAAATGGATCTTGGTTCTGATCCTCAACACCTCCTAGCGTGCCTGCATGCTTTCTCTTTTGGGCTATCATGCATGTATTGTTGGAATTTAACAAAACCCCGGCACGGCATGTGCCAAGGAAAACAAAAAACGAGAAGGACTCGACCTGTAATGCCCCGTTTGTGGTGTGCATTCTGAGCGTGTCCTCCTTTGAATCACAAACGACTCTCGGCAACGGATATCTCGGCTCACGCATCGATGAAGAACGTAGCAAAATGCGATACTTGGTGTGAATTGCAGAATCCCGTGAACCATCGAGTTTTTGAACGCAAGTTGCGCCTGAAGCCATCCGGTTGAGGGCACGCCTGCCTGGGCGTCACGCATCGCGTTGCCCCCCATCATACTTCCCTTAAGGGTAGTCGTGGTGATTGGGAGCGGAGATTGGCCTCCCGTACTTGTGGTGCGGTTGGTCAAAATAGGAGTCCCCTTCGGTGGACACACGGCTAGTGGTGGTTGTAAAGACCCTTTTCTTTTGCTGTGTGTTGTGAGCTGTTTGGGAAACCCTCAAAAAAGACCCCAATGTGTCGTTCTAGGATGATACTTCGACCGCGACCCCAGGTCAGGCGGGACTACCCGCTGAGTTTAA

>MaryvilleTNT9

AAGGATCATTGTCGAACCCTGCATGGCAGaACGACCTGTGaACATGTAAATACAATCGGGTGATGGGGAAATGGATCTTGGTTCTGATCCTCAACACCTCCTAGCGTGCCTGCATGCTTTCTCTTTTGGGCTATCATGCATGTATTGTTGGAATTTAACAAAACCCCGGCACGGCATGTGCCAAGGAAAACAAAAAACGAGAAGGACTCGACCTGTAATGCCCCGTTTGTGGTGTGCATTCTGAGCGTGTCCTCCTTTGAATCACAAACGACTCTCGGCAACGGATATCTCGGCTCACGCATCGATGAAGAACGTAGCAAAATGCGATACTTGGTGTGAATTGCAGAATCCCGTGAACCATCGAGTTTTTGAACGCAAGTTGCGCCTGAAGCCATCCGGTTGAGGGCACGCCTGCCTGGGCGTCACGCATCGCGTTGCCCCCCATCATACTTCCCTTAAGGGTAGTCGTGGTGATTGGGAGCGGAGATTGGCCTCCCGTACTTGTGGTGCGGTTGGTCAAAATAGGAGTCCCCTTCGGTGGACACACGGCTAGTGGTGGTTGTAAAGACCCTTTTCTTTTGCTGTGTGTTGTGAGCTGTTTGGGAAaCCCTCAAAAAAGACCCCAATGTGTCGTTCTAGGATGATACTTCGACCGCGACCCCAGGTCAGGCGGGACTACCCGCTGAGTTTAA

>MaryvilleTNT11

AAGGATCATTGTCGAACCCTGCATGGCAGAACGACCTGTGAACATGTAAATACAATCGGGTGATGGGGAAATGGATCTTGGTTCTGATCCTCAACACCTCCTAGCGTGCCTGCATGCTTTCTCTTTTGGGCTATCATGCATGTATTGTTGGAATTTAACAAAACCCCGGCACGGCATGTGCCAAGGAAAACAAAAAACGAGAAGGACTCGACCTGTAATGCCCCGTTTGTGGTGTGCATTCTGAGCGTGTCCTCCTTTGAATCACAAACGACTCTCGGCAACGGATATCTCGGCTCACGCATCGATGAAGAACGTAGCAAAATGCGATACTTGGTGTGAATTGCAGAATCCCGTGAACCATCGAGTTTTTGAACGCAAGTTGCGCCTGAAGCCATCCGGTTGAGGGCACGCCTGCCTGGGCGTCACGCATCGCGTTGCCCCCCATCATACTTCCCTTAAGGGTAGTCGTGGTGATTGGGAGCGGAGATTGGCCTCCCGTACTTGTGGTGCGGTTGGTCAAAATAGGAGTCCCCTTCGGTGGACACACGGCTAGTGGTGGTTGTAAAGACCCTTTTCTTTTGCTGTGTGTTGTGAGCTGTTTGGGAAACCCTCAAAAAAGACCCCAATGTGTCGTTCTAGGATGATACTtCGACCGCGACCCCAGGTCAGGCGGGACTACCCGCTGAGTTTAA

>MaryvilleTNT2

AAGGATCATTGTCGAACCCTGCATGGCAGAACGACCTGTGAACATGTAAATACAATCGGGTGATGGGGAAATGGATCTTGGTTCTGATCCTCAACACCTCCTAGCGTGCCTGCATGCTTTCTCTTTTGGGCTATCATGCATGTATTGTTGGAATTTAACAAAACCCCGGCACGGCATGTGCCAAGGAAAACAAAAAACGAGAAGGACTCGACCTGTAATGCCCCGTTTGTGGTGTGCATTCTGAGCGTGTCCTCCTTTGAATCACAAACGACTCTCGGCAACGGATATCTCGGCTCACGCATCGATGAAGAACGTAGCAAAATGCGATACTTGGTGTGAATTGCAGAATCCCGTGAACCATCGAGTTTTTGAACGCAAGTTGCGCCTGAAGCCATCCGGTTGAGGGCACGCCTGCCTGGGCGTCACGCATCGCGTTGCCCCCCATCATACTTCCCTTAAGGGTAGTCGTGGTGATTGGGAGCGGAGATTGGCCTCCCGTACTTGTGGTGCGGTTGGTCAAAATAGGAGTCCCCTTCGGTGGACACACGGCTAGTGGTGGTTGTAAAGACCCTTTTCTTTTGCTGTGTGTTGTGAGCTGTTTGGGAAACCCTCAAAAAAGACCCCAATGTGTCGTTCTAGGATGATACTtCGACCGCGACCCCAGGTCAGGCGGGACTACCCGCTGAGTTTTAt

>TofficinaleHerbariumW413345

AAGGATCATTGTCGAACCCTGCATGGCAGAACGACCTGTGAACATGTAAATACAATCGGGTGATGGGGAAATGGATCTTGGTTCTGATCCTCAACACCTCCTAGCGTGCCTGCATGCTTTCTCTTTTGGGCTATCATGCATGTATTGTTGGAATTTAACAAAACCCCGGCACGGCATGTGCCAAGGAAAACAAAAAACGAGAAGGACTCGACCTGTAATGCCCCGTTTGTGGTGTGCATTCTGAGCGTGTCCTCCTTTGAATCACAAACGACTCTCGGCAACGGATATCTCGGCTCACGCATCGATGAAGAACGTAGCAAAATGCGATACTTGGTGTGAATTGCAGAATCCCGTGAACCATCGAGTTTTTGAACGCAAGTTGCGCCTGAAGCCATCCGGTTGAGGGCACGCCTGCCTGGGCGTCACGCATCGCGTTGCCCCCCATCATACTTCCCTTAAGGGTAGTCGTGGTGATTGGGAGCGGAGATTGGCtCTCCCGTACTTGTGGTGCGGTTGGTCAAAATAGGAGTCCCCTTCGGTGGACACACGGCTAGTGGTGGTTGTAAAGACCCTTTTCTTTTGCTGTGTGTTGTGAGCTGTTTGGGAAACCCTCAAAAAAGACCCCAATGTGTCGTTCTAGGATGATACTTCGACCGCGACCCCAaGGTCAGGCGGGACTACCCGCTGAGTTTAA

>MaryvilleTNT5

AAGGATCATTGTCGAACCCTGCATGGCAGAACGACCTGTGAACATGTAAATACAATtCGGGTGATGGGGAGATGGATCTTGGTTCTGATCCTCAACACCTCCTAGCGTGCCTGCATGCTTTCTCTTTTGGGCTATCATGCATGTATTGTTGGAATTTAACAAAACCCCGGCACGGCATGTGCCAAGGAAAACAATAAACGAGAAGGACTCGACCTGTAATGCCCCGTTTGTGGTGTGCATTCTGAGCGTGTCCTCCTTTGAATCACAAACGACTCTCGGCAACGGATATCTCGGCTCACGCATCGATGAAGAACGTAGCAAAATGCGATACTTGGTGTGAATTGCAGAATCCCGTGAACCATCGAGTTTTTGAACGCAAGTTGCGCCTGAAGCCATCCGGTTGAGGGCACGCCTGCCTGGGCGTCACGCATCGCGTTGCCCCCCATCATACTTCCCTTAAGGGTAGTCGTGGTGATTGGGAGCGGAGATTGGCCTCCCGTGACTTGTGGTGCGGTTGGTCAAAATAGGAGTCCCCTTCGGTGGACACACGGCTAGTGGTGGTTGTAAAGACCCTTTtCTTCTGCTGTGTGTTGTGAGCTGCTAGGGAAACCCTCAAAAAAGACCCCAATGTGTCGTTCTAGGATGATACTTCGACTGCGACCCCAGGTCAGGCGGGACTACCCGCTGAGTTTAA

>PinedaleALM57

AAGGATCATTGTCGAACCCTGCATGGCAGaACGACCTGTGACATGTAAATACAATTCGGGTGATGGGGAAATGGATCTTGGTTCTGATCCTCAACACCTCCTAGCGTGCCTGCATGCTTTCTCTTTTGGGCTATCATGCATGTATTGTTGGAATTTAACAAAACCCCGGCACGGCATGTGCCAAGGAAAACAATAAAACGAGAAGGACTCGACCTGTAATGCCCCGTTTGTGGTGTGCATTCTGAGCGTGTCCTCCTTTGAATCACAAACGACTCTCGGCAACGGATATCTCGGCTCACGCATCGATGAAGAACGTAGCAAAATGCGATACTTGGTGTGAATTGCAGAATCCCGTGAACCATCGAGTTTTTGAACGCAAGTTGCGCCTGAAGCCATCCGGTTGAGGGCACGCCTGCCTGGGCGTCACGCATCGCGTTGCCCCCCATCATACTTCCCTTAAGGGTAGTCGTGGTGATTGGGAGCGGAGATTGGCCTCCCGTACTTGTGGTGCGGTTGGTCAAAATAGGAGTCCCCTTCGGTGGACACACGGCTAGTGGTGGTTGTAAAGACCCTTTTCTTTTGCTGTGTGTTGTGAGCTGTTAGGGAAACCCTCAAATAAGACCCCAATGTGTCGTTCTAGGATGATACtTCGACCGCGACCCCAGGTCAGGCGGGACTACCCGCTGAGTTTAA

>Tuscaloosa59SraALM18

AAGGATCATTGTCGAACCCTGCAAGGCAGAACGACCTGTGAACATGTAAATACAACTCGGGTGATGGGGAGATGGATCTTGGTTCTGATCCTCAACACCTCCTAGCGTGCCTGCATGCTTTCTCTTTTGGGCTATCATGCATGTATTGTTGGAATTTAACAAAACCCCGGCACGGCATGTGCCAAGGAAAACAATAAACGAGAAGGACTCGACCTGTAATGCCCCGTTTGTGGTGTGCATTCTGAGCGTGTCCTCCTTTGAATCACAAACGACTCTCGGCAACGGATATCTCGGCTCACGCATCGATGAAGAACGTAGCAAAATGCGATACTTGGTGTGAATTGCAGAATCCCGTGAACCATCGAGTTTTTGAACGCAAGTTGCGCCCGAAGCCATCCGGTTGAGGGCACGCCTGCCTGGGCGTCACGCATCGCGTCGCCCCCCATCATACTTCCCTTAAGGGTAGTCGTGGTGATTGGGAGCGGAGATTGGCTTCCCGTGCTTGTGGTGCGGTTGGTCAAAATAGGAGTCCCCTTCGGTGGACACACGGCTAGTGGTGGTTGTAAAGACCCTTTTCTTCTGCTGTGTGTTGTGAGCTGTTAGGGAAACCCTCAAATAAGACCCCAATGTGTCGTTCTAGGATGATGCTTCGACTGCGACCCCAGGTCAGGCGGGACTACCCGCTGAGTTTTAAGCATATCAATAAGCGGAGGAA

>BoligeeALM21

CCTTCCGTAGGTGAACCTGCGGAAGGATCATTGTCGAACCCTGCATGGCAGAACGACCTGTGAACATGTAAATACAACTcGGGTGATGGGGAGATGGATCTTGATTCTGATCCTCAACACCTCCTAGCGTGACTGCATGCTTTCTCTTTTGGGCTATCATGCATGTATTGTTGGAATTTAACAAAACCCCGGCACGGCATGTGCCAAGGAAAACAATAAATGAGAAGGACTCGACCTGTTATGCCCCGTTTGTGGTGTGCATTCTGAGCGTGTCCTCCTTTGAATCACAAACGACTCTCGGCAACGGATATCTCGGCTCACGCATCGATGAAGAACGTAGCAAAATGCGATACTTGGTGTGAATTGCAGAATCCCGTGAACCATCGAGTTTTTGAACGCAAGTTGCGCCCGAAGCCATCCGGTTGAGGGCACGCCTGCCTGGGCGTCACGCATCGCGTCGCCCCCCATCATACTTCCCTTAAGGGTAGTCGTGGTGATTGGGAGCGGAGATTGGCTTCCCGTGCTTGTGGTGCGGTTGGTCAAAATAGGAGTCCCCTTCGGTGGACACATGGCTAGTGGTGGTTGTAAAGACCCTTTTCTTCTGCTGTGTGTTGTGAGCTGTTAGGGAAACCCTCAAAAAAGAACCCAATGTATCGTTCTAGGACGATGCTCGACCGCGACCCcAgGTCAGGCGGGACTACCCGCTGAGTTTAA

>Tuscaloosa59SraALM59

AAGGATCATTGTCGAACCCTGCATGGCAGACGACCTGTGaACATGTAAATACAATCGGGTGATGGGGAGATGGATCTTGGTTCTGATCCTCAACACCTCCTAGCGTGCCTGCATGCTTTCTCTTTTGGGCTATCATGCATGTATTGTTGGAATTTAACAAAACCCCGGCACGGCATGTGCCAAGGAAAACAATAAACGAGAAGGACTCGACCTGTAATGCCCCGTTTGTGGTGTGCATTCTGAGCGTGTCCTCCTTTGAATCACAAACGACTCTCGGCAACGGATATCTCGGCTCACGCATCGATGAAGAACGTAGCAAAATGCGATACTTGGTGTGAATTGCAGAATCCCGTGAACCATCGAGTTTTTGAACGCAAGTTGCGCCTGAAGCCATCCGGTTGAGGGCACGCCTGCCTGGGCGTCACGCATCGCGTCGCCCCCCATCATACTTCCCTTAAGGGTAGTCGTGGTGATTGGGAGCGGAGATTGGCTTCCCGTACTTGTGGTGCGGTTGGTCAAAATAGGAGTCCCCTTCGGTGGACACACGGCTAGTGGTGGTTGTAAAGACCCTTTtCTTCTTGCTGTGTGTTGTGAGCTGCTTaGGGAAACCCTCAAAAAAGACCCCAATGTGTCGTTCTAGGATGATACTTCGACTGCGACCCCAGGTCAGGCGGGACTACCCGCTGAGTTTAA

>KnoxvilleTN55

TCGTAaGtGgACCTgGaGaAGGATCattgTTGGACCCTGCAAGGCAGGACGACCTGTGGACACGTAAATACAACTGGGTGATGGGGAGATGGATCTTGGTTCTGATTCTCAACACCTTCTAGCGTGCCTGCATGCTTTCTCTTTTGGGCTATCATGCATGTATTGTTGGAATTTAACAAAACCCCGGCACGGCATGTGCCAAGGAAAACAATAAACGAGAAGGACTTGACCTGTTATGCCCCGTTTGTGGTGTGCATTCTGAGCGTGTCCTTCTTTGAATCACAAACGACTCTCGGCAACGGATATCTTGGCTCACGCATCGATGAAGAACGTAGCAAAATGCGATACTTGGTGTGAATTGCAGAATCCCGTGAACCATCGAGTTTTTGAACGCAAGTTGCGCCCGAAGCCATCCGGTTCAGGGCACGCCTGCCTGGGCGTCACGCATCGCGTCGCCCCCCATCATACTTCCCTTAAGGGTAGTCGTGGTGATTGGGAGCGGAGATTGGCCTCCCGTGCTTGTTGTGCGGTTGGTCCAAATAGGAGTCCCCTTCGGTGGACACACGGCTAGTGGTGGTTGTAAAGACCCTTTTCTTCTGCTGTGTGTTGTGAGCTGCTAGGGAAACCCTCATAAAAGAACCCAATGTATCGTCCTAGGATGATGCTTCGACCGCGACCCCAGGTCAGGCGG

>TceratphorumNCBIconsensus

TGCAGAATCCCGTGAACCATCGAGTTTTTGAACGCAAGTTGCGCCCGAAGCCATCCGGTTCAGGGCACGCCTGCCTGGGCGTCACGCATCGCGTCGCCCCCTCCATCATACTTCCCTTAAAGGTAGTCGTGGTGATTGGGAGCGGAGATTGGCCTCCCGTGCTTGTTGTGCGGTTGGTCTAAATAGGAGTCCCCTTCGGTGGACACACGGCTAGTGGTGGTTGTAAAGACCCTTTTCTTCTGCTGTGTGTTGTGAGCTGCTAGGNAAACCTTCAAAAAAGAACCCAATGTATTGTTCTAGGACGATGCTTCGACCGCGACCCCAGGTCAGGCGGGACTACCCGCTGAGTTTAA

>TbrevicorniculatumS5

TCGACCCTGCACGGCAGAACGACCTGTGAACACGTAAATACAACTGGGTGATGGGGAGATGGATCTTGGTTCTGATTCTCAACACCTCCTAGCGTGCCTGCATGCTTTCTCTTTTGGGCTATCATGCATGTATTGTTGGAATTTAACAAAACCCCGGCACGGCATGTGCCAAGGAAAACAATAAACGAGAAGGACTTGACCTGTTATGCCCCGTTTGTGGTGTGCATTCTGAGCGTGTCCTTCTTTGAAACACAAACGACTCTCGGCAACGGATATCTTGGCTCACGCATCGATGAAGAACGTAGCAAAATGCGATACTTGGTGTGAATTGCAGAATCCCGTGAACCATCGAGTTTTTGAACGCAAGTTGCGCCCGAAGCCATCCGGTTCAGGGCACGCCTGCCTGGGCGTCACGCATCGCGTCGCCCCCCATCATACTTCCCTTAAGGGTAGTCGTGGTGATTGGGAGCGGAGATTGGCCTCCCGTGCTTGTTGTGCGGTTGGTCAAAATAGGAGTCCCCTTCGGTGGACACACGGCTAGTGGTGGTTGTAAAGACCCTTTTCTTCTGGTGTGTGTTGTGAGCTGCTAGGGAAACCGTCATAAAAGACCCCAATGTATCGTTCTAGGATGATGCTTCGACCGCGACCCCAGGTCAGGCGGGA

>TbrevicorniculatumS8

aAGATTCATTGTCGATGCTGCAAGGCAGACGACCTGTGAACACGTAAATACAACTCGGGGTGATGGGGAGATGGATCTTGGTTCTGATCCTCAACACCTCCTAGCGTGCCTGCATGCTTTCTCTTTTGGGCTATCATGCATGTATTGTTGGAATTTAACAAAACCCCGGCaCGGCATGtGCCAAGGAAAACAATAAACGaGAAGGACTTGACCTGTTATGCCCCGTTTGTGGTGTGCATTcTGAGCGTGTCCTTCTTTGAATCACAAACGACTCTCGGCAACGGATATCTTGGCTCACGCATCGATGAAGAACGTAGCAAAATGCGATACTTGGTGTGAATTGCAGAATCCCGTGAACCATCGAGTTTTTGAACGCAAGTTGCGCCCGAAGCCATCCGGTTCAGGGCACGCCTGCCTGGGCGTCACGCATCGCGTCGCCCCCCATCATACTTTCCTTAAGGGTAGTCGTGGTGATTGGGAGCGGAGATTGGCCTTCTGTGCTTGTTGTGCGGTTGGTCAAAATAGGAGTCCCCTTCGGTGGACACACGGCTAGTGGTGGTTGTAAAGACCCTTTTCTTCTGCTGTGTGTTGTGAGCTGCTAGGGAAACCCTCATAAAAGAACCCAATGTATCGTCCTAGGATGATGCTTCGACCGCGACCCCAGGTCAGGCGGAACTACCCGTTGAGTTTAg

>TbrevicornuculatumS6

AAGGATCATTGTTGGAACCTGCCAGGCAGGACGACCTGTGACACGTAAATACAACTCGGGTGATGGGGAGATGGATCTTGGTTCTGATCCTCAACACCTCCTAGCGTGCCTGCATGCTTTCTCTTTTGGGCTATCATGCATGTATTGTTGGAATTTAACAAAACCCCGGCACGGCATGTGCCAAGGAAAACAATAAACGAGAAGGACTTGACCTGTTATGCCCCGTTTGTGGTGTGCATTCTGAGCGTGTCCTTCTTTGAATCACAAACGACTCTCGGCAACGGATATCTTGGCTCACGCATCGATGAAGAACGTAGCAAAATGCGATACTTGGTGTGAATTGCAGAATCCCGTGAACCATCGAGTTTTTGAACGCAAGTTGCGCCCGAAGCCATCCGGTTCAGGGCACGCCTGCCTGGGCGTCACGCATCGCGTCGCCCCCCATCATACTTCCCTTAAGGGTAGTCGTGGTGATTGGGAGCGGAGATTGGCCTCCCGTGCTTGTTGTGCGGTTGGTCAAAATAGGAGTCCCCTTCGGTGGACACACGGCTAGTGGTGGTTGTAAAGACCCTTTTCTTCTGCTGTGTGTTGTGAGCTGCTAGGGAAACCCTCATAAAAGAACCCAATGTATCGTCCTAGGATGATGCTTCGACCGCGACCCCAGGCCAGGCGGAACTACCGGTTGAGTTTAG

>Youngia02

CCTTCCGTAGGTGAACCTGCGGAAGGATCATTGTCGAACCTGCATGGCAGAATGACCCGTGAACATGTAAAAATACTACTGGGTGATGGGGAGACGGTCCTTGGTCTTGATCCTCAATGCCTCCCGACTTGTGTGCATGGTGTCTTGTACAGGCCACCATGTATGTCTCGTTGGACCAATAACAAACCCCGGCACGGAATGTGCCAAGGAAATGAAAAAATAGAGAAGGACTTGTCCTGTTAATGCCCCGTATGCGGTGTGCTTACAGGTCGTGGCCTCCTTGGAATCATAAACGACTCTCGGCAACGGATATCTCGGCTCACGCATCGATGAAGAACGTAGCAAAATGCGATACTTGGTGTGAATTGCAGAATCCCGTGAACCATCGAGTTTTTGAACGCAAGTTGCGCCCAATGCCATTTGGTTGAGGGCACGCCTGCCTGGGCGTCACGCATCGCGTCGCCCCCCTTCCATACTTCCTTAACCGGTACATCTGGTGCTGGGGGCGGAGATTGGCCTCCCATGCCTGTGGTGTGGCTGGCCTAAAGAGTTGTCCCATTTGGTGGACACACGGTTAGTGGTGGTTGTATAGACCCTCGTCTTGTCCCGTGTGTCGTTAGCCTCTAGGGACATATTGAAAAGACCCTACTGTATTGTCTCTAGATGANACTTCGACTGCGACCC

>Youngia01

AAGGATCATTGTCGAACCCTGCAAGGCaGAACGACCTGTGAACACGTAAATACAACTGGGTGATGGGGAGATGGATCTTGGTTCTGATCCTCAACRCCTCCTAGCGTGCCTGCATGCTTTCTCTTTTGGGCTATCATGCATGTATTGTTGGAATTTAACAAAACCCCGGCACGGCATGTGCCAAGGAAAACAATAAACGAGAAGGACTCGACCTGTTATGCCCCGTTTGTGGTGTGCATTCTGAGCGTGTCCTCCTTTGAATCACAAACGACTCTCGGCAACGGATATCTCGGCTCACGCATCGATGAAGAACGTAGCAAAATGCGATACTTGGTGTGAATTGCAGAATCCCGTGAACCATCGAGTTTTTGAACGCAAGTTGCGCCCGAAGCCATCCGGTTCAGGGCACGCCTGCCTGGGCGTCACGCATCGCGTCGCCCCCCATCATACTTCCCTTAAGGGTAGTCGTGGTGATTGGGAGCGGAGATTGGCTTCCCGTGCTTGTTGTGCGGTTGGTCAAAATAGGAGTCCCCTTCGGTGGACACACGGCTAGTGGTGGTTGTAAAGACCCTTTTCTTCTGCTGTGTGTTGTGAGCTGCTAGGGAAACCCTCAAAAAAGAACCCAATGTATCGTTCTAGGACGATGCTTCGACCGCGACCCCAGGTCAGGCGGGACTACCCGCTGAGTTTAAG_KAGGATCATTGTCGAACCTGCATGGCAGAATGACCCGTgAACATGTAAAAATACTACTGGGTGATGGGGAGACGGTCCTTGGTCTTGATCCTCAATGCCTCCCGACTTGTGTGCATGGTGTCTTGTACAGGCCACCATGTATGTCTCGTTGGACCAATAACAAACCCCGGCACGGAATGTGCCAAGGAAATGAAAAAATAGAGAAGGACTTGTCCTGTTAATGCCCCGTATGCGGTGTGCTTACAGGTCGTGGCCTCCTTGGAATCATAAACGACTCTCGGCAACGGATATCTCGGCTCACGCATCGATGAAGAACGTAGCAAAATGCGATACTTGGTGTGAATTGCAGAATCCCGTGAACCATCGAGTTTTTGAACGCAAGTTGCGCCCAATGCCATTTGGTTGAGGGCACGCCTGCCTGGGCGTCACGCATCGCGTCGCCCCCCTTCCATACTTCCTTAACCGGTACATCTGGTGCTGGGGGCGGAGATTGGCCTCCCATGCCTGTGGTGTGGCTGGCCTAAAGAGTTGTCCCATTTGGTGGACACACGGTTAGTGGTGGTTGTATAGACCCTCGTCTTGTCCCGTGTGTCGTTAGCCTCTAGGGACATATTGAAAAGACCCTACTGTATTGTCTCTAGATGATACTTCGACTGCGACCCCAGGTCAGGCGGGACTACCCGCTGAGTTTAA

>Youngia03

AAGGATCATTGTCGAACCTGCATGGCAGAATGACCCGTGAACATGTAAAAATACTACTGGGTGATGGGGAGACGGTCCTTGGTCTTGATCCTCAATGCCTCCCGACTTGTGTGCATGGTGTCTTGTACAGGCCACCATGTATGTCTCGTTGGACCAATAACAAACCCCGGCACGGAATGTGCCAAGGAAATGAAAAAATAGAGAAGGACTTGTCCTGTTAATGCCCCGTATGCGGTGTGCTTACAGGTCGTGGCCTCCTTGGAATCATAAACGACTCTCGGCAACGGATATCTCGGCTCACGCATCGATGAAGAACGTAGCAAAATGCGATACTTGGTGTGAATTGCAGAATCCCGTGAACCATCGAGTTTTTGAACGCAAGTTGCGCCCAATGCCATTTGGTTGAGGGCACGCCTGCCTGGGCGTCACGCATCGCGTCGCCCCCCTTCCATACTTCCTTAACCGGTACATCTGGTGCTGGGGGCGGAGATTGGCCTCCCATGCCTGTGGTGTGGCTGGCCTAAAGAGTTGTCCCATTTGGTGGACACACGGTTAGTGGTGGTTGTATAGACCCTCGTCTTGTCCCGTGTGTCGTTAGCCTCTAGGGACATATTGAAAAGACCCTACTGTATTGTCTCTAGATGATACTtCGACTGCGACCCCAGGTCAGGCGGGACTACCCGCTGAGTTTAA

>Youngia04

AAGGATCATTGTCGAACCTGCATGGCAGaATGACCCGTGAACATGTaAAAATACTACTGGGTGATGGGGAGACGGTCCTTGGTCTTGATCCTCAATGCCTCCCGACTTGTGTGCATGGTGTCTTGTACAGGCCACCATGTATGTCTCGTTGGACCAATAACAAACCCCGGCACGGAATGTGCCAAGGAAATGAAAAAATAGAGAAGGACTTGTCCTGTTAATGCCCCGTATGCGGTGTGCTTACAGGTCGTGGCCTCCTTGGAATCATAAACGACTCTCGGCAACGGATATCTCGGCTCACGCATCGATGAAGAACGTAGCAAAATGCGATACTTGGTGTGAATTGCAGAATCCCGTGAACCATCGAGTTTTTGAACGCAAGTTGCGCCCAATGCCATTTGGTTGAGGGCACGCCTGCCTGGGCGTCACGCATCGCGTCGCCCCCCTTCCATACTTCCTTAACCGGTACATCTGGTGCTGGGGGCGGAGATTGGCCTCCCATGCCTGTGGTGTGGCTGGCCTAAAGAGTTGTCCCATTTGGTGGACACACGGTTAGTGGTGGTTGTATAGACCCTCGTCTTGTCCCGTGTGTCGTTAGCCTCTAGGGACATATTGAAAAGACCCTACTGTATTGTCTCTAGATGATACTTCGACTGCGACCCCAGGTCAGGCGGGACTACCCGCTGAGTTTAA

>Youngia05

AAGGATCATTGTCGAACCTGCATGGCAGAATGACCCGTGAACATGTAAAAATACTACTGGGTGATGGGGAGACGGTCCTTGGTCTTGATCCTCAATGCCTCCCGACTTGTGTGCATGGTGTCTTGTACAGGCCACCATGTATGTCTCGTTGGACCAATAACAAACCCCGGCACGGAATGTGCCAAGGAAATGAAAAAATAGAGAAGGACTTGTCCTGTTAATGCCCCGTATGCGGTGTGCTTACAGGTCGTGGCCTCCTTGGAATCATAAACGACTCTCGGCAACGGATATCTCGGCTCACGCATCGATGAAGAACGTAGCAAAATGCGATACTTGGTGTGAATTGCAGAATCCCGTGAACCATCGAGTTTTTGAACGCAAGTTGCGCCCAATGCCATTTGGTTGAGGGCACGCCTGCCTGGGCGTCACGCATCGCGTCGCCCCCCTTCCATACTTCCTTAACCGGTACATCTGGTGCTGGGGGCGGAGATTGGCCTCCCATGCCTGTGGTGTGGCTGGCCTAAAGAGTTGTCCCATTTGGTGGACACACGGTTAGTGGTGGTTGTATAGACCCTCGTCTTGTCCCGTGTGTCGTTAGCCTCTAGGGACATATTGAAAAGACCCTACTGTATTGTCTCTAGATGATACTTCGACTGCGACCCCAGGTCAGGCGGGACTACCCGCTGAGTTTAA

>PinedaleALM37

CAGACGACCTGTGACATGTAAATACAATTCGGGTGATGGGGAGaATGGATCTTGGTTCTGATCCTCAACACCTCCTAGCGTGCCTGCATGCTTTtCTCtTTTTGGGCTATCATGCATGTATTGTTGGAATTTAACAAAACCCCGGCACGGCATGTGCCAAGGAAAACAAAAAACGAGAAGGACTCGACCTGTAATGCCCCGTTTGTGGTGTGCATTCTGAGCGTGTCCTCCTTTGAATCACAAACGACTCTCGGCAACGGATATCTCGGCTCACGCATCGATGAAGAACGTAGCAAAATGCGATACTTGGTGTGAATTGCAGAATCCCGTGAACCATCGAGTTTTTGAACGCAAGTTGCGCCTGAAGCCATCCGGTTGAGGGCACGCCTGCCTGGGCGTCACGCATCGCGTTGCCCCCCATCATACTTCCCTTAAGGGTAGTCGTGGTGATTGGGAGCGGAGATTGGCCTCCCGTACTTGTGGTGCGGTTGGTCAAAATAGGAGTCCCCTTCGGTGGACACACGGCTAGTGGTGGTTGTAAAGACCCTTTTCTTTTGCTGTGTGTTGTGAGCTGTTTGGGAAACCCTCAAAAAAGACCCCAATGTGTCGTTCTAGGATGATACTtCGACCGCGaCCCCAGGTCAGGCGGGACTACCCGCTGAGTTTAA

>MaryvilleTNT8

AAGGATCATTGTCGAACCATGCATGGCaGAACGACCTGTGAACATGTAAATACAATCGGGTGATGGGGAAATGGATCTTGGTTCTGATCCTCAACACCTCCTAGCGTGCCTGCATGCTTTCTCTTTTGGGCTATCATGCATGTATTGTTGGAATTTAACAAAACCCCGGCACGGCATGTGCCAAGGAAAACAAAAAACGAGAAGGACTCGACCTGTAATGCCCCGTTTGTGGTGTGCATTCTGAGCGTGTCCTCCTTTGAATCACAAACGACTCTCGGCAACGGATATCTCGGCTCACGCATCGATGAAGAACGTAGCAAAATGCGATACTTGGTGTGAATTGCAGAATCCCGTGAACCATCGAGTTTTTGAACGCAAGTTGCGCCTGAAGCCATCCGGTTGAGGGCACGCCTGCCTGGGCGTCACGCATCGCGTTGCCCCCCATCATACTTCCCTTAAGGGTAGTCGTGGTGATTGGGAGCGGAGATTGGCCTCCCGTACTTGTGGTGCGGTTGGTCAAAATAGGAGTCCCCTTCGGTGGACACACGGCTAGTGGTGGTTGTAAAGACCCTTTTCTTTTGCTGTGTGTTGTGAGCTGTTTGGGAAACCCTCAAAAAAGACCTACaCTGTATCATaTCCTCCGCTTATTGATATGCATATCAATAAGCGGAgGtaT

>MaryvilleTNT3

AAGGATCATTGTTAGAACCATGCTCAGGCAGAATGACTTCGTGAAcACGtAATTAcTATTGGGAGATGGGGAgCTGCATCTTGGtTCTGATCCTCAACACCTCCTAGCGTGCCTGCATGCTTTCTCTTTTGGGCTATCATGCATGTATTGTTGGAATTTAACAAAACCCCGGCACGGCATGTGCCAAGGAAAACAATAAACGAGAAGGAcTCGACCTGTTATGCCCCGtTTGTGGTGTGCATTCTGAGCGTGTCCTCCTTTGAATCACAAACGACTCTCGGCAACGGATATCTCGGCTCACGCATCGATGAAGAACGTAGCAAAATGCGATACTTGGTGTGAATTGCAGAATCCCGTGAACCATCGAGTTTTTGAACGCAAGTTGCGCCCGAAGCCATCCGGTTCAGGGCACGCCTGCCTGGGCGTCACGCATCGCGTCGCCCCCCATCATACTTCCCTTAAGGGTAGTCGTGGTGATTGGGAGCGGAGATTGGCTTCCCGTGCTTGTTGTGCGGTTGGTCAAAATAGGAGTCCCCTTTGGTGGACACACGGCTAGTGGTGGTTGTAAAGACCCTTTTATTTTGTTGTGctGTTGTGAGTTGCTAGGGAAACCCTCAAAAAAGAAACCAACGGATCAaTTCTCCGCTTATTGATATGCATTCaTAGCGAga

>KnoxvilleTNK1

AGGATCATTGTCGAACCCTGCAAGGCAGAACGACCTGTGAACACGTAAATACAACTGGGTGATGGGGAGATGGATCTTGGTTCTGATCCTCAACACCTTCTAGCGTGCCTGCATGCTTTCTCTTTTGGGCTATCATGCATGTATTGTTGGAATTTAACAAAACCCCGGCACGGCATGTGCCAAGGAAAACAATAAACGAGAAGGACTCGACCTGTTATGCCCCGTTTGTGGTGTGCATTCTGAGCGTGTCCTCCCTTTGAATCACAAACGACTCTCGGCAACGGGATATCTCGGCTCACGCATCGATGAAGAAACGTAGCAAAAATGCGATACTTGGGTGTGAAATTGCAGAATCCCGTGAACCATCGAGTTTTTGAACGCAAGTTGCGCCCGAAGCCATCCGGTTCAGGGGCCACGCCTTGCCTGGGCGTCACGCATCGCCGTCGCCCCCCCCAATCATACTTTCCCTTTAAGGGTAG

>Lactuca_canadensisa

CCTTTTCCGTAGGGTGAACCTGCGGAAGGATCATTGTCGAaCCCTGCAGACAGTACGACCTGTGAACATGTTAACACAACGGGGCGACAGGGAAACGGGCCTTGGTCCTGATCCCCCAACCCCTCCCGACGTGCGTTTGTGATGCCTTCTATTGGGCATCATGGATCCCGTCGGACCATAACAAACCCCGGCACGGCATGTGCCAAGGAAAACAAAAATGAGAAGGACACGTCTTGTAATGCCCCGTTTGCGGTGTGCATGCGTTTCGTGGCCTCCTAGGAAAACTACAAACGACTCTCGGCAACGGATATCTCGGCTCACGCATCGATGAAGAACGTAGCAAAATGCGATACTTGGTGTGAATTGCAGAATCCCGTGAACCATCGAGTTTTTGAACGCAAGTTGCGCCTGAAGCCATCCGGCCGAAGGCACGCCTGCCTGGGCGTCACGCATCGCGTCGCTCCCCACCATACTCCTCCAATGGGTTGTGATGGTGTTAGGGGCGGATAATGGCCTCCCGTGCTTGTGTTTCGGTTGGCCTAAAAAGGAGTTCCCTTCGGCGGACACACAACTAGTGGTGGTTGAACATACCCTTGCCTTGTTGTGCGTCGTGAGCTGTGAGGGAAGCCCTCATCTAAACGACCCCATTGTATCGTCTTTGGACGGTGCTTCGACCGCGACCCCAGGTCAGGCGGGACTACCCGCTGAGTTTAAGCATATCAATAAGCCGGAGGAAAGG

>Hypochaeris02

CCTTCCGTAGGGTGACCNTGCGNNAAGGATCATTGTCGAACCCNTGCAAAGGCAGAACGACCCGTGAACATGTAAATACAACCGGGCGATGGGGAGACTGGCATTGGTCCGTATCCTCATCCTCTCCTGGCNTGCGTTTGTGGTGCCTCGTATGGGGTGCCATAGATGACATGCTGGACCATAACAAAAACCCGGCACGGCATGTGCCAAGGAAAACAAAAATTGAGATGGACTCGTCTTGTGTTGCCCCGTTTGCGGTGTGCATGCAGTTCGTGGCCTCTTTGTAATTACAAACGACTCTCGGCAACGGATATCTCGGCTCACGCATCGATGAAGAACGTAGCAAAATGCGATACTTGGTGTGAATTGCAGAATCCCGTGAACCATCGAGTTTTTGAACGCAAGTTGCGCCCGAAGCCATCCGGTCGAGGGCACGCCTGCCTGGGCGTCACGCATCGCGTCGCCCCCACCATATCTACTCTCGGTGATGGGAGCGGAGATTGGTCTCCCGTACTTGGTTGCGGTTGGCCTAAAAAGGAGTCCCCTACAGCGGACACACGACTAGTGGTGGTTGAATAGACCCTCGTTTTTATCGTGTGTCGTGAGTTGTTTGGGAAGCCCTCAACTAAGACCCGATTGTATCGTTTTAG

>Hypochaeris01

AAGGATCATTGTCGAACCCTGCAAGGCAGAACGACCCGTGAACATGTAAATACAACCGGGCGATGGGGAGACTGGCATTGGTCCGTATCCTCATCCTCTCCTGGCGTGCGTTTGTGGTGCCTCGTATGGGGTGCCATAGATTACATGCTGGACCATAACAAAAACCCGGCACGGCATGTGCCAAGGAAAACAAAAATTGAGATGGACTCGTCTTGTGTTGCCCCGTTCGCGGTGTGCATGCAGTTCGTGGCCTCTTTGTAATTACCAACGACTCTCGGCAACGGATATCTCGGCTCACGCATCGATGAAGAACGTAGCAAAATGCGATACTTGGTGTGAATTGCAGAATCCCGTGAACCATCGAGTTTTTGAACGCAAGTTGCGCCCGAAGCCATCCGGTCGAGGGCACGCCTGCCTGGGCGTCACGCATCGCGTCGCCCCCATCATATCTACTCTTGGTGATGGGAGCGGAGATTGGTCTTCCGTACTTGGTTGCGGTTGGCCTAAAAAGGAGTCCCCTACAGCGGACACACGACTAGTGGTGGTTGAATAGACCCTCGTTTTTATCGTGTGTCGTGAGCTGTTTGGGAAGCCCTCAACTAAGACCCCGATTGTATCGTTTTAGGACGGTGCTtCGACCGCGACCCCAGgTCAGGCGGGACTACCCGCTGAGTTTAA

>Hypochaeris58

AAGGATCATTGTCGAACCCTGCAAGGCAGAACGACCCGTGGACATGTTAATACAACCGGGCGATGGGGAGACTGGCATTGGTCCGTATCCTCATCCTCTCCTGGCGTGCGTTTGTGGTGCCTCGTATGGGGTGCCATAGATTACATGCTGGACCATAACAAAAACCCGGCACGGCATGTGCCAAGGAAAACAAAAATTGAGATGGACTCGTCTTGTGTTGCCCCGTTCGCGGTGTGCATGCAGTTCGTGGCCTCTTTGTAATTACAAACGACTCTCGGCAACGGATATCTCGGCTCACGCATCGATGAAGAACGTAGCAAAATGCGATACTTGGTGTGAATTGCAGAATCCCGTGAACCATCGAGTTTTTGAACGCAAGTTGCGCCCGAAGCCATCCGGTCGAGGGCACGCCTGCCTGGGCGTCACGCATCGCGTCGCCCCCATCATATCTACTCTTGGTGATGGGAGCGGAGATTGGTCTCCCGTACTTGGTTGCGGTTGGCCTAAAAAGGAGTCCCCTACAGCGGACACACGACTAGTGGTGGGTGAATAGACCCTCGTTTTTATCGTGTGTCGTGAGCTGTTTGGGAAGCCCTCAACTAAGACCCCGATTGTATCGTTTTAGGACGGTGCtTCGACCGCGACCCCAGGTCAGGGGGGACTACCCGCTGAGTTTAA

>Hypochaeris17

AAGGATCATTGTCGAACCCTGCAAGGCAGAACGACCCGTGAACATGTAAATACAACCGGGCGATGGGGAGACTGGCATTGGTCCGTATCCTCATCCTCTCCTGGCGTGCGTTTGTGGTGCCTCGTATGGGGTGCCATAGATTACATGCTGGACCATAACAAAAACCCGGCACGGCATGTGCCAAGGAAAACAAAAATTGAGATGGACTCGTCTTGTGTTGCCCCGTTCGCGGTGTGCATGCAGTTCGTGGCCTCTTTGTAATTACAAACGACTCTCGGCAACGGATATCTCGGCTCACGCATCGATGAAGAACGTAGCAAAATGCGATACTTGGTGTGAATTGCAGAATCCCGTGAACCATCGAGTTTTTGAACGCAAGTTGCGCCCGAAGCCATCCGGTCGAGGGCACGCCTGCCTGGGCGTCACGCATCGCGTCGCCCCCATCATATCTACTCTTGGTGATGGGAGCGGAGATTGGTCTCCCGTACTTGGTTGCGGTTGGCCTAAAAAGGAGTCCCCTACAGCGGACACACGACTAGTGGTGGTTGAATAGACCCTCGTTTTTATCGTGTGTCGTGAGCTGTTTGGGAAGCCCTCAACTAAGACCCCGATTGTATCGTTTTAGGACGGTGCTTCGACCGCGACCCCAGGTCAGGCGGGACTACCCGCTGAGTTTAAGCATATCAATAAGCNGGAGGAA

>Hypochaeris14

AAGGATCATTGTCGAACCCTGCAAGGCAGAACGACCCGTGAACATGTAAATACAACCGGGCGATGGGGAGACTGGCATTGGTCCGTATCCTCATCCTTTCCTGGCGTGCGTTTGTGGTGCCTTGTATGGGGTGCCATAGATTACATGCTGGACCATAACAAAAACCCGGCACGGCATGTGCCAAGGAAAACAAAAATTGAGATGGACTTGTCTTGTGTTGCCCCGTTCGCGGTGTGCATGCAGTTCGTGGCCTCTTTGTAATTACAAACGACTCTCGGCAACGGATATCTCGGCTCACGCATCGATGAAGAACGTAGCAAAATGCGATACTTGGTGTGAATTGCAGAATCCCGTGAACCATCGAGTTTTTGAACGCAAGTTGCGCCCGAAGCCATCCGGTCGAGGGCACGCCTGCCTGGGCGTCACGCATCGCGTCGCCCCCATCATATCTACTCTTGGTGATGGGAGCGGAGATTGGTCTCCCGTACTTGGTTGCGGTTGGCCTAAAAAGGAGTCCCCTACAGCGGACACACGACTAGTGGTGGTTGAATAGACCCTCGTTTTTATCGTGTGTCGTGAGCTGTTTGGGAAGCCCTCAACTAAGACCCCGATTGTATCGTTTTAGGACGGTGCTTCGACCGCGACCCCAGGcCAGGCGGGACTACCCGCTGAGTTTAAGCATATCAATAAGCGGAGGAA

>Hypochaeris46

AAGGATCATTGTCGAACCCTGCAAAGGCAGAACGACCCGTGAACATGTAGATACAACTGGGTGATGGGGAGACTGGCATTGGTCCTTATCCTCATCCTCTACTGGCATGCGTTTATGGTGCCTTGTATGGGGTGCCATAGATGACATGCTGGACCATAACAAAAACCCGGCACGGCATGTGCCAAGGAAAACAAAAATTGAGATGGACTCGTCTTGTGTTGCCCCGTTCGCGGTGTGCATGCAGTTCGTGGCCTCTTTGTAATTACAAACGACTCTCGGCAACGGATATCTCGGCTCACGCATCGATGAAGAACGTAGCAAAATGCGATACTTGGTGTGAATTGCAGAATCCCGTGAACCATCGAGTTTTTGAACGCAAGTTGCGCCCGAAGCCATCCGGTCGAGGGCACGTCTGCCTGGGCGTCACGCATCGCGTCGCCCCCACCATATCTACTCTTGGTGATGGGAGCGGAGATTGGTCTCCCGTACTTGGTTGCGGTTGGCCTAAAAAGGAGTCCCCTACAGCGGACACACGACTAGTGGTGGTTGAATAGACCCTCGTTTTTATCGTGTGTCGTGAGCTGTTTGGGAAGCCCTCAACTAAGACCCGATTGTATCGTTTTGGAACGGTGCTTCGACCGCGACCCCAGGTCAGGCGGGACTACCCGCTGAGTTTAA

>Hypochaeris49

AAGGATCATTGTCGAACCCTGCAAAGGCAGAACGACCCGTGAACATGTAGATACAACTGGGTGATGGGGAGACTGGCATTGGTCCTTATCCTCATCCTCTACTGGCATGCGTTTATGGTGCCTTGTATGGGGTGCCATAGATGACATGCTGGACCATAACAAAAACCCGGCACGGCATGTGCCAAGGAAAACAAAAATTGAGATGGACTCGTCTTGTGTTGCCCCGTTCGCGGTGTGCATGCAGTTCGTGGCCTCTTTGTAATTACAAACGACTCTCGGCAACGGATATCTCGGCTCACGCATCGATGAAGAACGTAGCAAAATGCGATACTTGGTGTGAATTGCAGAATCCCGTGAACCATCGAGTTTTTGAACGCAAGTTGCGCCCGAAGCCATCCGGTCGAGGGCACGTCTGCCTGGGCGTCACGCATCGCGTCGCCCCCACCATATCTACTCTTGGTGATGGGAGCGGAGATTGGTCTCCCGTACTTGGTTGCGGTTGGCCTAAAAAGGAGTCCCCTACAGCGGACACACGACTAGTGGTGGTTGAATAGACCCTCGTTTTTATCGTGTGTCGTGAGCTGTTTGGGAAGCCCTCAACTAAGACCCGATTGTATCGTTTTGGAACGGTGCTTCGACCGCGACCCCAGGTCAGGCGGGACTACCCGCTGAGTTTAA

>Hypochaeris51

AAGGATCATTGTCGAACCCTGCAAAGGCAGAACGACCCGTGAACATGTAAATACAACCGGGCGATGGGGAGACTGGCATTGGTCCGTATCCTCATCCTCTCCTGGCRTGCGTTTGTGGTGCCTCGTATGGGGTGCCATAGATGACATGCTGGACCATAACAAAAACCCGGCACGGCATGTGCCAAGGAAAACAAAAATTGAGATGGACTCGTCTTGTGTTGCCCCGTTTGCGGTGTGCATGCAGTTCGTGGCCTCTTTGTAATTACAAACGACTCTCGGCAACGGATATCTCGGCTCACGCATCGATGAAGAACGTAGCAAAATGCGATACTTGGTGTGAATTGCAGAATCCCGTGAACCATCGAGTTTTTGAACGCAAGTTGCGCCCGAAGCCATCCGGTCGAGGGCACGCCTGCCTGGGCGTCACGCATCGCGTCGCCCCCACCATATCTACTCTCGGTGATGGGAGCGGAGATTGGTCTCCCGTACTTGGTTGCGGTTGGCCTAAAAAGGAGTCCCCTACAGCGGACACACGACTAGTGGTGGTTGAATAGACCCTCGTTTTTATCGTGTGTCGTGAGTTGTTTGGGAAGCCCTCAACTAAGACCCGATTGTATCGTTTTAGAACGGTGCtTCGAcCGCgaAcccCagggtatAggcggaattaccgttaagtt

>Hypochaeris10

AAGGATCATTGTCGAACCCTGCATGGCAGAACGACCAGTTAACACGTAAATACAACTGGGTGATGGGGAGATGGGCCTTGGCTCTTTTCCTTATCCCCTCTCGGTGTGTGTTTGTGATGCCTCTTTTGGGGCGCCACTGACGTCATGCTGAACCTTAACAAACCCCGGCACGGCATGTGCCAAGGAAAACAAAATCGAGAAGGACGCGTCTTGTGTTGCCCCGTTCGCGGTGTGCATGCGGGCCGTGGCCTTTTTATAATTACAAACGACTCTCGGCAACGGATATCTCGGCTCACGCATCGATGAAGAACGTAGCAAAATGCGATACTTGGTGTGAATTGCAGAATCCCGTGAACCATCGAGTTTTTGAACGCAAGTTGCGCCCGAAGCCATCCGGCCGAGGGCACGCCTGCCTGGGCGTCACGCATCGCGTCGCCCCCACCAAATTTCCAAATCTGGTTGGGGGCGGAGATTGGCCTCCCGTACCTGTTGTGGTTGGCCTAAAAAGGAGTCCCCTTCGGTGGACACACGACTAGTGGTGGTTGAACAGACCCTCGTCTTTATTGTGTGTCATGAGCTGCTAGGGAGCCCTCATCAAAGACCCTTTGTATCGTTTTCGGACGGTGCTTCGACCGCGACCCCAGGTCAGACGGGACTACCCGCTGAGTTTAAGCATATCAATAAAGCGGAGGAA

>Pyrrhopappus_carolinianus

TTCCGTAGGTGAACCTGCGGAAGGATCATTGTCGAACCCTGCaAAGCAGAACGACCCGTGAACATGTAAACACAACTGGGAGTCGGAAACATTGGCTCCGGCCTTTGTCTTCGAATCCCTGCCGGCATACGTTTGTGGCATCCCGTTCGGGACGACACGAAAGTCATGCCGGCACCATAACAAACCCCGGCACGGAATGTGCCAAGGAAAACGAAATATGAGAAGGGCATGTCCATTATCGCCCCGTACGCGGTGTGCGTGTTGGTGTGACCTCCTTGAAATCACAAACGACTCTCGGCAACGGATATCTCGGCTCACGCATCGATGAAGAACGTAGCAAAATGCGATACTTGGTGTGAATTGCAGAATCCCGTGAACCATCGAGTTTTTGAACGCAAGTTGCGCCCAAAGCCATCCGGTCGAGGGCACGCCTGCCTGGGCGTCACGCATCGCGTCGCCCCCAACCATCCATCCCACGGGATGCCTGGCATCGGGGCGGAGATTGGCCTCCCGTGCTTTTGGTGCGGTTGGCCTAAACTGGAGTCACCTTCGGTGGACGCACGACTAGTGGTGGTTGAACAGACCCTCGTCTTATGTTGTGCGTTGTAAGCTGTGAGGTGGGCCCTTGATGAAGACCCCTATGTGTCGTCATGTGACGATGCTTCGACCGCGACCCCAGGTCAGGCGGGACtACCCGCTGAGTTTAAGCATATCAATAAGCGGAGGAA

>Krigia01

AAGGATCATTGTCGAACCCTGCAAAGCAGaACGACCCGCGaACTTGTACCCATAATCGGGAGTCAGGGATATTGGCTCTGTCCTTTGTCCCTGACACCCTGTCGGCATATGTTTGTGGTGCCCCGTTAGGGATGCCACGGATGTCATGTCGGCGCATTAACAAACCCCGGCACGGAATGTGCCAAGGAAAACAAAAAACTGAGAAGGACGCGTCCAATTTTGCCCCGTTTGCGGTGTGCTTGTTGGCGTGGCCTCCTTGAAATCACAAACGACTCTCGGCAACGGATATCTCGGCTCACGCATCGATGAAGAACGTAGCAAAATGCGATACTTGGTGTGAATTGCAGAATCCCGTGAACCATCGAGTTTTTGAACGCAAGTTGCGCCCAAAGCCATCCGGCCGAGGGCACGCCTGCCTGGGCGTCACGCATCGCGTCGCCCCCAACCATGCATCCTTATGGGATGCTTGGCATCGGGGCGGAGATTGGCCTCCCGTGCCTTTGGTGTGGTTGGCCTAAATCGGAGTCACCTTCGGTGGACGCACGACTAGTGGTGGTTGAAAAGACCCTCGTCCTGTGTTGTGCGTCGTAAGCTGTGAGGGAGGCCCTTCATGAAGACCCCAATGTGTCGTCTTGCGACGATGCTTCGACCGCGACCCCAGGTCAGGCGGGACTACCCGCTGAGTTTAA

>Krigia02

AAGGATCATTGTCGAACCCTGCAAAGCAGAACGACCCGCGAACTTGTACCCATAATCGGGAGTCAGGGATATTGGCTCTGTCCTTTGTCCCTGACACCCTGTCGGCATATGTTTGTGGTGCCCCGTTAGGGATGCCACGGATGTCATGTCGGCGCATTAACAAACCCCGGCACGGAATGTGCCAAGGAAAACAAAAAACTGAGAAGGACGCGTCCAATTTTGCCCCGTTTGCGGTGTGCTTGTTGGCGTGGCCTCCTTGAAATCACAAACGACTCTCGGCAACGGATATCTCGGCTCACGCATCGATGAAGAACGTAGCAAAATGCGATACTTGGTGTGAATTGCAGAATCCCGTGAACCATCGAGTTTTTGAACGCAAGTTGCGCCCAAAGCCATCCGGCCGAGGGCACGCCTGCCTGGGCGTCACGCATCGCGTCGCCCCCAACCATGCATCCTTATGGGATGCTTGGCATCGGGGCGGAGATTGGCCTCCCGTGCCTTTGGTGTGGTTGGCCTAAATCGGAGTCACCTTCGGTGGACGCACGACTAGTGGTGGTTGAAAAGACCCTCGTCCTGTGTTGTGCGTCGTAAGCTGTGAGGGAGGCCCTTCATGAAGACCCCAATGTGTCGTCTTGCGACGATGCTTCGACCGCGACCCCAGGTCAGGCGGGACTACCCGCTGAGTTTAA

>Krigia03

AAGGATCATTGTCGAACCCTGCAAAGCAGAACGACCCGCGAACTTGTACCCATAATCGGGAGTCAGGGATATTGGCTCTGTCCTTTGTCCCTGACACCCTGTCGGCATATGTTTGTGGTGCCCCGTTAGGGATGCCACGGATGTCATGTCGGCGCATTAACAAACCCCGGCACGGAATGTGCCAAGGAAAACAAAAAACTGAGAAGGACGCGTCCAATTTTGCCCCGTTTGCGGTGTGCTTGTTGGCGTGGCCTCCTTGAAATCACAAACGACTCTCGGCAACGGATATCTCGGCTCACGCATCGATGAAGAACGTAGCAAAATGCGATACTTGGTGTGAATTGCAGAATCCCGTGAACCATCGAGTTTTTGAACGCAAGTTGCGCCCAAAGCCATCCGGCCGAGGGCACGCCTGCCTGGGCGTCACGCATCGCGTCGCCCCCAACCATGCATCCTTATGGGATGCTTGGCATCGGGGCGGAGATTGGCCTCCCGTGCCTTTGGTGTGGTTGGCCTAAATCGGAGTCACCTTCGGTGGACGCACGACTAGTGGTGGTTGAAAAGACCCTCGTCCTGTGTTGTGCGTCGTAAGCTGTGAGGGAGGCCCTTCATGAAGACCCCAATGTGTCGTCTTGCGACGATGCTTCGACCGCGACCCCAGGTCAGGCGGGACTACCCGCTGAGTTTAA

>Erigeron02

AAGGATCATTGTCGAAGCCTGCAAAGCAGAACGACCCGCGAACATGTTAAAACAATCATGCCAGGATGTATTGAGCATCCGTTTGATCGTCCTGGCATACCGTTGATGTGCCTGCCTCGTTGGCCCAGTGGGTCATCTTGGTGGTCGCTTTGACGTAACAAAACCCAGGCACGGGATGTGCCAAGGAACTTTAAACTGAAGAATTGCCCATCCCAATGAAGTCCCGTTCGCGGTGTGCTCATGGGGTGTGGCATCTTTGTAATCACAAACGACTCTCGGCAACGGATATCTCGGCTCACGCATCGATGAAGAACGTAGCAAAATGCGATACTTGGTGTGAATTGCAGAATCCCGTGAACCATCGAGTTTTTGAACGCAAGTTGCGCCCGAAGCCATTCGGCTGAGGGCACGTCTGCCTGGGCGTCACGCATCGCGTCGCTCCCCCACCATTTCCTTTTGGATTGTTGGCTGGGAGCGGATATTGGCCTCCCGTTTTAACCGAGTGGTTGGCCAAAATAAAAGCACCTCTTGACGGGCGCAAGACTATTGGTGACAAAACCATGAATTTCGTTGCGTGTCTCGTCAAAAGGTTGCTTGTTATCGACCCAACGCGTTGTCTTTTGATGACGCTTCGACCGCGACCCCAGGTCAGGCGGGACTACCCGCTGAGTTTAAGCATATCAATAAGCGGAGGAAGCGTC

>Erigeron01

AAGGATCATTGTCGAAGCCTGCAAAGCAGAACGACCCGCGAACATGTTAAAACAACCATGCCAGGATGTGTCGAGCATTCGTTCGATCGTTCTGGCATACCGTTGATGTGCCTGCCTAGTTGGCCCTCTGGGTCATCTTGGTGGTCGCATTGACGTAACAAAACCCAGGCACGGGATGTGCCAAGGAACTTAAAATTGAAGAATTGCCTGTCCCATAGTCCCGTTCGCGGTGTGCTCATGGGGTCTGGCATCTTTGTAATCACAAACGACTCTCGGCAACGGATATCTCGGCTCACGCATCGATGAAGAACGTAGCAAAATGCGATACTTGGTGTGAATTGCAGAATCCCGTGAACCATCGAGTTTTTGAACGCAAGTTGCGCCCGAAGCCATTCGGTTGAGGGCACGTCTGCCTGGGCGTCACGCATCGCGTCGCTCCCCCAACATTTCCTTTGGGATGCTTGGTTGGGAGCGGATATTGGTCTCCCGTTTTCACCGAGCGGTTGGCCGAAATAAAAGCACCTCTTGACGGGCGCAAGACTATTGGTGACAAAACCATGAATTTTGTTGCGTGTCTCGTTAAAAGGATGCTTCTTATAGACCCAACGCGTTGTTTTCTTATGACGCTTCGACCGCGACCCCAGGTCAGGCGGGACTACCCGCTGAGTTTAAGCATATCAATAAGCGGAGGAA

Supplementary File S2. Alignment of the ITS sequences, based on which the phylogenetic tree (Fig. 6) was constructed. The sequences were aligned using MAFFT and trimmed with SeaView-Gblocks to remove the uninformative characters, before the RAxML analysis (Fig. 6).

>TceratophorumNCBIconsensus

-----------------------------------------------------------------------------------------------------------------------------------------------------------------------------------------------------------------------------------------------------------------------------------------------------------------------------------------tgcagaatcccgtgaaccatcgagtttttgaacgcaagttgcgcccgaagccatccggttcagggcacgcctgcctgggcgtcacgcatcgcgtcgcccccatacttcccttaaaggtagtcgtggtgattgggagcggagattggcctcccgtgcttgttgtgcggttggtctaaataggagtccccttcggtggacacacggctagtggtggttgtaaagacccttttctgctgtgtgttgtgagctgctaggnaaaccttcaaaaaaacccaatgtattgttctaggacgatgcttcgaccggtcaggcgggactacccgctgagttt

>KnoxvilleTN52

a-----------------ccaggcagaacgacctgtgaacacgtaaatacaattgggtgatggggagatggatcttggttctaacacctcctagcgtgcctgcatgctttctcttttgggctatcatgcatgtattgttggaatttaacaaaaccccggcacggcatgtgccaaggaaaacaataaactagaaggactcgacctgatgccccgtttgtggtgtgcattctgagcgtgtcctcctttgaatcacaaacgactctcggcaacggatatctcggctcacgcatcgatgaagaacgtagcaaaatgcgatacttggtgtgaattgcagaatcccgtgaaccatcgagtttttgaacgcaagttgcgcccgaagccatccggttcagggcacgcctgcctgggcgtcacgcatcgcgtcgcccccatacttcccttaagggtagtcgtggcgattgggagcggagattggcttcccgtgcttgttgtgcggttggtcaaaataggagtccccttcggtggacacacggctagtggtggttgtaaagacccttttctgctgtgtgttgtgagctgctagggaaaccctcaaaaaaacccaatgtatcgttctaggacgatgcttcgaccggtcaggcgg-----------------

>KnoxvilleTN53

a-----------------ccaggcagaacgacctgtgaacacgtaaatacaaytgggtgatggggagatggatcttggttctaacacctcctagcgtgcctgcatgctttctcttttgggctatcatgcatgtattgttggaatttaacaaaaccccggcacggcatgtgccaaggaaaacaataaacgagaaggacttgacctgatgccccgtttgtggtgtgcattctgagcgtgtcctcctttgaatcacaaacgactctcggcaacggatatctcggctcacgcatcgatgaagaacgtagcaaaatgcgatacttggtgtgaattgcagaatcccgtgaaccatcgagtttttgaacgcaagttgcgcccgaagccatccggttcagggcacgcctgcctgggcgtcacgcatcgcgtcgcccccatacttcccttaagggtagtcgtggtgattgggagcggagattggcttcccgtgcttgttgtgcggttggtcaaaataggagtccccttyggtggacacacggctagtggtggttgtaaagacccttttctgctgtgtgttgtgagctgctagggaaaccctcaaaaaaacccaatgtatcgttctaggacgatgcttcgaccggtcaggcgg-----------------

>KnoxvilleTN55

aaggatcattgtcgaacccaaggcagaacgacctgtgaacacgtaaatacaactgggtgatggggagatggatcttggttctaacacctcctagcgtgcctgcatgctttctcttttgggctatcatgcatgtattgttggaatttaacaaaaccccggcacggcatgtgccaaggaaaacaataaactagaaggactcgacctgatgccccgtttgtggtgtgcattctgagcgtgtcctcctttgaatcacaaacgactctcggcaacggatatctcggctcacgcatcgatgaagaacgtagcaaaatgcgatacttggtgtgaattgcagaatcccgtgaaccatcgagtttttgaacgcaagttgcgcccgaagccatccggttcagggcacgcctgcctgggcgtcacgcatcgcgtcgcccccatacttcccttaagggtagtcgtggygattgggagcggagattggcttcccgtgcttgttgtgcggttggtcaaaataggagtccccttcggtggacacacggctagtggtggttgtaaagacccttttctgctgtgtgttgtgagctgctagggaaaccctcaaaaaaacccaatgtatcgttctaggacgatgcttcgaccggtcaggcgg-----------------

>KnoxvilleTN52

aaggatcattgtcgaacccaaggcagaacgacctgtgaacacgtaaatacaactgggtgatggggagatggatcttggttctaacacctcctagcgtgcctgcatgctttctcttttgggctatcatgcatgtattgttggaatttaacaaaaccccggcacggcatgtgccaaggaaaacaataaacgagaaggactcgacctgatgccccgtttgtggtgtgcattctgagcgtgtcctcctttgaatcacaaacgactctcggcaacggatatctcggctcacgcatcgatgaagaacgtagcaaaatgcgatacttggtgtgaattgcagaatcccgtgaaccatcgagtttttgaacgcaagttgcgcccgaagccatccggtttagggcacgcctgcctgggcgtcacgcatcgcgtcgcccccatacttcccttaagggtagtcgtggtgattgggagcggagattggcttcccgtgcttgttgtgcggttggtcaaaataggagtccccttcggtggacacacggctagtggtggttgtaaagacccttttctgctgtgtgttgtgagctgctagggaaaccctcaaaaaaacccaatgtatcgttctaggacgatgcttcgaccggtcaggcgg-----------------

>TofficinaleS2

aaggatcattgtcgaacccaaggcagaacgacctgtgaacacgtaaatacaactgggtgatggggagatggatcttggttctaacacctcctagcgtgcctgcatgctttctcttttgggctatcatgcatgtattgttggaatttaacaaaaccccggcacggcatgtgccaaggaaaacaataaacgagaaggactcgacctgatgccccgtttgtggtgtgcattctgagcgtgtcctcctttgaatcacaaacgactctcggcaacggatatctcggctcacgcatcgatgaagaacgtagcaaaatgcgatacttggtgtgaattgcagaatcccgtgaaccatcgagtttttgaacgcaagttgcgcccgaagccatccggtttagggcacgcctgcctgggcgtcacgcatcgcgtcgcccccatacttcccttaagggtagtcgtggtgattgggagcggagattggcttcccgtgcttgttgtgcggttggtcaaaataggagtccccttcggtggacacacggctagtggtggttgtaaagacccttttctgctgtgtgttgtgagctgctagggaaaccctcaaaaaaacccaatgtatcgttctaggacgatgcttcgaccggtcaggcgg-----------------

>KnoxvilleTN54

aaggatcattgtcgaacccaaggcagaacgacctgtgaacacgtaaatacaattgggtgatggggagatggatcttggttctaacacctcctagcgtgcctgcatgctttctcttttgggctatcatgcatgtattgttggaatttaacaaaaccccggcacggcatgtgccaaggaaaacaataaacgagaaggactcgacctgatgccccgtttgtggtgtgcattctgagcgtgtcctcctttgaatcacaaacgactctcggcaacggatatctcggctcacgcatcgatgaagaacgtagcaaaatgcgatacttggtgtgaattgcagaatcccgtgaaccatcgagtttttgaacgcaagttgcgcccgaagccatccggttcagggcacgcctgcctgggcgtcacgcatcgcgtcgcccccatacttcccttaagggtagtcgtggcgattgggagcggagattggcttcccgtgcttgttgtgcggttggtcaaaataggagtccccttcggtggacacacggctagtggtggttgtaaagacccttttctgctgtgtgttgtgagctgctagggaaaccctcaaaaaaacccaatgtatcgttctaggacgatgcttcgaccggtcaggcgggactacccgctgagttt

>PinedaleALM11

aaggatcattgtcgaacccaaggcagaacgacctgtgaacacgtaaatacaactgggtgatggggagatggatcttggttctaacaccttctagcgtgcctgcatgctttctcttttgggctatcatgcatgtattgttggaatttaacaaaaccccggcacggcatgtgccaaggaaaacaataaacgagaaggactcgacctgatgccccgtttgtggtgtgcattctgagcgtgtcctcctttgaatcacaaacgactctcggcaacggatatctcggctcacgcatcgatgaagaacgtagcaaaatgcgatacttggtgtgaattgcagaatcccgtgaaccatcgagtttttgaacgcaagttgcgcccgaagccatccggttcagggcacgcctgcctgggcgtcacgcatcgcgtcgcccccatacttcccttaagggtagtcgtggtgattgggagcggagattggcttcccgtgcttgttgtgcggttggtcaaaataggagtccccttcggtggacacacggctagtggtggttgtaaagacccttttctgctgtgtgttgtgagctgctagggaaaccctcaaaaaaacccaatgtatcgttctaggacgatgcttcgaccggtcaggcgggactacccgctgagttt

>43MileALM19

aaggatcattgtcgaacccaaggcagaacgacctgtgaacacgtaaatacaactgggtgatggggagatggatcttggttctaacacctcctagcgtgcctgcatgctttctcttttgggctatcatgcatgtattgttggaatttaacaaaaccccggcacggcatgtgccaaggaaaacaataaacgagaaggactcgacctgatgccccgtttgtggtgtgcattctgagcgtgtcctcctttgaatcacaaacgactctcggcaacggatatctcggctcacgcatcgatgaagaacgtagcaaaatgcgatacttggtgtgaattgcagaatcccgtgaaccatcgagtttttgaacgcaagttgcgcccgaagccatccggttcagggcacgcctgcctgggcgtcacgcatcgcgtcgcccccatacttcccttaagggtagtcgtggtgattgggagcggagattggcttcccgtgcttgttgtgcggttggtcaaaataggagtccccttcggtggacacacggctagtggtggttgtaaagacccttttctgctgtgtgttgtgagctgctagggaaaccctcaaaaaaacccaatgtatcgttctaggacgatgcttcgaccggtcaggcgggactacccgctgagttt

>MaryvilleTNT4

aaggatcattgtcgaacccaaggcagaacgacctgtgaacacgtaaatacaactgggtgatggggagatggatcttggttctaacacctcctagcgtgcctgcatgctttctcttttgggctatcatgcatgtattgttggaatttaacaaaaccccggcacggcatgtgccaaggaaaacaataaacgagaaggactcgacctgatgccccgtttgtggtgtgcattctgagcgtgtcctcctttgaatcacaaacgactctcggcaacggatatctcggctcacgcatcgatgaagaacgtagcaaaatgcgatacttggtgtgaattgcagaatcccgtgaaccatcgagtttttgaacgcaagttgcgcccgaagccatccggttcagggcacgcctgcctgggcgtcacgcatcgcgtcgcccccatacttcccttaagggtagtcgtggtgattgggagcggagattggcttcccgtgcttgttgtgcggttggtcaaaataggagtccccttcggtggacacacggctagtggtggttgtaaagacccttttctgctgtgtgttgtgagctgctagggaaaccctcaaaaaaacccaatgtatcgttctaggacgatgcttcgaccggtcaggcgggactacccgctgagttt

>TrentonGAM8

aaggatcattgtcgaacccaaggcagaacgacctgtgaacacgtaaatacaactgggtgatggggagatggatcttggttctaacacctcctagcgtgcctgcatgctttctcttttgggctatcatgcatgtattgttggaatttaacaaaaccccggcacggcatgtgccaaggaaaacaataaacgagaaggactcgacctgatgccccgtttgtggtgtgcattctgagcgtgtcctcctttgaatcacaaacgactctcggcaacggatatctcggctcacgcatcgatgaagaacgtagcaaaatgcgatacttggtgtgaattgcagaatcccgtgaaccatcgagtttttgaacgcaagttgcgcccgaagccatccggttcagggcacgcctgcctgggcgtcacgcatcgcgtcgcccccatacttcccttaagggtagtcgtggtgattgggagcggagattggcttcccgtgcttgttgtgcggttggtcaaaataggagtccccttcggtggacacacggctagtggtggttgtaaagacccttttctgctgtgtgttgtgagctgctagggaaaccctcaaaaaaacccaatgtatcgttctaggacgatgcttcgacc---------------------------

>43MileALM23

aaggatcattgtcgaacccaaggcagaacgacctgtgaacacgtaaatacaattgggtgatggggagatggatcttggttctaacacctcctagcgtgcctgcatgctttctcttttgggctatcatgcatgtattgttggaatttaacaaaaccccggcacggcatgtgccaaggaaaacaataaactagaaggactcgacctgatgccccgtttgtggtgtgcattctgagcgtgtcctcctttgaatcacaaacgactctcggcaacggatatctcggctcacgcatcgatgaagaacgtagcaaaatgcgatacttggtgtgaattgcagaatcccgtgaaccatcgagtttttgaacgcaagttgcgcccgaagccatccggttcagggcacgcctgcctgggcgtcacgcatcgcgtcgcccccatacttcccttaagggtagtcgtggcgattgggagcggagattggcttcccgtgcttgttgtgcggttggtcaaaataggagtccccttcggtggacacacggctagtggtggttgtaaagacccttttctgctgtgtgttgtgagctgctagggaaaccctcaaaaaaacccaatgtatcgttctaggacgatgcttcgaccggtcaggcgggactacccgctgagttt

>43MileALM24

aaggatcattgtcgaacccaaggcagaacgacctgtgaacacgtaaatacaattgggtgatggggagatggatcttggttctaacacctcctagcgtgcctgcatgctttctcttttgggctatcatgcatgtattgttggaatttaacaaaaccccggcacggcatgtgccaaggaaaacaataaactagaaggactcgacctgatgccccgtttgtggtgtgcattctgagcgtgtcctcctttgaatcacaaacgactctcggcaacggatatctcggctcacgcatcgatgaagaacgtagcaaaatgcgatacttggtgtgaattgcagaatcccgtgaaccatcgagtttttgaacgcaagttgcgcccgaagccatccggttcagggcacgcctgcctgggcgtcacgcatcgcgtcgcccccatacttcccttaagggtagtcgtggcgattgggagcggagattggcttcccgtgcttgttgtgcggttggtcaaaataggagtccccttcggtggacacacggctagtggtggttgtaaagacccttttctgctgtgtgttgtgagctgctagggaaaccctcaaaaaaacccaatgtatcgttctaggacgatgcttcgaccggtcaggcgggactacccgctgagttt

>43MileALM63

aaggatcattgtcgaacccaaggcagaacgacctgtgaacacgtaaatacaattgggtgatggggagatggatcttggttctaacacctcctagcgtgcctgcatgctttctcttttgggctatcatgcatgtattgttggaatttaacaaaaccccggcacggcatgtgccaaggaaaacaataaactagaaggactcgacctgatgccccgtttgtggtgtgcattctgagcgtgtcctcctttgaatcacaaacgactctcggcaacggatatctcggctcacgcatcgatgaagaacgtagcaaaatgcgatacttggtgtgaattgcagaatcccgtgaaccatcgagtttttgaacgcaagttgcgcccgaagccatccggttcagggcacgcctgcctgggcgtcacgcatcgcgtcgcccccatacttcccttaagggtagtcgtggcgattgggagcggagattggcttcccgtgcttgttgtgcggttggtcaaaataggagtccccttcggtggacacacggctagtggtggttgtaaagacccttttctgctgtgtgttgtgagctgctagggaaaccctcaaaaaaacccaatgtatcgttctaggacgatgcttcgaccggtcaggcgggactacccgctgagttt

>43MileALM64

aaggatcattgtcgaacccaaggcagaacgacctgtgaacacgtaaatacaattgggtgatggggagatggatcttggttctaacacctcctagcgtgcctgcatgctttctcttttgggctatcatgcatgtattgttggaatttaacaaaaccccggcacggcatgtgccaaggaaaacaataaactagaaggactcgacctgatgccccgtttgtggtgtgcattctgagcgtgtcctcctttgaatcacaaacgactctcggcaacggatatctcggctcacgcatcgatgaagaacgtagcaaaatgcgatacttggtgtgaattgcagaatcccgtgaaccatcgagtttttgaacgcaagttgcgcccgaagccatccggttcagggcacgcctgcctgggcgtcacgcatcgcgtcgcccccatacttcccttaagggtagtcgtggcgattgggagcggagattggcttcccgtgcttgttgtgcggttggtcaaaataggagtccccttcggtggacacacggctagtggtggttgtaaagacccttttctgctgtgtgttgtgagctgctagggaaaccctcaaaaaaacccaatgtatcgttctaggacgatgcttcgaccggtcaggcgggactacccgctgagttt

>43mileALM40

aaggatcattgtcgaacccaaggcagaacgacctgtgaacacgtaaatacaattgggtgatggggagatggatcttggttctaacacctcctagcgtgcctgcatgctttctcttttgggctatcatgcatgtattgttggaatttaacaaaaccccggcacggcatgtgccaaggaaaacaataaactagaaggactcgacctgatgccccgtttgtggtgtgcattctgagcgtgtcctcctttgaatcacaaacgactctcggcaacggatatctcggctcacgcatcgatgaagaacgtagcaaaatgcgatacttggtgtgaattgcagaatcccgtgaaccatcgagtttttgaacgcaagttgcgcccgaagccatccggttcagggcacgcctgcctgggcgtcacgcatcgcgtcgcccccatacttcccttaagggtagtcgtggygattgggagcggagattggcttcccgtgcttgttgtgcggttggtcaaaataggagtccccttcggtggacacacggctagtggtggttgtaaagacccttttctgctgtgtgttgtgagctgctagggaaaccctcaaaaaaacccaatgtatcgttctaggacgatgcttcgaccggtcaggcgggactacccgctgagttt

>BoligeeALM32

aaggatcattgtcgaacccaaggcagaacgacctgtgaacacgtaaatacaactgggtgatggggagatggatcttggttctaacgcctcctagcgtgcctgcatgctttctcttttgggctatcatgcatgtattgttggaatttaacaaaaccccggcacggcatgtgccaaggaaaacaataaacgagaaggactcgacctgatgccccgtttgtggtgtgcattctgagcgtgtcctcctttgaatcacaaacgactctcggcaacggatatctcggctcacgcatcgatgaagaacgtagcaaaatgcgatacttggtgtgaattgcagaatcccgtgaaccatcgagtttttgaacgcaagttgcgcccgaagccatccggttcagggcacgcctgcctgggcgtcacgcatcgcgtcgcccccatacttcccttaagggtagtcgtggtgattgggagcggagattggcttcccgtgcttgttgtgcggttggtcaaaataggagtccccttcggtggacacacggctagtggtggttgtaaagacccttttctgctgtgtgttgtgagctgctagggaaaccctcaaaaaaacccaatgtatcgttctaggacgatgcttcgaccggtcaggcgggactacccgctgagttt

>BoligeeALM38

aaggatcattgtcgaacccaaggcagaacgacctgtgaacacgtaaatacaactgggtgatggggagatggatcttggttctaacgcctcctagcgtgcctgcatgctttctcttttgggctatcatgcatgtattgttggaatttaacaaaaccccggcacggcatgtgccaaggaaaacaataaacgagaaggactcgacctgatgccccgtttgtggtgtgcattctgagcgtgtcctcctttgaatcacaaacgactctcggcaacggatatctcggctcacgcatcgatgaagaacgtagcaaaatgcgatacttggtgtgaattgcagaatcccgtgaaccatcgagtttttgaacgcaagttgcgcccgaagccatccggttcagggcacgcctgcctgggcgtcacgcatcgcgtcgcccccatacttcccttaagggtagtcgtggtgattgggagcggagattggcttcccgtgcttgttgtgcggttggtcaaaataggagtccccttcggtggacacacggctagtggtggttgtaaagacccttttctgctgtgtgttgtgagctgctagggaaaccctcaaaaaaacccaatgtatcgttctaggacgatgcttcgaccggtcaggcgggactacccgctgagttt

>PinedaleAL9M36

aaggatcattgtcgaacccaaggcagaacgacctgtgaacacgtaaatacaactgggtgatggggagatggatcttggttctaacaccttctagcgtgcctgcatgctttctcttttgggctatcatgcatgtattgttggaatttaacaaaaccccggcacggcatgtgccaaggaaaacaataaacgagaaggactcgacctgatgccccgtttgtggtgtgcattctgagcgtgtcctcctttgaatcacaaacgactctcggcaacggatatctcggctcacgcatcgatgaagaacgtagcaaaatgcgatacttggtgtgaattgcagaatcccgtgaaccatcgagtttttgaacgcaagttgcgcccgaagccatccggttcagggcacgcctgcctgggcgtcacgcatcgcgtcgcccccatacttcccttaagggtagtcgtggtgattgggagcggagattggcttcccgtgcttgttgtgcggttggtcaaaataggagtccccttcggtggacacacggctagtggtggttgtaaagacccttttctgctgtgtgttgtgagctgctagggaaaccctcaaaaaaacccaatgtatcgttctaggacgatgcttcgaccggtcaggcgggactacccgctgagttt

>PinedaleALM50

aaggatcattgtcgaacccaaggcagaacgacctgtgaacacgtaaatacaactgggtgatggggagatggatcttggttctaacacctyctagcgtgcctgcatgctttctcttttgggctatcatgcatgtattgttggaatttaacaaaaccccggcacggcatgtgccaaggaaaacaataaacgagaaggactcgacctgatgccccgtttgtggtgtgcattctgagcgtgtcctcctttgaatcacaaacgactctcggcaacggatatctcggctcacgcatcgatgaagaacgtagcaaaatgcgatacttggtgtgaattgcagaatcccgtgaaccatcgagtttttgaacgcaagttgcgcccgaagccatccggttcagggcacgcctgcctgggcgtcacgcatcgcgtcgcccccatacttcccttaagggtagtcgtggtgattgggagcggagattggcttcccgtgcttgttgtgcggttggtcaaaataggagtccccttcggtggacacacggctagtggtggttgtaaagacccttttctgctgtgtgttgtgagctgctagggaaaccctcaaaaaaacccaatgtatcgttctaggacgatgcttcgaccggtcaggcgggactacccgctgagttt

>TrentonGAM52

aaggatcattgtcgaacccaaggcagaacgacctgtgaacacgtaaatacaactgggtgatggggagatggatcttggttctaacacctcctagcgtgcctgcatgctttctcttttgggctatcatgcatgtattgttggaatttaacaaaaccccggcacggcatgtgccaaggaaaacaataaacgagaaggactcgacctgatgccccgtttgtggtgtgcattctgagcgtgtcctcctttgaatcacaaacgactctcggcaacggatatctcggctcacgcatcgatgaagaacgtagcaaaatgcgatacttggtgtgaattgcagaatcccgtgaaccatcgagtttttgaacgcaagttgcgcccgaagccatccggttcagggcacgcctgcctgggcgtcacgcatcgcgtcgcccccatacttcccttaagggtagtcgtggtgattgggagcggagattggcttcccgtgcttgttgtgcggttggtcaaaataggagtccccttcggtggacacacggctagtggtggttgtaaagacccttttctgctgtgtgttgtgagctgctagggaaaccctcaaaaaaacccaatgtatcgttctaggacgatgcttcgaccggtcaggcgggactacccgctgagttt

>TrentonGAM55

aaggatcattgtcgaacccaaggcagaacgacctgtgaacacgtaaatacaactgggtgatggggagatggatcttggttctaacacctcctagcgtgcctgcatgctttctcttttgggctatcatgcatgtattgttggaatttaacaaaaccccggcacggcatgtgccaaggaaaacaataaacgagaaggactcgacctgatgccccgtttgtggtgtgcattctgagcgtgtcctcctttgaatcacaaacgactctcggcaacggatatctcggctcacgcatcgatgaagaacgtagcaaaatgcgatacttggtgtgaattgcagaatcccgtgaaccatcgagtttttgaacgcaagttgcgcccgaagccatccggttcagggcacgcctgcctgggcgtcacgcatcgcgtcgcccccatacttcccttaagggtagtcgtggtgattgggagcggagattggcttcccgtgcttgttgtgcggttggtcaaaataggagtccccttcggtggacacacggctagtggtggttgtaaagacccttttctgctgtgtgttgtgagctgctagggaaaccctcaaaaaaacccaatgtatcgttctaggacgatgcttcgaccggtcaggcgggactacccgctgagttt

>KnoxvilleTNT3

aaggatcattgtcgaacccaaggcagaacgacctgtgaacacgtaaatacaactgggtgatggggagatggatcttggttctaacacctcctagcgtgcctgcatgctttctcttttgggctatcatgcatgtattgttggaatttaacaaaaccccggcacggcatgtgccaaggaaaacaataaacgagaaggactcgacctgatgccccgtttgtggtgtgcattctgagcgtgtcctcctttgaatcacaaacgactctcggcaacggatatctcggctcacgcatcgatgaagaacgtagcaaaatgcgatacttggtgtgaattgcagaatcccgtgaaccatcgagtttttgaacgcaagttgcgcccgaagccatccggttcagggcacgcctgcctgggcgtcacgcatcgcgtcgcccccatacttcccttaagggtagtcgtggtgattgggagcggagattggcttcccgtgcttgttgtgcggttggtcaaaataggagtccccttcggtggacacacggctagtggtggttgtaaagacccttttctgctgtgtgttgtgagctgctagggaaaccctcaaaaaaacccaatgtatcgttctaggacgatgcttcgaccggtcaggcgggactacccgctgagttt

>TofficinaleS3

aaggatcattgtcgaacccaaggcagaacgacctgtgaacacgtaaatacaactgggtgatggggagatggatcttggttctaacacctcctagcgtgcctgcatgctttctcttttgggctatcatgcatgtattgttggaatttaacaaaaccccggcacggcatgtgccaaggaaaacaataaacgagaaggactcgacctgatgccccgtttgtggtgtgcattctgagcgtgtcctcctttgaatcacaaacgactctcggcaacggatatctcggctcacgcatcgatgaagaacgtagcaaaatgcgatacttggtgtgaattgcagaatcccgtgaaccatcgagtttttgaacgcaagttgcgcccgaagccatccggtttagggcacgcctgcctgggcgtcacgcatcgcgtcgcccccatacttcccttaagggtagtcgtggtgattgggagcggagattggcttcccgtgcttgttgtgcggttggtcaaaataggagtccccttcggtggacacacggctagtggtggttgtaaagacccttttctgctgtgtgttgtgagctgctagggaaaccctcaaaaaaacccaatgtatcgttctaggacgatgcttcgaccggtcaggcgggactacccgctgagttt

>TrentonGAM56

aaggatcattgtcgaacccaaggcagaacgacctgtgaacacgtaaatacaactgggtgatggggagatggatcttggttctaacacctcctagcgtgcctgcatgctttctcttttgggctatcatgcatgtattgttggaatttaacaaaaccccggcacggcatgtgccaaggaaaacaataaactagaaggactcgacctgatgccccgtttgtggtgtgcattctgagcgtgtcctcctttgaatcacaaacgactctcggcaacggatatctcggctcacgcatcgatgaagaacgtagcaaaatgcgatacttggtgtgaattgcagaatcccgtgaaccatcgagtttttgaacgcaagttgcgcccgaagccatccggttcagggcacgcctgcctgggcgtcacgcatcgcgtcgcccccatacttcccttaagggtagtcgtggtgattgggagcggagattggcttcccgtgcttgttgtgcggttggtcaaaataggagtccccttcggtggacacacggctagtggtggttgtaaagacccttttctgctgtgtgttgtgagctgctagggaaaccctcaaaaaaacccaatgtatcgttctaggacgatgcttcgaccggtcaggcgggactacccgctgagttt

>43mileALM62

aaggatcattgtcgaacccaaggcagaacgacctgtgaacacgtaaatacaactgggtgatggggagatggatcttggttctaacacctcctagcgtgcctgcatgctttctcttttgggctatcatgcatgtattgttggaatttaacaaaaccccggcacggcatgtgccaaggaaaacaataaackagaaggactcgacctgatgccccgtttgtggtgtgcattctgagcgtgtcctcctttgaatcacaaacgactctcggcaacggatatctcggctcacgcatcgatgaagaacgtagcaaaatgcgatacttggtgtgaattgcagaatcccgtgaaccatcgagtttttgaacgcaagttgcgcccgaagccatccggttcagggcacgcctgcctgggcgtcacgcatcgcgtcgcccccatacttcccttaagggtagtcgtggtgattgggagcggagattggcttcccgtgcttgttgtgcggttggtcaaaataggagtccccttcggtggacacacggctagtggtggttgtaaagacccttttctgctgtgtgttgtgagctgctagggaaaccctcaaaaaaacccaatgtatcgttctaggacgatgcttcgaccggtcaggcgggactacccgctgagttt

>KnoxvilleTNT1

aaggatcattgtcgaacccaaggcagaacgacctgtgaacacgtaaatacaactgggtgatggggagatggatcttggttctaacacctcctagcgtgcctgcatgctttctcttttgggctatcatgcatgtattgttggaatttaacaaaaccccggcacggcatgtgccaaggaaaacaataaacgagaaggactcgacctgatgccccgtttgtggtgtgcattctgagcgtgtcctcctttgaatcacaaacgactctcggcaacggatatctcggctcacgcatcgatgaagaacgtagcaaaatgcgatacttggtgtgaattgcagaatcccgtgaaccatcgagtttttgaacgcaagttgcgcccgaagccatccggtttagggcacgcctgcctgggcgtcacgcatcgcgtcgcccccatacttcccttaagggtagtcgtggtgattgggagcggagattggcttcccgtgcttgttgtgcggttggtcaaaataggagtccccttcggtggacacacggctagtggtggttgtaaagacccttttctgctgtgtgttgtgagctgctagggaaaccctcaaataaacccaatgtatcgttctaggacgatgcttcgaccggtcaggcgggactacccgctgagttt

>MaryvilleTNT12

aaggatcattgtcgaacccaaggcagaacgacctgtgaacacgtaaatacaactgggtgatggggagatggatcttggttctaacacctcctagcgtgcctgcatgctttctcttttgggctatcatgcatgtattgttggaatttaacaaaaccccggcacggcatgtgccaaggaaaacaataaacgagaaggactcgacctgatgccccgtttgtggtgtgcattctgagcgtgtcctcctttgaatcacaaacgactctcggcaacggatatctcggctcacgcatcgatgaagaacgtagcaaaatgcgatacttggtgtgaattgcagaatcccgtgaaccatcgagtttttgaacgcaagttgcgcccgaagccatccggtttagggcacgcctgcctgggcgtcacgcatcgcgtcgcccccatacttcccttaagggtagtcgtggtgattgggagcggagattggcttcccgtgcttgttgtgcggttggtcaaaataggagtccccttcggtggacacacggctagtggtggttgtaaagacccttttctgctgtgtgttgtgagctgctagggaaaccctcaaataaacccaatgtatcgttctaggacgatgcttcgaccggtcaggcgggactacccgctgagttt

>WelcomeRaMSM31

aaggatcattgtcgaacccaaggcagaacgacctgtgaacacgtaaatacaactgggtgatggggagatggatcttggttctaacaccttctagcgtgcctgcatgctttctcttttgggctatcatgcatgtattgttggaatttaacaaaaccccggcacggcatgtgccaaggaaaacaataaacgagaaggactcgacctgatgccccgtttgtggtgtgcattctgagcgtgtcctcctttgaatcacaaacgactctcggcaacggatatctcggctcacgcatcgatgaagaacgtagcaaaatgcgatacttggtgtgaattgcagaatcccgtgaaccatcgagtttttgaacgcaagttgcgcccgaagccatccggtttagggcacgcctgcctgggcgtcacgcatcgcgtcgcccccatacttcccttaagggtagtcgtggtgattgggagcggagattggcttcccgtgcttgttgtgcggttggtcaaaataggagtccccttcggtggacacacggctagtggtggttgtaaagacccttttctgctgtgtgttgtgagctgctagggaaaccctcatataaacccaatgtatcgttctaggacgatgcttcgaccggtcaggcgggactacccgctgagttt

>WelcomeRaMSM33

aaggatcattgtcgaacccaaggcagaacgacctgtgaacacgtaaatacaactgggtgatggggagatggatcttggttctaacaccttctagcgtgcctgcatgctttctcttttgggctatcatgcatgtattgttggaatttaacaaaaccccggcacggcatgtgccaaggaaaacaataaacgagaaggactcgacctgatgccccgtttgtggtgtgcattctgagcgtgtcctcctttgaatcacaaacgactctcggcaacggatatctcggctcacgcatcgatgaagaacgtagcaaaatgcgatacttggtgtgaattgcagaatcccgtgaaccatcgagtttttgaacgcaagttgcgcccgaagccatccggtttagggcacgcctgcctgggcgtcacgcatcgcgtcgcccccatacttcccttaagggtagtcgtggtgattgggagcggagattggcttcccgtgcttgttgtgcggttggtcaaaataggagtccccttcggtggacacacggctagtggtggttgtaaagacccttttctgctgtgtgttgtgagctgctagggaaaccctcatataaacccaatgtatcgttctaggacgatgcttcgaccggtcaggcgggactacccgctgagttt

>WelcomeRaMSM44

aaggatcattgtcgaacccaaggcagaacgacctgtgaacacgtaaatacaactgggtgatggggagatggatcttggttctaacaccttctagcgtgcctgcatgctttctcttttgggctatcatgcatgtattgttggaatttaacaaaaccccggcacggcatgtgccaaggaaaacaataaacgagaaggactcgacctgatgccccgtttgtggtgtgcattctgagcgtgtcctcctttgaatcacaaacgactctcggcaacggatatctcggctcacgcatcgatgaagaacgtagcaaaatgcgatacttggtgtgaattgcagaatcccgtgaaccatcgagtttttgaacgcaagttgcgcccgaagccatccggtttagggcacgcctgcctgggcgtcacgcatcgcgtcgcccccatacttcccttaagggtagtcgtggtgattgggagcggagattggcttcccgtgcttgttgtgcggttggtcaaaataggagtccccttcggtggacacacggctagtggtggttgtaaagacccttttctgctgtgtgttgtgagctgctagggaaaccctcatataaacccaatgtatcgttctaggacgatgcttcgaccggtcaggcgggactacccgctgagttt

>WelcomeRaMSM48

aaggatcattgtcgaacccaaggcagaacgacctgtgaacacgtaaatacaactgggtgatggggagatggatcttggttctaacaccttctagcgtgcctgcatgctttctcttttgggctatcatgcatgtattgttggaatttaacaaaaccccggcacggcatgtgccaaggaaaacaataaacgagaaggactcgacctgatgccccgtttgtggtgtgcattctgagcgtgtcctcctttgaatcacaaacgactctcggcaacggatatctcggctcacgcatcgatgaagaacgtagcaaaatgcgatacttggtgtgaattgcagaatcccgtgaaccatcgagtttttgaacgcaagttgcgcccgaagccatccggtttagggcacgcctgcctgggcgtcacgcatcgcgtcgcccccatacttcccttaagggtagtcgtggtgattgggagcggagattggcttcccgtgcttgttgtgcggttggtcaaaataggagtccccttcggtggacacacggctagtggtggttgtaaagacccttttctgctgtgtgttgtgagctgctagggaaaccctcatataaacccaatgtatcgttctaggacgatgcttcgaccggtcaggcgggactacccgctgagttt

>WelcomeraMSM53

aaggatcattgtcgaacccaaggcagaacgacctgtgaacacgtaaatacaactgggtgatggggagatggatcttggttctaacaccttctagcgtgcctgcatgctttctcttttgggctatcatgcatgtattgttggaatttaacaaaaccccggcacggcatgtgccaaggaaaacaataaacgagaaggactcgacctgatgccccgtttgtggtgtgcattctgagcgtgtcctcctttgaatcacaaacgactctcggcaacggatatctcggctcacgcatcgatgaagaacgtagcaaaatgcgatacttggtgtgaattgcagaatcccgtgaaccatcgagtttttgaacgcaagttgcgcccgaagccatccggtttagggcacgcctgcctgggcgtcacgcatcgcgtcgcccccatacttcccttaagggtagtcgtggtgattgggagcggagattggcttcccgtgcttgttgtgcggttggtcaaaataggagtccccttcggtggacacacggctagtggtggttgtaaagacccttttctgctgtgtgttgtgagctgctagggaaaccctcatataaacccaatgtatcgttctaggacgatgcttcgaccggtcaggcgggactacccgctgagttt

>TrentonGAM15

aaggatcattgtcgaacccaaggcagaacgacctgtgaacacgtaaatacaactgggtgatggggagatggatcttggttctaacacctcctagcgtgcctgcatgctttctcttttgggctatcatgcatgtattgttggaatttaacaaaaccccggcacggcatgtgccaaggaaaacaataaactagaaggactcgacctgatgccccgtttgtggtgtgcattctgagcgtgtcctcctttgaatcacaaacgactctcggcaacggatatctcggctcacgcatcgatgaagaacgtagcaaaatgcgatacttggtgtgaattgcagaatcccgtgaaccatcgagtttttgaacgcaagttgcgcccgaagccatccggttcagggcacgcctgcctgggcgtcacgcatcgcgtcgcccccatacttcccttaagggtagtcgtggtgattgggagcggagattggcttcccgtgcttgttgtgcggttggtcaaaataggagtccccttcggtggacacacggctagtggtggttgtaaagacccttttctgctgtgtgttgtgagctgctagggaaaccctcaaaaaaacccaatgtatcgttctaggacgatgcttcgaccggtcaggcgggactacccgctgagttt

>TrentonGAM16

aaggatcattgtcgaacccaaggcagaacgacctgtgaacacgtaaatacaactgggtgatggggagatggatcttggttctaacacctcctagcgtgcctgcatgctttctcttttgggctatcatgcatgtattgttggaatttaacaaaaccccggcacggcatgtgccaaggaaaacaataaactagaaggactcgacctgatgccccgtttgtggtgtgcattctgagcgtgtcctcctttgaatcacaaacgactctcggcaacggatatctcggctcacgcatcgatgaagaacgtagcaaaatgcgatacttggtgtgaattgcagaatcccgtgaaccatcgagtttttgaacgcaagttgcgcccgaagccatccggttcagggcacgcctgcctgggcgtcacgcatcgcgtcgcccccatacttcccttaagggtagtcgtggtgattgggagcggagattggcttcccgtgcttgttgtgcggttggtcaaaataggagtccccttcggtggacacacggctagtggtggttgtaaagacccttttctgctgtgtgttgtgagctgctagggaaaccctcaaaaaaacccaatgtatcgttctaggacgatgcttcgaccggtcaggcgggactacccgctgagttt

>BoligeeALM13

aaggatcattgtcgaacccaaggcagaacgacctgtgaacacgtaaatacaactgggtgatggggagatggatcttggttctaacgcctcctagcgtgcctgcatgctttctcttttgggctatcatgcatgtattgttggaatttaacaaaaccccggcacggcatgtgccaaggaaaacaataaacgagaaggactcgacctgatgccccgtttgtggtgtgcattctgagcgtgtcctcctttgaatcacaaacgactctcggcaacggatatctcggctcacgcatcgatgaagaacgtagcaaaatgcgatacttggtgtgaattgcagaatcccgtgaaccatcgagtttttgaacgcaagttgcgcccgaagccatccggttcagggcacgcctgcctgggcgtcacgcatcgcgtcgcccccatacttcccttaagggtagtcgtggtgattgggagcggagattggcttcccgtgcttgttgtgcggttggtcaaaataggagtccccttcggtggacacacggctagtggtggttgtaaagacccttttctgctgtgtgttgtgagctgctagggaaaccctcaaaaaaacccaatgtatcgttctaggacgatgcttcgacc---------------------------

>WelcomeRaMSM2

aaggatcattgtcgaacccaaggcagaacgacctgtgaacacgtaaatacaactgggtgatggggagatggatcttggttctaacaccttctagcgtgcctgcatgctttctcttttgggctatcatgcatgtattgttggaatttaacaaaaccccggcacggcatgtgccaaggaaaacaataaacgagaaggactcgacctgatgccccgtttgtggtgtgcattctgagcgtgtcctcctttgaatcacaaacgactctcggcaacggatatctcggctcacgcatcgatgaagaacgtagcaaaatgcgatacttggtgtgaattgcagaatcccgtgaaccatcgagtttttgaacgcaagttgcgcccgaagccatccggtttagggcacgcctgcctgggcgtcacgcatcgcgtcgcccccatacttcccttaagggtagtcgtggtgattgggagcggagattggcttcccgtgcttgttgtgcggttggtcaaaataggagtccccttcggtggacacacggctagtggtggttgtaaagacccttttctgctgtgtgttgtgagctgctagggaaaccctcaaataaacccaatgtatcgttctaggacgatgcttcgacc---------------------------

>TrentonGAM7

aaggatcattgtcgaacccaaggcagaacgacctgtgaacacgtaaatacaactgggtgatggggagatggatcttggttctaacaccttctagcgtgcctgcatgctttctcttttgggctatcatgcatgtattgttggaatttaacaaaaccccggcacggcatgtgccaaggaaaacaataaacgagaaggactcgacctgatgccccgtttgtggtgtgcattctgagcgtgtcctcctttgaatcacaaacgactctcggcaacggatatctcggctcacgcatcgatgaagaacgtagcaaaatgcgatacttggtgtgaattgcagaatcccgtgaaccatcgagtttttgaacgcaagttgcgcccgaagccatccggttcagggcacgcctgcctgggcgtcacgcatcgcgtcgcccccatacttcccttaagggtagtcgtggtgattgggagcggagattggcttcccgtgcttgttgtgcggttggtcaaaataggagtccccttcggtggacacacggctagtggtggttgtaaagacccttttctgctgtgtgttgtgagctgctagggaaaccctcaaataaacccaatgtatcgttctaggacgatgcttcgacc---------------------------

>KnoxvilleTNT3

aaggatcattgtcgaacccaaggcagaacgacctgtgaacacgtaaatacaactgggtgatggggagatggatcttggttctaacaccttctagcgtgcctgcatgctttctcttttgggctatcatgcatgtattgttggaatttaacaaaaccccggcacggcatgtgccaaggaaaacaataaacgagaaggactygacctgatgccccgtttgtggtgtgcattctgagcgtgtcctcctttgaatcacaaacgactctcggcaacggatatctcggctcacgcatcgatgaagaacgtagcaaaatgcgatacttggtgtgaattgcagaatcccgtgaaccatcgagtttttgaacgcaagttgcgcccgaagccatccggttcagggcacgcctgcctgggcgtcacgcatcgcgtcgcccccatacttcccttaagggtagtcgtggtgattgggagcggagattggcttcccgtgcttgttgtgcggttggtcaaaataggagtccccttcggtggacacacggctagtggtggttgtaaagacccttttctgctgtgtgttgtgagctgctagggaaaccctcaaaaaaacccaatgtatcgttctaggacgatgcttcgaccggtcaggcgggactacccgctgagttt

>BoligeeALM29

aaggatcattgtcgaacccaaggcagaacgacctgtg-acacgtaaatacaactgggtgatggggagatggatcttggttctaacgcctcctagcgtgcctgcatgctttctcttttgggctatcatgcatgtattgttggaatttaacaaaaccccggcacggcatgtgccaaggaaaacaataaacgagaaggactcgacctgatgccccgtttgtggtgtgcattctgagcgtgtcctcctttgaatcacaaacgactctcggcaacggatatctcggctcacgcatcgatgaagaacgtagcaaaatgcgatacttggtgtgaattgcagaatcccgtgaaccatcgagtttttgaacgcaagttgcgcccgaagccatccggttcagggcacgcctgcctgggcgtcacgcatcgcgtcgcccccatacttcccttaagggtagtcgtggtgattgggagcggagattggcttcccgtgcttgttgtgcggttggtcaaaataggagtccccttcggtggacacacggctagtggtggttgtaaagacccttttctgctgtgtgttgtgagctgctagggaaaccctcaaaaaaacccaatgtatcgttctaggacgatgcttcgaccggtcaggcgggactacccgctgagttt

>43MileALM39

aaggatcattgtcgaacccaaggcagaacgacctgtg-acacgtaaatacaactgggtgatggggagatggatcttggttctaacrcctcctagcgtgcctgcatgctttctcttttgggctatcatgcatgtattgttggaatttaacaaaaccccggcacggcatgtgccaaggaaaacaataaacgagaaggactcgacctgatgccccgtttgtggtgtgcattctgagcgtgtcctcctttgaatcacaaacgactctcggcaacggatatctcggctcacgcatcgatgaagaacgtagcaaaatgcgatacttggtgtgaattgcagaatcccgtgaaccatcgagtttttgaacgcaagttgcgcccgaagccatccggttcagggcacgcctgcctgggcgtcacgcatcgcgtcgcccccatacttcccttaagggtagtcgtggtgattgggagcggagattggcttcccgtgcttgttgtgcggttggtcaaaataggagtccccttcggtggacacacggctagtggtggttgtaaagacccttttctgctgtgtgttgtgagctgctagggaaaccctcaaaaaaacccaatgtatcgttctaggacgatgcttcgaccggtcaggcgggactacccgctgagttt

>Tuscaloosa59SraALM47

aaggatcattgtcgaacccaaggcagaacgacctgtg-acacgtaaatacaactgggtgatggggagatggatcttggttctaacacctcctagcgtgcctgcatgctttctcttttgggctatcatgcatgtattgttggaatttaacaaaaccccggcacggcatgtgccaaggaaaacaataaacgagaaggactcgacctgatgccccgtttgtggtgtgcattctgagcgtgtcctcctttgaatcacaaacgactctcggcaacggatatctcggctcacgcatcgatgaagaacgtagcaaaatgcgatacttggtgtgaattgcagaatcccgtgaaccatcgagtttttgaacgcaagttgcgcccgaagccatccggttcagggcacgcctgcctgggcgtcacgcatcgcgtcgcccccatacttcccttaagggtagtcgtggtgattgggagcggagattggcttcccgtgcttgttgtgcggttggtcaaaataggagtccccttcggtggacacacggctagtggtggttgtaaagacccttttctgctgtgtgttgtgagctgctagggaaaccctcaaaaaaacccaatgtatcgttctaggacgatgcttcgaccggtcaggcgggactacccgctgagttt

>KnoxvilleTN53

aaggatcattgtcgaacccaaggcagaacgacctgtgaacacgtaaatacaactgggtgatggggagatggatcttggttctaacacctcctagcgtgcctgcatgctttctcttttgggctatcatgcatgtattgttggaatttaacaaaaccccggcacggcatgtgccaaggaaaacaataaacgagaaggactcgacctgatgccccgtttgtggtgtgcattctgagcgtgtcctcctttgaatcacaaacgactctcggcaacggatatctcggctcacgcatcgatgaagaacgtagcaaaatgcgatacttggtgtgaattgcagaatcccgtgaaccatcgagtttttgaacgcaagttgcgcccgaagccatccggtttagggcacgcctgcctgggcgtcacgcatcgcgtcgcccccatacttcccttaagggtagtcgtggtgattgggagcggagattgacttcctgtgcttgttgtgcggttggtcaaaataggagtccccttcggtggacacacggctagtggtggttgtaaagacccttttctgctgtgtgttgtgagctgctagggaaaccctcaaaaaaacccaatgtatcgttctaggacgatgcttcgaccggtcaggcgggactacccgctgagttt

>WelcomeRaMSM5

aaggatcattgtcgaacccaaggcagaacgacctgtgaacacgtaaatacaactgggtgatggggagatggatcttggttctaacaccttctagcgtgcctgcatgctttctcttttgggctatcatgcatgtattgttggaatttaacaaaaccccggcacggcatgtgccaaggaaaacaataaacgagaaggactcgacctgatgccccgtttgtggtgtgcattctgagcgtgtcctcctttgaatcacaaacgactctcggcaacggatatctcggctcacgcatcgatgaagaacgtagcaaaatgcgatacttggtgtgaattgcagaatcccgtgaaccatcgagtttttgaacgcaagttgcgcccgaagccatccggtttagggcacgcctgcctgggcgtcacgcatcgcgtcgcccccatacttcccttaagggtagtcgtggtgattgggagcggagattggcttcccgtgcttgttgtgcggttggtcaaaataggagtccccttcggtggacacacggctagtggtggttgtaaagacccttttctgctgtgtgttgtgagctgctagggaaaccctcaaaaaaacccaatgtatcgttctaggacgatgcttcgaccggtcaggcgggactacccgctgagttt

>BoligeeALM22

aaggatcattgtcgaacccaaggcagaacgacctgtgaacacgtaaatacaactgggtgatggggagatggatcttggttctaacgcctcctagcgtgcctgcatgctttctcttttgggctatcatgcatgtattgttggaatttaacaaaaccccggcacggcatgtgccaaggaaaacaataaacgagaaggactcgacctgatgccccgtttgtggtgtgcattctgagcgtgtcctcctttgaatcacaaacgactctcggcaacggatatctcggctcacgcatcgatgaagaacgtagcaaaatgcgatacttggtgtgaattgcagaatcccgtgaaccatcgagtttttgaacgcaagttgcgcccgaagccatccggttcagggcacgcctgcctgggcgtcacgcatcgcgtcgcccccatacttcccttaagggtagtcgtggtgattgggagcggagattggcttcccgtgcttgttgtgcggttggtcaaaataggagtccccttcggtggacacacggctagtggtggttgtaaagacccttttctgctgtgtgttgtgagctgctagggaaaccctcaaaaaaacccaatgtatcgttctaggacgatgcttcgaccggtcaggcgggactacccgctgagttt

>43MileALM25

aaggatcattgtcgaacccaaggcag-acgacctgtg-acacgtaaatacaattgggtgatggggagatggatcttggttctaacacctcctagcgtgcctgcatgctttctcttttgggctatcatgcatgtattgttggaatttaacaaaaccccggcacggcatgtgccaaggaaaacaataaactagaaggactcgacctgatgccccgtttgtggtgtgcattctgagcgtgtcctcctttgaatcacaaacgactctcggcaacggatatctcggctcacgcatcgatgaagaacgtagcaaaatgcgatacttggtgtgaattgcagaatcccgtgaaccatcgagtttttgaacgcaagttgcgcccgaagccatccggttcagggcacgcctgcctgggcgtcacgcatcgcgtcgcccccatacttcccttaagggtagtcgtggcgattgggagcggagattggcttcccgtgcttgttgtgcggttggtcaaaataggagtccccttcggtggacacacggctagtggtggttgtaaagacccttttctgctgtgtgttgtgagctgctagggaaaccctcaaaaaaacccaatgtatcgttctaggacgatgcttcgaccggtcaggcgggactacccgctgagttt

>PinedaleALM1

aaggatcattgtcgaacccaaggcagaacgacctgtgaacacgtaaatacaattgggtgatggggagatggatcttggttctaacacctcctagcgtgcctgcatgctttctcttttgggctatcatgcatgtattgttggaatttaacaaaaccccggcacggcatgtgccaaggaaaacaataaactagaaggactcgacctgatgccccgtttgtggtgtgcattctgagcgtgtcctcctttgaatcacaaacgactctcggcaacggatatctcggctcacgcatcgatgaagaacgtagcaaaatgcgatacttggtgtgaattgcagaatcccgtgaaccatcgagtttttgaacgcaagttgcgcccgaagccatccggttcagggcacgcctgcctgggcgtcacgcatcgcgtcgcccccatacttcccttaagggtagtcgtggcgattgggagcggagattggcttcccgtgcttgttgtgcggttggtcaaaataggagtccccttcggtggacacacggctagtggtggttgtaaagacccttttctgctgtgtgttgtgagctgctagggaaaccctcaaaaaaacccaatgtatcgttctaggacgatgcttcgaccggtcaggcgggactacccgctgagttt

>KnoxvilleTNT4

aaggatcattgtcgaacccaaggcagaacgacctgtgaacacgtaaatacaattgggtgatggggagatggatcttggttctaacacctcctagcgtgcctgcatgctttctcttttgggctatcatgcatgtattgttggaatttaacaaaaccccggcacggcatgtgccaaggaaaacaataaactagaaggactcgacctgatgccccgtttgtggtgtgcattctgagcgtgtcctcctttgaatcacaaacgactctcggcaacggatatctcggctcacgcatcgatgaagaacgtagcaaaatgcgatacttggtgtgaattgcagaatcccgtgaaccatcgagtttttgaacgcaagttgcgcccgaagccatccggttcagggcacgcctgcctgggcgtcacgcatcgcgtcgcccccatacttcccttaagggtagtcgtggcgattgggagcggagattggcttcccgtgcttgttgtgcggttggtcaaaataggagtccccttcggtggacacacggctagtggtggttgtaaagacccttttctgctgtgtgttgtgagctgctagggaaaccctcaaaaaaacccaatgtatcgttctaggacgatgcttcgaccggtcaggcgggactacccgctgagttt

>WelcomeRaMSM27

aaggatcattgtcgaacccaaggcagaacgacctttgaacacgtaaatacaactgggtgatggggagatggatcttggttctaacaccttctagcgtgcctgcatgctttctcttttgggctatcatgcatgtattgttggaatttaacaaaaccccggcacggcatgtgccaaggaaaacaataaacgagaaggactcgacctgatgccccgtttgtggtgtgcattctgagcgtgtcctcctttgaatcacaaacgactctcggcaacggatatctcggctcacgcatcgatgaagaacgtagcaaaatgcgatacttggtgtgaattgcagaatcccgtgaaccatcgagtttttgaacgcaagttgcgcccgaagccatccggtttagggcacgcctgcctgggcgtcacgcatcgcgtcgcccccatacttcccttaagggtagtcgtggtgattgggagcggagattggcttcccgtgcttgttgtgcggttggtcaaaataggagtccccttcggtggacacacggctagtggtggttgtaaagacccttttctgctgtgtgttgtgagctgctagggaaaccctcatataaacccaatgtatcgttctaggacgatgcttcgaccggtcaggcgggactacccgctgagttt

>WelcomeRaMSM4

aaggatcattgtcgaacccaaggccgaacgacctgtgaacacgtaaatacaactgggtgatggggagatggatcttggttctaacaccttctagcgtgcctgcatgctttctcttttgggctatcatgcatgtattgttggaatttaacaaaaccccggcacggcatgtgccaaggaaaacaataaacgagaaggactcgacctgatgccccgtttgtggtgtgcattctgagcgtgtcctcctttgaatcacaaacgactctcggcaacggatatctcggctcacgcatcgatgaagaacgtagcaaaatgcgatacttggtgtgaattgcagaatcccgtgaaccatcgagtttttgaacgcaagttgcgcccgaagccatccggtttagggcacgcctgcctgggcgtcacgcatcgcgtcgcccccatacttcccttaagggtagtcgtggtgattgggagcggagattggcttcccgtgcttgttgtgcggttggtcaaaataggagtccccttcggtggacacacggctagtggtggttgtaaagacccttttctgctgtgtgttgtgagctgctagggaaaccctcaaaaaaacccaatgtatcgttctaggacgatgcttcgaccggtcaggcgggactacccgctgagttt

>TofficinaleS1

aaggatcattgtcgaacccaaggcagaacgacctgtgaacacgtaaatacaactgggtgatggggagatggatcttggttctaacacctcctagcgtgcctgcatgctttctcttttgggctatcatgcatgtattgttggaatttaacaaaaccccggcacggcatgtgccaaggaaaacaataaacgagaaggactcgacctgatgccccgtttgtggtgtgcattctgagcgtgtcctcctttgaatcacaaacgactctcggcaacggatatctcggctcacgcatcgatgaagaacgtagcaaaatgcgatacttggtgtgaattgcagaatcccgtgaaccatcgagtttttgaacgcaagttgcgcccgaagccatccggtttagggcacgcctgcctgggcgtcacgcatcgcgtcgcccccatacttcccttaagggtagtcgtggtgattgggagcggagattggcttcccgtgcttg-tgtgcggttggtcaaaataggagtccccttcggtggacacacggctagtggtggttgtaaagacccttttctgctgtgtg-tgtgagctgctagggaaaccctcaaaaaaacccaatgtatcgttctaggacgatgcttcgaccggtcaggcgggactacccgctgagttt

>BoligeeALM20

aaggatcattgtcgaacccaaggcag-acgacctcgtgacacgtaaatacaactgggtgatggggagatggatcttgattctaacacctcctagcgtgcctgcatgctttctcttttgggctatcatgcatgtattgttggaatttaacaaaaccccggcacggcatgtgccaaggaaaacaataaacgagaaggactcgacctgatgccccgtttgtggtgtgcattctgagcgtgtcctcctttgaatcacaaacgactctcggcaacggatatctcggctcacgcatcgatgaagaacgtagcaaaatgcgatacttggtgtgaattgcagaatcccgtgaaccatcgagtttttgaacgcaagttgcgcccgaagccatccggttcagggcacgcctgcctgggcgtcacgcatcgcgtcgcccccatacttcccttaagggtagtcgtggtgattgggagcggagattggcttcccgtgcttgttgtgcggttggtcaaaataggagtccccttcggtggacacatggctagtggtggttgtaaagacccttttctgctgtgtgttgtgagctgctagggaaaccctcaaaaaaacccaatgtatcgttctaggacgatgcttcgaccggtcaggcgggactacccgctgagttt

>Tuscaloosa59SraALM65

aaggatcattgtcgaacccatggcagaacgacctgtgaacacgtaaatacaactgggtgatggggagatggatcttgattctaacacctcctagcgtgcctgcatgctttctcttttgggctatcatgcatgtattgttggaatttaacaaaaccccggcacggcatgtgccaaggaaaacaataaatgagaaggactcgacctgatgccccgtttgtggtgtgcattctgagcgtgtcctcctttgaatcacaaacgactctcggcaacggatatctcggctcacgcatcgatgaagaacgtagcaaaatgcgatacttggtgtgaattgcagaatcccgtgaaccatcgagtttttgaacgcaagttgcgcccgaagccatccggttcagggcacgcctgcctgggcgtcacgcatcgcgtcgcccccatacttcccttaagggtagtcgtggtgattgggagcggagattggcttcccgtgcttgtggtgcggttggtcaaaataggagtccccttcggtggacacacggctagtggtggttgtaaagacccttttctgctgtgtgttgtgagctgctagggaaaccctcaaaaaaacccaatgtatcgttctaggacgatgcttcgaccggtcaggcgggactacccgctgagttt

>MaryvilleTNK6

aaggatcatngtcgaacccatggcagaacgacctgtgtacatgtaaatacaat-gggtgatggggaaatggatcttggttctaacacctcctagcgtgcctgcatgctttctcttttgggctatcatgcatgtattgttggaatttaacaaaaccccggcacggcatgtgccaaggaaaacaaaaaacgagaaggactcgacctgatgccccgtttgtggtgtgcattctgagcgtgtcctcctttgaatcacaaacgactctcggcaacggatatctcggctcacgcatcgatgaagaacgtagcaaaatgcgatacttggtgtgaattgcagaatcccgtgaaccatcgagtttttgaacgcaagttgcgcctgaagccatccggttgagggcacgcctgcctgggcgtcacgcatcgcgttgcccccatacttcccttaagggtagtcgtggtgattgggagcggagattggcctcccgtacttgtggtgcggttggtcaaaataggagtccccttcggtggacacacggctagtggtggttgtaaagacccttttctgctgtgtgttgtgagctgtttgggaaaccctcaaaaaaccccaatgtgtcgttctaggatgatat----------------------------------

>PinedaleALM12

aaggatcattgtcgaacccatggcagaacgacctgtgaacatgtaaatacaat-gggtgatggggaaatggatcttggttctaacacctcctagcgtgcctgcatgctttctcttttgggctatcatgcatgtattgttggaatttaacaaaaccccggcacggcatgtgccaaggaaaacaaaaaacgagaaggactcgacctgatgccccgtttgtggtgtgcattctgagcgtgtcctcctttgaatcacaaacgactctcggcaacggatatctcggctcacgcatcgatgaagaacgtagcaaaatgcgatacttggtgtgaattgcagaatcccgtgaaccatcgagtttttgaacgcaagttgcgcctgaagccatccggttgagggcacgcctgcctgggcgtcacgcatcgcgttgcccccatacttcccttaagggtagtcgtggtgattgggagcggagattggcctcccgtacttgtggtgcggttggtcaaaataggagtccccttcggtggacacacggctagtggtggttgtaaagacccttttctgctgtgtgttgtgagctgtttgggaaaccctcaaataaccccaatgtgtcgttctaggatgatacttcgaccggtcaggcgggactacccgctgagttt

>PinedaleALM26

aaggatcattgtcgaacccatggcagaacgacctgtgaacatgtaaatacaattgggtgatggggaaatggatcttggttctaacacctcctagcgtgcctgcatgctttctcttttgggctatcatgcatgtattgttggaatttaacaaaaccccggcacggcatgtgccaaggaaaacaaaaaacgagaaggactcgacctgatgccccgtttgtggtgtgcattctgagcgtgtcctcctttgaatcacaaacgactctcggcaacggatatctcggctcacgcatcgatgaagaacgtagcaaaatgcgatacttggtgtgaattgcagaatcccgtgaaccatcgagtttttgaacgcaagttgcgcctgaagccatccggttgagggcacgcctgcctgggcgtcacgcatcgcgttgcccccatacttcccttaagggtagtcgtggtgattgggagcggagattggcctcccgtacttgtggtgcggttggtcaaaataggagtccccttcggtggacacacggctagtggtggttgtaaagacccttttctgctgtgtgttgtgagctgtttgggaaaccctcaaaaaaccccaatgtgtcgttctaggatgatacttcgaccggtcaggcgggactacccgctgagttt

>MaryvilleTNT6

aaggatcattgtcgaacccatggcagaacgacctgtgaacatgtaaatacaatcgggtgatggggaaatggatcttggttctaacacctcctagcgtgcctgcatgctttctcttttgggctatcatgcatgtattgttggaatttaacaaaaccccggcacggcatgtgccaaggaaaacaaaaaacgagaaggactcgacctgatgccccgtttgtggtgtgcattctgagcgtgtcctcctttgaatcacaaacgactctcggcaacggatatctcggctcacgcatcgatgaagaacgtagcaaaatgcgatacttggtgtgaattgcagaatcccgtgaaccatcgagtttttgaacgcaagttgcgcctgaagccatccggttgagggcacgcctgcctgggcgtcacgcatcgcgttgcccccatacttcccttaagggtagtcgtggtgattgggagcggagattggcctcccgtacttgtggtgcggttggtcaaaataggagtccccttcggtggacacacggctagtggtggttgtaaagacccttttctgctgtgtgttgtgagctgtttgggaaaccctcaaaaaaccccaatgtgtcgttctaggatgatacttcgaccggtcaggcgggactacccgctgagttt

>MaryvilleTNT10

aaggatcattgtcgaacccatggcagaacgacctgtgaacatgtaaatacaat-gggtgatggggaaatggatcttggttctaacacctcctagcgtgcctgcatgctttctcttttgggctatcatgcatgtattgttggaatttaacaaaaccccggcacggcatgtgccaaggaaaacaaaaaacgagaaggactcgacctgatgccccgtttgtggtgtgcattctgagcgtgtcctcctttgaatcacaaacgactctcggcaacggatatctcggctcacgcatcgatgaagaacgtagcaaaatgcgatacttggtgtgaattgcagaatcccgtgaaccatcgagtttttgaacgcaagttgcgcctgaagccatccggttgagggcacgcctgcctgggcgtcacgcatcgcgttgcccccatacttcccttaagggtagtcgtggtgattgggagcggagattggcctcccgtacttgtggtgcggttggtcaaaataggagtccccttcggtggacacacggctagtggtggttgtaaagacccttttctgctgtgtgttgtgagctgtttgggaaaccctcaaaaaaccccaatgtgtcgttctaggatgatacttcgaccggtcaggcgggactacccgctgagttt

>MaryvilleTNT9

aaggatcattgtcgaacccatggcagaacgacctgtgaacatgtaaatacaat-gggtgatggggaaatggatcttggttctaacacctcctagcgtgcctgcatgctttctcttttgggctatcatgcatgtattgttggaatttaacaaaaccccggcacggcatgtgccaaggaaaacaaaaaacgagaaggactcgacctgatgccccgtttgtggtgtgcattctgagcgtgtcctcctttgaatcacaaacgactctcggcaacggatatctcggctcacgcatcgatgaagaacgtagcaaaatgcgatacttggtgtgaattgcagaatcccgtgaaccatcgagtttttgaacgcaagttgcgcctgaagccatccggttgagggcacgcctgcctgggcgtcacgcatcgcgttgcccccatacttcccttaagggtagtcgtggtgattgggagcggagattggcctcccgtacttgtggtgcggttggtcaaaataggagtccccttcggtggacacacggctagtggtggttgtaaagacccttttctgctgtgtgttgtgagctgtttgggaaaccctcaaaaaaccccaatgtgtcgttctaggatgatacttcgaccggtcaggcgggactacccgctgagttt

>MaryvilleTNT11

aaggatcattgtcgaacccatggcagaacgacctgtgaacatgtaaatacaat-gggtgatggggaaatggatcttggttctaacacctcctagcgtgcctgcatgctttctcttttgggctatcatgcatgtattgttggaatttaacaaaaccccggcacggcatgtgccaaggaaaacaaaaaacgagaaggactcgacctgatgccccgtttgtggtgtgcattctgagcgtgtcctcctttgaatcacaaacgactctcggcaacggatatctcggctcacgcatcgatgaagaacgtagcaaaatgcgatacttggtgtgaattgcagaatcccgtgaaccatcgagtttttgaacgcaagttgcgcctgaagccatccggttgagggcacgcctgcctgggcgtcacgcatcgcgttgcccccatacttcccttaagggtagtcgtggtgattgggagcggagattggcctcccgtacttgtggtgcggttggtcaaaataggagtccccttcggtggacacacggctagtggtggttgtaaagacccttttctgctgtgtgttgtgagctgtttgggaaaccctcaaaaaaccccaatgtgtcgttctaggatgatacttcgaccggtcaggcgggactacccgctgagttt

>MaryvilleTNT2

aaggatcattgtcgaacccatggcagaacgacctgtgaacatgtaaatacaat-gggtgatggggaaatggatcttggttctaacacctcctagcgtgcctgcatgctttctcttttgggctatcatgcatgtattgttggaatttaacaaaaccccggcacggcatgtgccaaggaaaacaaaaaacgagaaggactcgacctgatgccccgtttgtggtgtgcattctgagcgtgtcctcctttgaatcacaaacgactctcggcaacggatatctcggctcacgcatcgatgaagaacgtagcaaaatgcgatacttggtgtgaattgcagaatcccgtgaaccatcgagtttttgaacgcaagttgcgcctgaagccatccggttgagggcacgcctgcctgggcgtcacgcatcgcgttgcccccatacttcccttaagggtagtcgtggtgattgggagcggagattggcctcccgtacttgtggtgcggttggtcaaaataggagtccccttcggtggacacacggctagtggtggttgtaaagacccttttctgctgtgtgttgtgagctgtttgggaaaccctcaaaaaaccccaatgtgtcgttctaggatgatacttcgaccggtcaggcgggactacccgctgagttt

>TofficinaleHerbariumW413345

aaggatcattgtcgaacccatggcagaacgacctgtgaacatgtaaatacaat-gggtgatggggaaatggatcttggttctaacacctcctagcgtgcctgcatgctttctcttttgggctatcatgcatgtattgttggaatttaacaaaaccccggcacggcatgtgccaaggaaaacaaaaaacgagaaggactcgacctgatgccccgtttgtggtgtgcattctgagcgtgtcctcctttgaatcacaaacgactctcggcaacggatatctcggctcacgcatcgatgaagaacgtagcaaaatgcgatacttggtgtgaattgcagaatcccgtgaaccatcgagtttttgaacgcaagttgcgcctgaagccatccggttgagggcacgcctgcctgggcgtcacgcatcgcgttgcccccatacttcccttaagggtagtcgtggtgattgggagcggagattggcctcccgtacttgtggtgcggttggtcaaaataggagtccccttcggtggacacacggctagtggtggttgtaaagacccttttctgctgtgtgttgtgagctgtttgggaaaccctcaaaaaaccccaatgtgtcgttctaggatgatacttcgaccggtcaggcgggactacccgctgagttt

>MaryvilleTNT5

aaggatcattgtcgaacccatggcagaacgacctgtgaacatgtaaatacaattgggtgatggggagatggatcttggttctaacacctcctagcgtgcctgcatgctttctcttttgggctatcatgcatgtattgttggaatttaacaaaaccccggcacggcatgtgccaaggaaaacaataaacgagaaggactcgacctgatgccccgtttgtggtgtgcattctgagcgtgtcctcctttgaatcacaaacgactctcggcaacggatatctcggctcacgcatcgatgaagaacgtagcaaaatgcgatacttggtgtgaattgcagaatcccgtgaaccatcgagtttttgaacgcaagttgcgcctgaagccatccggttgagggcacgcctgcctgggcgtcacgcatcgcgttgcccccatacttcccttaagggtagtcgtggtgattgggagcggagattggcctcccgtacttgtggtgcggttggtcaaaataggagtccccttcggtggacacacggctagtggtggttgtaaagacccttttctgctgtgtgttgtgagctgctagggaaaccctcaaaaaaccccaatgtgtcgttctaggatgatacttcgactggtcaggcgggactacccgctgagttt

>PinedaleALM57

aaggatcattgtcgaacccatggcagaacgacctgtg-acatgtaaatacaattgggtgatggggaaatggatcttggttctaacacctcctagcgtgcctgcatgctttctcttttgggctatcatgcatgtattgttggaatttaacaaaaccccggcacggcatgtgccaaggaaaacaaaaaacgagaaggactcgacctgatgccccgtttgtggtgtgcattctgagcgtgtcctcctttgaatcacaaacgactctcggcaacggatatctcggctcacgcatcgatgaagaacgtagcaaaatgcgatacttggtgtgaattgcagaatcccgtgaaccatcgagtttttgaacgcaagttgcgcctgaagccatccggttgagggcacgcctgcctgggcgtcacgcatcgcgttgcccccatacttcccttaagggtagtcgtggtgattgggagcggagattggcctcccgtacttgtggtgcggttggtcaaaataggagtccccttcggtggacacacggctagtggtggttgtaaagacccttttctgctgtgtgttgtgagctgttagggaaaccctcaaataaccccaatgtgtcgttctaggatgatacttcgaccggtcaggcgggactacccgctgagttt

>Tuscaloosa59SraALM18

aaggatcattgtcgaacccaaggcagaacgacctgtgaacatgtaaatacaactgggtgatggggagatggatcttggttctaacacctcctagcgtgcctgcatgctttctcttttgggctatcatgcatgtattgttggaatttaacaaaaccccggcacggcatgtgccaaggaaaacaataaacgagaaggactcgacctgatgccccgtttgtggtgtgcattctgagcgtgtcctcctttgaatcacaaacgactctcggcaacggatatctcggctcacgcatcgatgaagaacgtagcaaaatgcgatacttggtgtgaattgcagaatcccgtgaaccatcgagtttttgaacgcaagttgcgcccgaagccatccggttgagggcacgcctgcctgggcgtcacgcatcgcgtcgcccccatacttcccttaagggtagtcgtggtgattgggagcggagattggcttcccgtgcttgtggtgcggttggtcaaaataggagtccccttcggtggacacacggctagtggtggttgtaaagacccttttctgctgtgtgttgtgagctgttagggaaaccctcaaataaccccaatgtgtcgttctaggatgatgcttcgactggtcaggcgggactacccgctgagttt

>TofficinaleS2

aaggatcattgtcgaacccatggcagaacgacctgtgaacatgtaaatacaactgggtgatggggagatggatcttgattctaacacctcctagcgtgactgcatgctttctcttttgggctatcatgcatgtattgttggaatttaacaaaaccccggcacggcatgtgccaaggaaaacaataaatgagaaggactcgacctgatgccccgtttgtggtgtgcattctgagcgtgtcctcctttgaatcacaaacgactctcggcaacggatatctcggctcacgcatcgatgaagaacgtagcaaaatgcgatacttggtgtgaattgcagaatcccgtgaaccatcgagtttttgaacgcaagttgcgcccgaagccatccggttgagggcacgcctgcctgggcgtcacgcatcgcgtcgcccccatacttcccttaagggtagtcgtggtgattgggagcggagattggcttcccgtgcttgtggtgcggttggtcaaaataggagtccccttcggtggacacatggctagtggtggttgtaaagacccttttctgctgtgtgttgtgagctgttagggaaaccctcaaaaaaacccaatgtatcgttctaggacgatgc-tcgaccggtcaggcgggactacccgctgagttt

>Tuscaloosa59SraALM59

aaggatcattgtcgaacccatggcag-acgacctgtgaacatgtaaatacaatcgggtgatggggagatggatcttggttctaacacctcctagcgtgcctgcatgctttctcttttgggctatcatgcatgtattgttggaatttaacaaaaccccggcacggcatgtgccaaggaaaacaataaacgagaaggactcgacctgatgccccgtttgtggtgtgcattctgagcgtgtcctcctttgaatcacaaacgactctcggcaacggatatctcggctcacgcatcgatgaagaacgtagcaaaatgcgatacttggtgtgaattgcagaatcccgtgaaccatcgagtttttgaacgcaagttgcgcctgaagccatccggttgagggcacgcctgcctgggcgtcacgcatcgcgtcgcccccatacttcccttaagggtagtcgtggtgattgggagcggagattggcttcccgtacttgtggtgcggttggtcaaaataggagtccccttcggtggacacacggctagtggtggttgtaaagacccttttctgctgtgtgttgtgagctgctagggaaaccctcaaaaaaccccaatgtgtcgttctaggatgatacttcgactggtcaggcgggactacccgctgagttt

>Youngia02

aaggatcattgtcgaacccaaggcagaacgacctgtgaacacgtaaatacaactgggtgatggggagatggatcttggttctaacrcctcctagcgtgcctgcatgctttctcttttgggctatcatgcatgtattgttggaatttaacaaaaccccggcacggcatgtgccaaggaaaacaataaacgagaaggactcgacctgatgccccgtttgtggtgtgcattctgagcgtggcctccttggaatcataaacgactctcggcaacggatatctcggctcacgcatcgatgaagaacgtagcaaaatgcgatacttggtgtgaattgcagaatcccgtgaaccatcgagtttttgaacgcaagttgcgcccaatgccatttggttgagggcacgcctgcctgggcgtcacgcatcgcgtcgcccccatacttccttaaccggtacatctggtg-ctgggggcggagattggcctcccatgcctgtggtgtggctggcctaaagagttgtcccatttggtggacacacggttagtggtggttgtatagaccctcgtctcccgtgtgtcgttagcctctagggacatatt--gaaaaccctactgtattgtctctagatgatacttcgactggtcaggcgggactacccgctgagttt

>Youngia03

a----------------------------------------------------------------------------------------------------------------------------------------------------------------------------------------------------------------------------------------cgtggcctccttggaatcataaacgactctcggcaacggatatctcggctcacgcatcgatgaagaacgtagcaaaatgcgatacttggtgtgaattgcagaatcccgtgaaccatcgagtttttgaacgcaagttgcgcccaatgccatttggttgagggcacgcctgcctgggcgtcacgcatcgcgtcgcccccatacttccttaaccggtacatctggtg-ctgggggcggagattggcctcccatgcctgtggtgtggctggcctaaagagttgtcccatttggtggacacacggttagtggtggttgtatagaccctcgtctcccgtgtgtcgttagcctctagggacatatt--gaaaaccctactgtattgtctctagatgatacttcgactggtcaggcgggactacccgctgagttt

>Youngia04

a----------------------------------------------------------------------------------------------------------------------------------------------------------------------------------------------------------------------------------------cgtggcctccttggaatcataaacgactctcggcaacggatatctcggctcacgcatcgatgaagaacgtagcaaaatgcgatacttggtgtgaattgcagaatcccgtgaaccatcgagtttttgaacgcaagttgcgcccaatgccatttggttgagggcacgcctgcctgggcgtcacgcatcgcgtcgcccccatacttccttaaccggtacatctggtg-ctgggggcggagattggcctcccatgcctgtggtgtggctggcctaaagagttgtcccatttggtggacacacggttagtggtggttgtatagaccctcgtctcccgtgtgtcgttagcctctagggacatatt--gaaaaccctactgtattgtctctagatgatacttcgactggtcaggcgggactacccgctgagttt

>Youngia05

a----------------------------------------------------------------------------------------------------------------------------------------------------------------------------------------------------------------------------------------cgtggcctccttggaatcataaacgactctcggcaacggatatctcggctcacgcatcgatgaagaacgtagcaaaatgcgatacttggtgtgaattgcagaatcccgtgaaccatcgagtttttgaacgcaagttgcgcccaatgccatttggttgagggcacgcctgcctgggcgtcacgcatcgcgtcgcccccatacttccttaaccggtacatctggtg-ctgggggcggagattggcctcccatgcctgtggtgtggctggcctaaagagttgtcccatttggtggacacacggttagtggtggttgtatagaccctcgtctcccgtgtgtcgttagcctctagggacatatt--gaaaaccctactgtattgtctctagatgatacttcgactggtcaggcgggactacccgctgagttt

>TbrevicorniculatumS6

aaggatcattgttggaacccaggcaggacgacctgtg-acacgtaaatacaactgggtgatggggagatggatcttggttctaacacctcctagcgtgcctgcatgctttctcttttgggctatcatgcatgtattgttggaatttaacaaaaccccggcacggcatgtgccaaggaaaacaataaacgagaaggacttgacctgatgccccgtttgtggtgtgcattctgagcgtgtccttctttgaatcacaaacgactctcggcaacggatatcttggctcacgcatcgatgaagaacgtagcaaaatgcgatacttggtgtgaattgcagaatcccgtgaaccatcgagtttttgaacgcaagttgcgcccgaagccatccggttcagggcacgcctgcctgggcgtcacgcatcgcgtcgcccccatacttcccttaagggtagtcgtggtgattgggagcggagattggcctcccgtgcttgttgtgcggttggtcaaaataggagtccccttcggtggacacacggctagtggtggttgtaaagacccttttctgctgtgtgttgtgagctgctagggaaaccctcataaaaacccaatgtatcgtcctaggatgatgcttcgaccggccaggcggaactaccggttgagttt

>KnoxvilleTN55

aaggatcattgttggacccaaggcaggacgacctgtggacacgtaaatacaactgggtgatggggagatggatcttggttctaacaccttctagcgtgcctgcatgctttctcttttgggctatcatgcatgtattgttggaatttaacaaaaccccggcacggcatgtgccaaggaaaacaataaacgagaaggacttgacctgatgccccgtttgtggtgtgcattctgagcgtgtccttctttgaatcacaaacgactctcggcaacggatatcttggctcacgcatcgatgaagaacgtagcaaaatgcgatacttggtgtgaattgcagaatcccgtgaaccatcgagtttttgaacgcaagttgcgcccgaagccatccggttcagggcacgcctgcctgggcgtcacgcatcgcgtcgcccccatacttcccttaagggtagtcgtggtgattgggagcggagattggcctcccgtgcttgttgtgcggttggtccaaataggagtccccttcggtggacacacggctagtggtggttgtaaagacccttttctgctgtgtgttgtgagctgctagggaaaccctcataaaaacccaatgtatcgtcctaggatgatgcttcgaccggtcaggcgg-----------------

>TbrevicorniculatumS5

--------------gacccacggcagaacgacctgtgaacacgtaaatacaactgggtgatggggagatggatcttggttctaacacctcctagcgtgcctgcatgctttctcttttgggctatcatgcatgtattgttggaatttaacaaaaccccggcacggcatgtgccaaggaaaacaataaacgagaaggacttgacctgatgccccgtttgtggtgtgcattctgagcgtgtccttctttgaaacacaaacgactctcggcaacggatatcttggctcacgcatcgatgaagaacgtagcaaaatgcgatacttggtgtgaattgcagaatcccgtgaaccatcgagtttttgaacgcaagttgcgcccgaagccatccggttcagggcacgcctgcctgggcgtcacgcatcgcgtcgcccccatacttcccttaagggtagtcgtggtgattgggagcggagattggcctcccgtgcttgttgtgcggttggtcaaaataggagtccccttcggtggacacacggctagtggtggttgtaaagacccttttctggtgtgtgttgtgagctgctagggaaaccgtcataaaaccccaatgtatcgttctaggatgatgcttcgaccggtcaggcggg----------------

>TbrevicorniculatumS8

aagattcattgtcg-atgcaaggcag-acgacctgtgaacacgtaaatacaactgggtgatggggagatggatcttggttctaacacctcctagcgtgcctgcatgctttctcttttgggctatcatgcatgtattgttggaatttaacaaaaccccggcacggcatgtgccaaggaaaacaataaacgagaaggacttgacctgatgccccgtttgtggtgtgcattctgagcgtgtccttctttgaatcacaaacgactctcggcaacggatatcttggctcacgcatcgatgaagaacgtagcaaaatgcgatacttggtgtgaattgcagaatcccgtgaaccatcgagtttttgaacgcaagttgcgcccgaagccatccggttcagggcacgcctgcctgggcgtcacgcatcgcgtcgcccccatactttccttaagggtagtcgtggtgattgggagcggagattggccttctgtgcttgttgtgcggttggtcaaaataggagtccccttcggtggacacacggctagtggtggttgtaaagacccttttctgctgtgtgttgtgagctgctagggaaaccctcataaaaacccaatgtatcgtcctaggatgatgcttcgaccggtcaggcggaactacccgttgagttt

>PinedaleALM37

cag------------------------acgacctgtg-acatgtaaatacaattgggtgatggggagatggatcttggttctaacacctcctagcgtgcctgcatgcttttctttttgggctatcatgcatgtattgttggaatttaacaaaaccccggcacggcatgtgccaaggaaaacaaaaaacgagaaggactcgacctgatgccccgtttgtggtgtgcattctgagcgtgtcctcctttgaatcacaaacgactctcggcaacggatatctcggctcacgcatcgatgaagaacgtagcaaaatgcgatacttggtgtgaattgcagaatcccgtgaaccatcgagtttttgaacgcaagttgcgcctgaagccatccggttgagggcacgcctgcctgggcgtcacgcatcgcgttgcccccatacttcccttaagggtagtcgtggtgattgggagcggagattggcctcccgtacttgtggtgcggttggtcaaaataggagtccccttcggtggacacacggctagtggtggttgtaaagacccttttctgctgtgtgttgtgagctgtttgggaaaccctcaaaaaaccccaatgtgtcgttctaggatgatacttcgaccggtcaggcgggactacccgctgagttt

>KF437406_TKS_Ceratoidea

----------------------------------------------------------------------------------------------------------------------ggctatcatgcatgtattgttgggcattaacaaaaccccggcacggcatgtgccaaggaaaataataaacgagaaggacacgacttgatgccccgtttgcggtgtgcattccgagcgtgtcctcctttgaatcacaaacgactctcggcaacggatatctcggctcacgcatcgatgaagaacgtagcaaaatgcgatacttggtgtgaattgcagaatcccgtgaaccatcgagtttttgaacgcaagttgcgcctgaagccatccggttgagggcacgcctgcctgggcgtcacgcatcgcgtcgcccccatacttctcttaagggtagttttggtgattgggagcggagattggcctcctgtgcttgttgtgcggttggtccaaataggagtccccttcggtggacacacggctagtggtggttgtaaagacccttttctgttgtgtgtcgtgagctgc-waggaaaccctcaaaaaaccccattgtattgtcctaggatgatgc----------------------------------

>KF437407_TKS_Ceratoidea

----------------------------------------------------------------------------------------------------------------------ggctatcatgcatgtattgttgggcattaacaaaaccccggcacggcatgtgccaaggaaaataataaacgagaaggacacgacttgatgccccgtttgcggtgtgcattccgagcgtgtcctcctttgaatcacaaacgactctcggcaacggatatctcggctcacgcatcgatgaagaacgtagcaaaatgcgatacttggtgtgaattgcagaatcccgtgaaccatcgagtttttgaacgcaagttgcgcctgaagccatccggttgagggcacgcctgcctgggcgtcacgcatcgcgtcgcccccatacttctcttaagggtagttttggtgattgggagcggagattggcctcctgtgcttgttgtgcggttggtccaaataggagtccccttcggtggacacacggctagtggtggttgtaaagacccttttctgttgtgtgtcgtgagctgc-aaggaaaccctcaaaaaaccccattgtattgtcctaggat---------------------------------------

>TKSherbMontnew

aaggatcattgtcgaacccacggcagaacgacctgtgaacacgtaaatacaaccgggtgatggggagatggatcttggttttaacacctcccaacgtgcctgcgtgctttctcttttgggctatcatgcatgtattgttgggcattaacaaaaccccggcacggcatgtgccaaggaaaataataaacgagaaggacacgacttgatgccccgtttgcggtgtgcattccgagcgtgtcctcctttgaatcacaaacgactctcggcaacggatatctcggctcacgcatcgatgaagaacgtagcaaaatgcgatacttggtgtgaattgcagaatcccgtgaaccatcgagtttttgaacgcaagttgcgcctgaagccatccggttgagggcacgcctgcctgggcgtcacgcatcgcgtcgcccccatacttctcttaagggtagttttggtgattgggagcggagattggcctcctgtgcttgttgtgcggttggtccaaataggagtccccttcggtggacacacggctagtggtggttgtaaagacccttttctgttgtgtgtcgtgagctgc-aaggaaaccctcaaaaaaccccattgtattgtcctaggatgatgc-tcgaccggtca----------------------

>TKShebrKent

aaggatcattgtcgaacccacggcagaacgacctgtgaacacgtaaatacaaccgggtgatggggagatggatcttggttttaacacctcccaacgtgcctgcgtgctttctcttttgggctatcatgcatgtattgttgggcattaacaaaaccccggcacggcatgtgccaaggaaaataataaacgagaaggacacgacttgatgccccgtttgcggtgtgcattccgagcgtgtcctcctttgaatcacaaacgactctcggcaacggatatctcggctcacgcatcgatgaagaacgtagcaaaatgcgatacttggtgtgaattgcagaatcccgtgaaccatcgagtttttgaacgcaagttgcgcctgaagccatccggttgagggcacgcctgcctgggcgtcacgcatcgcgtcgcccccatacttctcttaagggtagttttggtgattgggagcggagattggcctcctgtgcttgttgtgcggttggtccaaataggagtccccttcggtggacacacggctagtggtggttgtaaagacccttttctgttgtgtgtcgtgagctgc-aaggaaaccctcaaaaaaccccattgtattgtcctaggatgatgcttcgacc-gtcaggcgg-----------------

>TKSK2

aaggatcattgtcgaacccacggcagaacgacctgtgaacacgtaaatacaaccgggtgatggggagatggatcttggttttaacacctcccaacgtgcctgcgtgctttctcttttgggctatcatgcatgtattgttgggcattaacaaaaccccggcacggcatgtgccaaggaaaataataaacgagaaggacacgacttgatgccccgtttgcggtgtgcattccgagcgtgtcctcctttgaatcacaaacgactctcggcaacggatatctcggctcacgcatcgatgaagaacgtagcaaaatgcgatacttggtgtgaattgcagaatcccgtgaaccatcgagtttttgaacgcaagttgcgcctgaagccatccggttgagggcacgcctgcctgggcgtcacgcatcgcgtcgcccccatacttctcttaagggtagttttggtgattgggagcggagattggcctcctgtgcttgttgtgcggttggtccaaataggagtccccttcggtggacacacggctagtggtggttgtaaagacccttttctgttgtgtgtcgtgagctgc-aaggaaaccctcaaaaaaccccattgtattgtcctaggatgatgcttcgacc-gtcaggcgg-----------------

>TKSA1seq

aaggatcattgtcgaacccacggcagaacgacctgtgaacacgtaaatacaaccgggtgatggggagatggatcttggttttaacacctcccaacgtgcctgcgtgctttctcttttgggctatcatgcatgtattgttgggcattaacaaaaccccggcacggcatgtgccaaggaaaataataaacgagaaggacacgacttgatgccccgtttgcggtgtgcattccgagcgtgtcctcctttgaatcacaaacgactctcggcaacggatatctcggctcacgcatcgatgaagaacgtagcaaaatgcgatacttggtgtgaattgcagaatcccgtgaaccatcgagtttttgaacgcaagttgcgcctgaagccatccggttgagggcacgcctgcctgggcgtcacgcatcgcgtcgcccccatacttctcttaagggtagttttggtgattgggagcggagattggcctcctgtgcttgttgtgcggttggtccaaataggagtccccttcggtggacacacggctagtggtggttgtaaagacccttttctgttgtgtgtcgtgagctgc-aaggaaaccctcaaaaaaccccattgtattgtcctaggatgatgcttcgaccggtcaggcgggactacccgctgagttt

>TKSB2

aaggatcattgtcgaacccacggcagaacgacctgtgaacacgtaaatacaaccgggtgatggggagatggatcttggttttaacacctcccaacgtgcctgcgtgctttctcttttgggctatcatgcatgtattgttgggcattaacaaaaccccggcacggcatgtgccaaggaaaataataaacgagaaggacacgacttgatgccccgtttgcggtgtgcattccgagcgtgtcctcctttgaatcacaaacgactctcggcaacggatatctcggctcacgcatcgatgaagaacgtagcaaaatgcgatacttggtgtgaattgcagaatcccgtgaaccatcgagtttttgaacgcaagttgcgcctgaagccatccggttgagggcacgcctgcctgggcgtcacgcatcgcgtcgcccccatacttctcttaagggtagttttggtgattgggagcggagattggcctcctgtgcttgttgtgcggttggtccaaataggagtccccttcggtggacacacggctagtggtggttgtaaagacccttttctgttgtgtgtcgtgagctgc-aaggaaaccctcaaaaaaccccattgtattgtcctaggatgatgcttcgaccggtcaggcgggactacccgctgagttt

>TbrevicorniculatumS7

aaggatcattgtcgaacccacggcagaacgacctgtgaacacgtaaatacaaccgggtgatggggagatggatcttggttttaacacctcccaacgtgcctgcgtgctttctcttttgggctatcatgcatgtattgttgggcattaacaaaaccccggcacggcatgtgccaaggaaaataataaacgagaaggacacgacttgatgccccgtttgcggtgtgcattccgagcgtgtcctcctttgaatcacaaacgactctcggcaacggatatctcggctcacgcatcgatgaagaacgtagcaaaatgcgatacttggtgtgaattgcagaatcccgtgaaccatcgagtttttgaacgcaagttgcgcctgaagccatccggttgagggcacgcctgcctgggcgtcacgcatcgcgtcgcccccatacttctcttaagggtagttttggtgattgggagcggagattggcctcctgtgcttgttgtgcggttggtccaaataggagtccccttcggtggacacacggctagtggtggttgtaaagacccttttctgttgtgtgtcgtgagctgc-aaggaaaccctcaaaaaaccccattgtattgtcctaggatgatgcttcgaccggtcaggcgggactacccgctgagttt

>TKS3

aaggatcattgtcgaacccacggcagaacgacctgtgaacacgtaaatacaaccgggtgatggggagatggatcttggttttaacacctcccaacgtgcctgcgtgctttctcttttgggctatcatgcatgtattgttgggcattaacaaaaccccggcacggcatgtgccaaggaaaataataaacgagaaggacacgacttgatgccccgtttgcggtgtgcattccgagcgtgtcctcctttgaatcacaaacgactctcggcaacggatatctcggctcacgcatcgatgaagaacgtagcaaaatgcgatacttggtgtgaattgcagaatcccgtgaaccatcgagtttttgaacgcaagttgcgcctgaagccatccggttgagggcacgcctgcctgggcgtcacgcatcgcgtcgcccccatacttctcttaagggtagttttggtgattgggagcggagattggcctcctgtgcttgttgtgcggttggtccaaataggagtccccttcggtggacacacggctagtggtggttgtaaagacccttttctgttgtgtgtcgtgagctgc-aaggaaaccctcaaaaaaccccattgtattgtcctaggatgatgcttcgaccggtcaggcgggactacccgctgagttt

>TKS04

aaggatcattgtcgaacccacggcagaacgacctgtgaacacgtaaatacaaccgggtgatggggagatggatcttggttttaacacctcccaacgtgcctgcgtgctttctcttttgggctatcatgcatgtattgttgggcattaacaaaaccccggcacggcatgtgccaaggaaaataataaacgagaaggacacgacttgatgccccgtttgcggtgtgcattccgagcgtgtcctcctttgaatcacaaacgactctcggcaacggatatctcggctcacgcatcgatgaagaacgtagcaaaatgcgatacttggtgtgaattgcagaatcccgtgaaccatcgagtttttgaacgcaagttgcgcctgaagccatccggttgagggcacgcctgcctgggcgtcacgcatcgcgtcgcccccatacttctcttaagggtagttttggtgattgggagcggagattggcctcctgtgcttgttgtgcggttggtccaaataggagtccccttcggtggacacacggctagtggtggttgtaaagacccttttctgttgtgtgtcgtgagctgc-aaggaaaccctcaaaaaaccccattgtattgtcctaggatgatgcttccaccggtcaggcgggactacccgctgagttt

>TofficinaleNCBIconsensus

tg----------cgaacccaaggcagaacgacctgtgaacacgtaaatacaactgggtgatggggagatggatcttggttctaacacctcctagcgtgcctgcatgctttctcttttgggctatcatgcatgtattgttggaatttaacaaaaccccggcacggcatgtgccaaggaaaacaataaacgagaaggactcgacctgatgccccgtttgtggtgtgcattctgagcgtgtcctcctttgaatcacaaacgactctcggcaacggatatctcggctcacgcatcgatgaagaacgtagcaaaatgcgatacttggtgtgaattgcagaatcccgtgaaccatcgagtttttgaacgcaagttgcgcccgaagccatccggttcagggcacgcctgcctgggcgtcacgcatcgcgtcgcccccatacttcccttaagggtagtcgtggtgattgggagcggagattggcttcccgtgcttgttgtgcggttggtcaaaataggagtccccttcggtggacacacggctagtggtggttgtaaagacccttttctgctgtgtgttgtgagctgctagggaaaccctcaaaaaaacccaatgtatcgttctaggacgatgcttcgacc---------------------------

>MaryvilleTNT8

aaggatcattgtcgaaccaatggcagaacgacctgtgaacatgtaaatacaatcgggtgatggggaaatggatcttggttctaacacctcctagcgtgcctgcatgctttctcttttgggctatcatgcatgtattgttggaatttaacaaaaccccggcacggcatgtgccaaggaaaacaaaaaacgagaaggactcgacctgatgccccgtttgtggtgtgcattctgagcgtgtcctcctttgaatcacaaacgactctcggcaacggatatctcggctcacgcatcgatgaagaacgtagcaaaatgcgatacttggtgtgaattgcagaatcccgtgaaccatcgagtttttgaacgcaagttgcgcctgaagccatccggttgagggcacgcctgcctgggcgtcacgcatcgcgttgcccccatacttcccttaagggtagtcgtggtgattgggagcggagattggcctcccgtacttgtggtgcggttggtcaaaataggagtccccttcggtggacacacggctagtggtggttgtaaagacccttttctgctgtgtgttgtgagctgtttgggaaaccctcaaaaaactacactgtatcat-------------------------------atcctccgcttattga

>TerythrospermumNCBIconsensus

-----------tcgaacccaaggcagaacgacctgtgaacacgtaaatacaantgggtgatggggagatggatcttggttctaacacctcctagcgtgcctgcatgctttctcttttgggctatcatgcatgtattgttggaatttaacaaaaccccggcacggcatgtgccaaggaaaacaataaacnagaaggactcgacctgatgccccgtttgtggtgtgcattctgagcgtgtcctcctttgaatcacaaacgactctcggcaacggatatctcggctcacgcatcgatgaagaacgtagcaaaatgcgatacttggtgtgaattgcagaatcccgtgaaccatcgagtttttgaacgcaagttgcgcccgaagccatccggttnagggcacgcctgcctgggcgtcacgcatcgcgtngcccccatacttcccttaagggtagtcgtggtgattgggagcggagattggcntcccgtgcttgtngtgcggttggtcaaaataggagtccccttcggtggacacacggctagtggtggttgtaaagacccttttctgctgtgtgttgtgagctgttngggaaaccctcaaaaaaccccaatgtatcgttctaggatgatgcttcgaccggtcaggcgggactacccgctgagttt

>MaryvilleTNT3

aaggatcattgttagaacccaggcagaatgacttgtgaacacgtaattactattgggagatggggagctgcatcttggttctaacacctcctagcgtgcctgcatgctttctcttttgggctatcatgcatgtattgttggaatttaacaaaaccccggcacggcatgtgccaaggaaaacaataaacgagaaggactcgacctgatgccccgtttgtggtgtgcattctgagcgtgtcctcctttgaatcacaaacgactctcggcaacggatatctcggctcacgcatcgatgaagaacgtagcaaaatgcgatacttggtgtgaattgcagaatcccgtgaaccatcgagtttttgaacgcaagttgcgcccgaagccatccggttcagggcacgcctgcctgggcgtcacgcatcgcgtcgcccccatacttcccttaagggtagtcgtggtgattgggagcggagattggcttcccgtgcttgttgtgcggttggtcaaaataggagtcccctttggtggacacacggctagtggtggttgtaaagacccttttatgttgtgtgttgtgagttgctagggaaaccctcaaaaaaaaccaacggatca--------------------------------attctccgct------

>Youngia01

aaggatcattgtcgaacc-atggcagaatgacccgtgaacatgtaaatactactgggtgatggggagacggtccttggtcttaatgcctcccgacttgtgtgcatggtgtcttgtacaggccaccatgtatgtctcgttggaccaataacaaaccccggcacggaatgtgccaaggaaatgaaaaaatgagaaggacttgtcctgatgccccgtatgcggtgtgcttacaggtcgtggcctccttggaatcataaacgactctcggcaacggatatctcggctcacgcatcgatgaagaacgtagcaaaatgcgatacttggtgtgaattgcagaatcccgtgaaccatcgagtttttgaacgcaagttgcgcccaatgccatttggttgagggcacgcctgcctgggcgtcacgcatcgcgtcgcccccatacttccttaaccggtacatctggtg-ctgggggcggagattggcctcccatgcctgtggtgtggctggcctaaagagttgtcccatttggtggacacacggttagtggtggttgtatagaccctcgtctcccgtgtgtcgttagcctctagggacatatt--gaaaaccctactgtattgtctctagatganacttcgact---------------------------

>KnoxvilleTNK1

-aggatcattgtcgaacccaaggcagaacgacctgtgaacacgtaaatacaactgggtgatggggagatggatcttggttctaacaccttctagcgtgcctgcatgctttctcttttgggctatcatgcatgtattgttggaatttaacaaaaccccggcacggcatgtgccaaggaaaacaataaacgagaaggactcgacctgatgccccgtttgtggtgtgcattctgagcgtgtcctcctttgaatcacaaacgactctcggcaacggatatctcggctcacgcatcgatgaagaacgtagcaaaatgcgatacttgggtgaaattgcagaatcccgtgaaccatcgagtttttgaacgcaagttgcgcccgaagccatccggttcaggggccacttgcctgggcgtcacgcatcgcgccccccccatactttccttaagggt---------------------------------------------------------------------------------------------------------------------------------------------------------------------------------------------------------------------

>Lactuca01

aaggatcattgtcgaaccc-agacagtacgacctgtgaacatgttaacacaacggggcgacagggaaacgggccttggtcctaacccctcccgacgtgcgtttgtgatgccttctattgggcatcatggat--cccgtcggaccataac-aaaccccggcacggcatgtgccaaggaaaacaaaaa-tgagaaggacacgtcttgatgccccgtttgcggtgtgcatgcgtttcgtggcctcctagaaactacaaacgactctcggcaacggatatctcggctcacgcatcgatgaagaacgtagcaaaatgcgatacttggtgtgaattgcagaatcccgtgaaccatcgagtttttgaacgcaagttgcgcctgaagccatccggccgaaggcacgcctgcctgggcgtcacgcatcgcgtcgctcccatactcctccaatgggttgtgatggtg-ttaggggcggataatggcctcccgtgcttgtgtttcggttggcctaaaaaggagttcccttcggcggacacacaactagtggtggttgaacatacccttg--tgttgtgcgtcgtgagctgtgagggaagccctcatctaaccccattgtatcgtctttggacggtgcttcgaccggtcaggcgggactacccgctgagttt

>Hypochaeris01

aaggatcattgtcgaacccaaggcagaacgacccgtgaacatgtaaatacaaccgggcgatggggagactggcattggtccgatcctctcctggcntgcgtttgtggtgcctcgtatggggtgccatagatgacatgctggaccataacaaaaacccggcacggcatgtgccaaggaaaacaaaaattgagatggactcgtcttgttgccccgtttgcggtgtgcatgcagttcgtggcctctttgtaattacaaacgactctcggcaacggatatctcggctcacgcatcgatgaagaacgtagcaaaatgcgatacttggtgtgaattgcagaatcccgtgaaccatcgagtttttgaacgcaagttgcgcccgaagccatccggtcgagggcacgcctgcctgggcgtcacgcatcgcgtcgccccatatctactct------------cggtg-atgggagcggagattggtctcccgtacttg-gttgcggttggcctaaaaaggagtcccctacagcggacacacgactagtggtggttgaatagaccctcgt-tatcgtgtgtcgtgagttgtttgggaagccctcaactaacccgattgtatcgttttag------------------------------------------

>Hypochaeris02

aaggatcattgtcgaacccaaggcagaacgacccgtgaacatgtaaatacaaccgggcgatggggagactggcattggtccgatcctctcctggcgtgcgtttgtggtgcctcgtatggggtgccatagattacatgctggaccataacaaaaacccggcacggcatgtgccaaggaaaacaaaaattgagatggactcgtcttgttgccccgttcgcggtgtgcatgcagttcgtggcctctttgtaattaccaacgactctcggcaacggatatctcggctcacgcatcgatgaagaacgtagcaaaatgcgatacttggtgtgaattgcagaatcccgtgaaccatcgagtttttgaacgcaagttgcgcccgaagccatccggtcgagggcacgcctgcctgggcgtcacgcatcgcgtcgccccatatctactct------------tggtg-atgggagcggagattggtcttccgtacttg-gttgcggttggcctaaaaaggagtcccctacagcggacacacgactagtggtggttgaatagaccctcgt-tatcgtgtgtcgtgagctgtttgggaagccctcaactaacccgattgtatcgttttaggacggtgcttcgaccggtcaggcgggactacccgctgagttt

>Hypochaeris58

aaggatcattgtcgaacccaaggcagaacgacccgtggacatgttaatacaaccgggcgatggggagactggcattggtccgatcctctcctggcgtgcgtttgtggtgcctcgtatggggtgccatagattacatgctggaccataacaaaaacccggcacggcatgtgccaaggaaaacaaaaattgagatggactcgtcttgttgccccgttcgcggtgtgcatgcagttcgtggcctctttgtaattacaaacgactctcggcaacggatatctcggctcacgcatcgatgaagaacgtagcaaaatgcgatacttggtgtgaattgcagaatcccgtgaaccatcgagtttttgaacgcaagttgcgcccgaagccatccggtcgagggcacgcctgcctgggcgtcacgcatcgcgtcgccccatatctactct------------tggtg-atgggagcggagattggtctcccgtacttg-gttgcggttggcctaaaaaggagtcccctacagcggacacacgactagtggtgggtgaatagaccctcgt-tatcgtgtgtcgtgagctgtttgggaagccctcaactaacccgattgtatcgttttaggacggtgcttcgaccggtcaggggggactacccgctgagttt

>Hypochaeris17

aaggatcattgtcgaacccaaggcagaacgacccgtgaacatgtaaatacaaccgggcgatggggagactggcattggtccgatcctctcctggcgtgcgtttgtggtgcctcgtatggggtgccatagattacatgctggaccataacaaaaacccggcacggcatgtgccaaggaaaacaaaaattgagatggactcgtcttgttgccccgttcgcggtgtgcatgcagttcgtggcctctttgtaattacaaacgactctcggcaacggatatctcggctcacgcatcgatgaagaacgtagcaaaatgcgatacttggtgtgaattgcagaatcccgtgaaccatcgagtttttgaacgcaagttgcgcccgaagccatccggtcgagggcacgcctgcctgggcgtcacgcatcgcgtcgccccatatctactct------------tggtg-atgggagcggagattggtctcccgtacttg-gttgcggttggcctaaaaaggagtcccctacagcggacacacgactagtggtggttgaatagaccctcgt-tatcgtgtgtcgtgagctgtttgggaagccctcaactaacccgattgtatcgttttaggacggtgcttcgaccggtcaggcgggactacccgctgagttt

>Hypochaeris14

aaggatcattgtcgaacccaaggcagaacgacccgtgaacatgtaaatacaaccgggcgatggggagactggcattggtccgatcctttcctggcgtgcgtttgtggtgccttgtatggggtgccatagattacatgctggaccataacaaaaacccggcacggcatgtgccaaggaaaacaaaaattgagatggacttgtcttgttgccccgttcgcggtgtgcatgcagttcgtggcctctttgtaattacaaacgactctcggcaacggatatctcggctcacgcatcgatgaagaacgtagcaaaatgcgatacttggtgtgaattgcagaatcccgtgaaccatcgagtttttgaacgcaagttgcgcccgaagccatccggtcgagggcacgcctgcctgggcgtcacgcatcgcgtcgccccatatctactct------------tggtg-atgggagcggagattggtctcccgtacttg-gttgcggttggcctaaaaaggagtcccctacagcggacacacgactagtggtggttgaatagaccctcgt-tatcgtgtgtcgtgagctgtttgggaagccctcaactaacccgattgtatcgttttaggacggtgcttcgaccggccaggcgggactacccgctgagttt

>Hypochaeris46

aaggatcattgtcgaacccaaggcagaacgacccgtgaacatgtagatacaactgggtgatggggagactggcattggtcctatcctctactggcatgcgtttatggtgccttgtatggggtgccatagatgacatgctggaccataacaaaaacccggcacggcatgtgccaaggaaaacaaaaattgagatggactcgtcttgttgccccgttcgcggtgtgcatgcagttcgtggcctctttgtaattacaaacgactctcggcaacggatatctcggctcacgcatcgatgaagaacgtagcaaaatgcgatacttggtgtgaattgcagaatcccgtgaaccatcgagtttttgaacgcaagttgcgcccgaagccatccggtcgagggcacgtctgcctgggcgtcacgcatcgcgtcgccccatatctactct------------tggtg-atgggagcggagattggtctcccgtacttg-gttgcggttggcctaaaaaggagtcccctacagcggacacacgactagtggtggttgaatagaccctcgt-tatcgtgtgtcgtgagctgtttgggaagccctcaactaacccgattgtatcgttttggaacggtgcttcgaccggtcaggcgggactacccgctgagttt

>Hypochaeris49

aaggatcattgtcgaacccaaggcagaacgacccgtgaacatgtagatacaactgggtgatggggagactggcattggtcctatcctctactggcatgcgtttatggtgccttgtatggggtgccatagatgacatgctggaccataacaaaaacccggcacggcatgtgccaaggaaaacaaaaattgagatggactcgtcttgttgccccgttcgcggtgtgcatgcagttcgtggcctctttgtaattacaaacgactctcggcaacggatatctcggctcacgcatcgatgaagaacgtagcaaaatgcgatacttggtgtgaattgcagaatcccgtgaaccatcgagtttttgaacgcaagttgcgcccgaagccatccggtcgagggcacgtctgcctgggcgtcacgcatcgcgtcgccccatatctactct------------tggtg-atgggagcggagattggtctcccgtacttg-gttgcggttggcctaaaaaggagtcccctacagcggacacacgactagtggtggttgaatagaccctcgt-tatcgtgtgtcgtgagctgtttgggaagccctcaactaacccgattgtatcgttttggaacggtgcttcgaccggtcaggcgggactacccgctgagttt

>Hypochaeris51

aaggatcattgtcgaacccaaggcagaacgacccgtgaacatgtaaatacaaccgggcgatggggagactggcattggtccgatcctctcctggcrtgcgtttgtggtgcctcgtatggggtgccatagatgacatgctggaccataacaaaaacccggcacggcatgtgccaaggaaaacaaaaattgagatggactcgtcttgttgccccgtttgcggtgtgcatgcagttcgtggcctctttgtaattacaaacgactctcggcaacggatatctcggctcacgcatcgatgaagaacgtagcaaaatgcgatacttggtgtgaattgcagaatcccgtgaaccatcgagtttttgaacgcaagttgcgcccgaagccatccggtcgagggcacgcctgcctgggcgtcacgcatcgcgtcgccccatatctactct------------cggtg-atgggagcggagattggtctcccgtacttg-gttgcggttggcctaaaaaggagtcccctacagcggacacacgactagtggtggttgaatagaccctcgt-tatcgtgtgtcgtgagttgtttgggaagccctcaactaacccgattgtatcgttttagaacggtgcttcgaccggtataggcggaattaccgttaagtt-

>Hypochaeris10

aaggatcattgtcgaacccatggcagaacgaccagttaacacgtaaatacaactgggtgatggggagatgggccttggctctatcccctctcggtgtgtgtttgtgatgcctcttttggggcgccactgacgtcatgctgaaccttaac-aaaccccggcacggcatgtgccaaggaaaac-aaaatcgagaaggacgcgtcttgttgccccgttcgcggtgtgcatgcgggccgtggcctttttataattacaaacgactctcggcaacggatatctcggctcacgcatcgatgaagaacgtagcaaaatgcgatacttggtgtgaattgcagaatcccgtgaaccatcgagtttttgaacgcaagttgcgcccgaagccatccggccgagggcacgcctgcctgggcgtcacgcatcgcgtcgccccaaatttccaaa------------tctgg-ttgggggcggagattggcctcccgtacct--gttgtggttggcctaaaaaggagtccccttcggtggacacacgactagtggtggttgaacagaccctcgt-tattgtgtgtcatgagctg-ctagggagccctcatcaaaccc-tttgtatcgttttcggacggtgcttcgaccggtcagacgggactacccgctgagttt

>Pyrrhopappus

aaggatcattgtcgaacccaaagcagaacgacccgtgaacatgtaaacacaactgggagtcggaaacattggctccggccttaatccctgccggcatacgtttgtggcatcccgttcgggacgacacgaaagtcatgccggcaccataacaaaccccggcacggaatgtgccaaggaaaacgaaata-gagaagggcatgtccattcgccccgtacgcggtgtgcgtgttgg-tgtgacctccttgaaatcacaaacgactctcggcaacggatatctcggctcacgcatcgatgaagaacgtagcaaaatgcgatacttggtgtgaattgcagaatcccgtgaaccatcgagtttttgaacgcaagttgcgcccaaagccatccggtcgagggcacgcctgcctgggcgtcacgcatcgcgtcgcccccatccatcc-cacgggatgcctg----gcatcggggcggagattggcctcccgtgcttttggtgcggttggcctaaactggagtcaccttcggtggacgcacgactagtggtggttgaacagaccctcgtctgttgtgcgttgtaagctgtgaggtgggcccttgatgaacccctatgtgtcgtcatgtgacgatgcttcgaccggtcaggcgggactacccgctgagttt

>Krigia01

aaggatcattgtcgaacccaaagcagaacgacccgcgaacttgtacccataatcgggagtcagggatattggctctgtccttacaccctgtcggcatatgtttgtggtgccccgttagggatgccacggatgtcatgtcggcgcattaacaaaccccggcacggaatgtgccaaggaaaacaaaaaacgagaaggacgcgtccaattgccccgtttgcggtgtgcttgttgg-cgtggcctccttgaaatcacaaacgactctcggcaacggatatctcggctcacgcatcgatgaagaacgtagcaaaatgcgatacttggtgtgaattgcagaatcccgtgaaccatcgagtttttgaacgcaagttgcgcccaaagccatccggccgagggcacgcctgcctgggcgtcacgcatcgcgtcgcccccatgcatccttatgggatgcttg----gcatcggggcggagattggcctcccgtgcctttggtgtggttggcctaaatcggagtcaccttcggtggacgcacgactagtggtggttgaaaagaccctcgtctgttgtgcgtcgtaagctgtgagggaggcccttcatgaaccccaatgtgtcgtcttgcgacgatgcttcgaccggtcaggcgggactacccgctgagttt

>Krigia02

aaggatcattgtcgaacccaaagcagaacgacccgcgaacttgtacccataatcgggagtcagggatattggctctgtccttacaccctgtcggcatatgtttgtggtgccccgttagggatgccacggatgtcatgtcggcgcattaacaaaccccggcacggaatgtgccaaggaaaacaaaaaacgagaaggacgcgtccaattgccccgtttgcggtgtgcttgttgg-cgtggcctccttgaaatcacaaacgactctcggcaacggatatctcggctcacgcatcgatgaagaacgtagcaaaatgcgatacttggtgtgaattgcagaatcccgtgaaccatcgagtttttgaacgcaagttgcgcccaaagccatccggccgagggcacgcctgcctgggcgtcacgcatcgcgtcgcccccatgcatccttatgggatgcttg----gcatcggggcggagattggcctcccgtgcctttggtgtggttggcctaaatcggagtcaccttcggtggacgcacgactagtggtggttgaaaagaccctcgtctgttgtgcgtcgtaagctgtgagggaggcccttcatgaaccccaatgtgtcgtcttgcgacgatgcttcgaccggtcaggcgggactacccgctgagttt

>Krigia03

aaggatcattgtcgaacccaaagcagaacgacccgcgaacttgtacccataatcgggagtcagggatattggctctgtccttacaccctgtcggcatatgtttgtggtgccccgttagggatgccacggatgtcatgtcggcgcattaacaaaccccggcacggaatgtgccaaggaaaacaaaaaacgagaaggacgcgtccaattgccccgtttgcggtgtgcttgttgg-cgtggcctccttgaaatcacaaacgactctcggcaacggatatctcggctcacgcatcgatgaagaacgtagcaaaatgcgatacttggtgtgaattgcagaatcccgtgaaccatcgagtttttgaacgcaagttgcgcccaaagccatccggccgagggcacgcctgcctgggcgtcacgcatcgcgtcgcccccatgcatccttatgggatgcttg----gcatcggggcggagattggcctcccgtgcctttggtgtggttggcctaaatcggagtcaccttcggtggacgcacgactagtggtggttgaaaagaccctcgtctgttgtgcgtcgtaagctgtgagggaggcccttcatgaaccccaatgtgtcgtcttgcgacgatgcttcgaccggtcaggcgggactacccgctgagttt

>Erigeron01

aaggatcattgtcgaagccaaagcagaacgacccgcgaacatgttaaaacaatcatgccaggatgtattgagcatccgtttggcataccgttgatgtgcctgcctcgttggcccagtgggtcatcttggtggtcgctttga--cgtaacaaaacccaggcacgggatgtgccaaggaactttaaactgaagaattgcccatcccaaagtcccgttcgcggtgtgctcatggggtgtggcatctttgtaatcacaaacgactctcggcaacggatatctcggctcacgcatcgatgaagaacgtagcaaaatgcgatacttggtgtgaattgcagaatcccgtgaaccatcgagtttttgaacgcaagttgcgcccgaagccattcggctgagggcacgtctgcctgggcgtcacgcatcgcgtcgctcccaccatttccttttggattgt-----tggctgggagcggatattggcctcccgttttaaccgagtggttggccaaaataaaagcacctcttgacgggcgcaagactattggtg-----acaaaaccatgaacgttgcgtgtctcgt-----caaaaggttgcttgttatc-cccaacgcgttgtcttttgatgacgcttcgaccggtcaggcgggactacccgctgagttt

>Erigeron02

aaggatcattgtcgaagccaaagcagaacgacccgcgaacatgttaaaacaaccatgccaggatgtgtcgagcattcgttcggcataccgttgatgtgcctgcctagttggccctctgggtcatcttggtggtcgcattga--cgtaacaaaacccaggcacgggatgtgccaaggaacttaaaattgaagaattgcctgtcccatagtcccgttcgcggtgtgctcatggggtctggcatctttgtaatcacaaacgactctcggcaacggatatctcggctcacgcatcgatgaagaacgtagcaaaatgcgatacttggtgtgaattgcagaatcccgtgaaccatcgagtttttgaacgcaagttgcgcccgaagccattcggttgagggcacgtctgcctgggcgtcacgcatcgcgtcgctcccaacatttcctttgggatgct-----tggttgggagcggatattggtctcccgttttcaccgagcggttggccgaaataaaagcacctcttgacgggcgcaagactattggtg-----acaaaaccatgaatgttgcgtgtctcgt-----taaaaggatgcttcttata-cccaacgcgttgttttcttatgacgcttcgaccggtcaggcgggactacccgctgagttt
